# Construction and analysis of telomere-to-telomere genomes for 2 sweet oranges: Longhuihong and Newhall (*Citrus sinensis*)

**DOI:** 10.1093/gigascience/giae084

**Published:** 2024-11-26

**Authors:** Lin Hong, Xin-Dong Xu, Lei Yang, Min Wang, Shuang Li, Haijian Yang, Si-Ying Ye, Ling-Ling Chen, Jia-Ming Song

**Affiliations:** Fruit Tree Research Institute, Chongqing Academy of Agricultural Sciences, Chongqing 401329, China; Integrative Science Center of Germplasm Creation in Western China (CHONGQING) Science City and Southwest University, College of Agronomy and Biotechnology, Southwest University, Chongqing 400715, China; State Key Laboratory for Conservation and Utilization of Subtropical Agro-bioresources, College of Life Science and Technology, Guangxi University, Nanning 530004, China; Fruit Tree Research Institute, Chongqing Academy of Agricultural Sciences, Chongqing 401329, China; Fruit Tree Research Institute, Chongqing Academy of Agricultural Sciences, Chongqing 401329, China; Fruit Tree Research Institute, Chongqing Academy of Agricultural Sciences, Chongqing 401329, China; Fruit Tree Research Institute, Chongqing Academy of Agricultural Sciences, Chongqing 401329, China; Integrative Science Center of Germplasm Creation in Western China (CHONGQING) Science City and Southwest University, College of Agronomy and Biotechnology, Southwest University, Chongqing 400715, China; State Key Laboratory for Conservation and Utilization of Subtropical Agro-bioresources, College of Life Science and Technology, Guangxi University, Nanning 530004, China; State Key Laboratory for Conservation and Utilization of Subtropical Agro-bioresources, College of Life Science and Technology, Guangxi University, Nanning 530004, China; Integrative Science Center of Germplasm Creation in Western China (CHONGQING) Science City and Southwest University, College of Agronomy and Biotechnology, Southwest University, Chongqing 400715, China; State Key Laboratory for Conservation and Utilization of Subtropical Agro-bioresources, College of Life Science and Technology, Guangxi University, Nanning 530004, China

**Keywords:** sweet orange, *Citrus sinensis*, Longhuihong, Newhall, telomere-to-telomere genome, cold tolerance

## Abstract

**Background:**

Sweet orange (*Citrus sinensis* Osbeck) is a fruit crop of high nutritional value that is widely consumed around the world. However, its susceptibility to low-temperature stress limits its cultivation and production in regions prone to frost damage, severely impacting the sustainable development of the sweet orange industry. Therefore, developing cold-resistant sweet orange varieties is of great necessity. Traditional hybrid breeding methods are not feasible due to the polyembryonic phenomenon in sweet oranges, necessitating the enhancement of its germplasm through molecular breeding. High-quality reference genomes are valuable for studying crop resistance to biotic and abiotic stresses. However, the lack of genomic resources for cold-resistant sweet orange varieties has hindered the progress in developing such varieties and researching their molecular mechanisms of cold resistance.

**Findings:**

This study integrated PacBio HiFi, ONT, Hi-C, and Illumina sequencing data to assemble telomere-to-telomere (T2T) reference genomes for the cold-resistant sweet orange mutant “Longhuihong” (*Citrus sinensis* [L.] Osb. cv. LHH) and its wild-type counterpart “Newhall” (*C. sinensis* [L.] Osb. cv. Newhall). Comprehensive evaluations based on multiple criteria revealed that both genomes exhibit high continuity, completeness, and accuracy. The genome sizes were 340.28 Mb and 346.33 Mb, with contig N50 of 39.31 Mb and 36.77 Mb, respectively. In total, 31,456 and 30,021 gene models were annotated in the respective genomes. Leveraging these assembled genomes, comparative genomics analyses were performed, elucidating the evolutionary history of the sweet orange genome. Moreover, the study identified 2,886 structural variants between the 2 genomes, with several SVs located in the upstream, downstream, or intronic regions of homologous genes known to be associated with cold resistance.

**Conclusions:**

The study *de novo* assembled 2 T2T reference genomes of sweet orange varieties exhibiting different levels of cold tolerance. These genomes serve as valuable foundational resources for genomic research and molecular breeding aimed at enhancing cold tolerance in sweet oranges. Additionally, they expand the existing repository of reference genomes and sequencing data resources for *C. sinensis*. Moreover, these genomes provide a critical data foundation for comparative genomics analyses across different plant species.

## Introduction

The sweet orange (*Citrus sinensis* Osbeck; NCBI:txid2711), a member of the Rutaceae family and *Citrus* genus, is globally recognized as one of the most commercially valuable fruits due to its high content of bioactive compounds such as flavonoids, phenolic acids, alkaloids, carotenoids, and limonoids, which provide significant anti-inflammatory, anticancer, and antioxidant benefits. These properties make sweet oranges widely applicable in agriculture, food, and medicine [1, 2]. Sweet oranges can be consumed fresh or juiced and are generally categorized into 3 types: blond oranges, navel oranges, and blood oranges [3]. Navel oranges, distinguished by a secondary fruitlet (resembling a navel) at the bottom of the fruit, have a high mutation rate that has facilitated their proliferation, with numerous cultivars being bred and disseminated worldwide [4]. To date, there are over 190 known varieties, with commonly cultivated types including “Washington,” “Newhall,” “Chislett,” “Powell,” “Lane Late,” “Barnfield,” and “Cara Cara” [4, 5]. Somatic mutation-based bud sport breeding has become a vital method for developing new fruit tree varieties. It is estimated that around 80% of sweet orange cultivars originate from somatic mutations, with sweet oranges being cultivated in 114 countries globally. Moreover, the apomictic nature of sweet oranges allows for strict clonal propagation under natural conditions, making them an ideal species for studying somatic mutations [6, 7].

The genome is considered a foundational element in biological research. In 2000, the first plant genome, the *Arabidopsis thaliana* genome, was published by scientists [8]. Over the past 2 decades, with rapid advancements in sequencing technology, assembly algorithms, genetics, and bioinformatics [9], nearly 900 plant species’ genomes have been published to date (based on data from NCBI and GWH). In 2012, the first genome of the sweet orange was sequenced. To reduce assembly complexity, double haploid sequencing was utilized by researchers, resulting in the successful mapping of 87% of the sweet orange genome. The total assembly size was determined to be 320 Mb, with a contig N50 of 49.89 Kb and a scaffold N50 of 1.69 Mb, and 29,445 protein-coding genes were identified [10]. In 2014, the genome sequences of 7 citrus species, including clementine, mandarin, pummelo, sweet orange, and sour orange, were completed. Higher-quality genomes for the *Citrus* genus were produced, significantly improving upon the previously published sweet orange genome (contig N50 = 119 Kb, scaffold N50 = 6.8 Mb). Evolution and domestication of the *Citrus* genus were explored, indicating that the cultivated pummelo originated from the ancestral species *Citrus maxima*, while the cultivated mandarin was derived from *Citrus reticulata* with introgressions from *C. maxima* [11]. In 2017, single-molecule sequencing technology was employed to complete the genomes of 4 representative citrus species, achieving a contig N50 of 2.2 Mb, which is over 18 times greater than previously reported citrus genomes. Comparative genomic analysis revealed that the citrus-specific genomic regions primarily consist of repeat sequences and genes of unknown function, with approximately one-fifth of the genes having known functions and being enriched in biological pathways related to resistance, proteolysis, and pectin degradation [12]. In 2021, a bud sports population was combined with genomics strategies to improve the scope and accuracy of somatic mutation predictions. The genomes of a double haploid sweet orange and 6 diploids were first assembled, leaving an average of only 3 gaps per chromosome across the 9 chromosomes [7]. In 2023, a genomic map of the Citrus subfamily was constructed, with *de novo* assembly of the genomes of 12 *Citrus* genus and closely related species. The contig N50 of these genomes ranged from 1.6 to 16.8 Mb, with assembly sizes ranging from 217.8 to 419.1 Mb, thus covering over 90% of their estimated genome sizes. The number of annotated gene models in these genomes ranged from 22,907 to 31,413 [13]. In 2023, a complete and haplotype-resolved telomere-to-telomere (T2T) genome of the lemon variety perfume lemon was assembled by researchers. The assembled genome is 633.0 Mb in size, with a contig N50 of 35.6 Mb and zero gaps, achieving a fully gap-free assembly. Further multiomics analyses identified candidate genes associated with the biosynthesis of flavor compounds and resistance to Huanglongbing (HLB), providing a foundation for accelerating molecular breeding programs and the discovery of functional genes [14]. In 2024, a study has reported the T2T genome of the “Nei Xiu,” a bud mutation originating from Tarocco blood orange [15].

**Figure 1:**
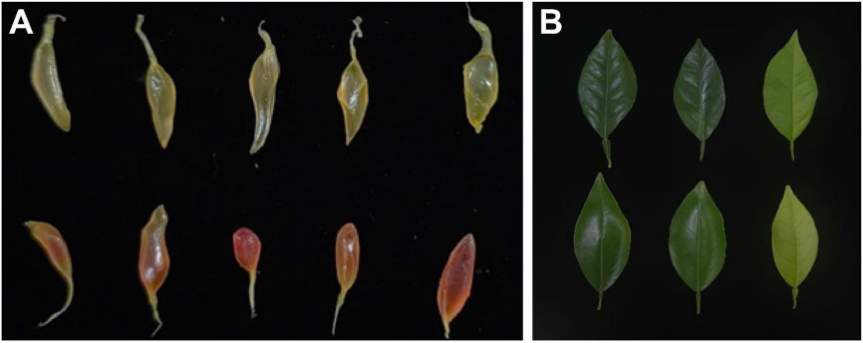
Morphological characteristics of *C. sinensis* LHH and Newhall. (A) Comparison of juice sac characteristics between navel orange cultivar Newhall (top) and LHH (bottom). (B) Comparison of leaf characteristics between navel orange cultivar LHH (top) and Newhall (bottom).

“Longhuihong” (LHH) is a bud mutation of the “Newhall” navel orange, known for its enhanced cold tolerance. The low-temperature stress during winter stimulates the synthesis of anthocyanins in LHH fruits, leading to visibly red juice sacs (Fig. 1A). The leaves display distinct curling and prominent veins, making its variation characteristics highly noticeable (Fig. 1B). Variety trials have demonstrated that LHH possesses several agronomic traits superior to those of Newhall, indicating that it may harbor multiple advantageous genotypes with significant development potential. However, the lack of available genomic resources for LHH currently limits the exploration and utilization of the genetic basis behind its desirable traits—such as cold tolerance, high photosynthetic efficiency, and high anthocyanin content. This poses a significant challenge to molecular breeding efforts for sweet oranges.

This study leveraged state-of-the-art sequencing technologies, including PacBio High Fidelity (HiFi), Oxford Nanopore Technologies Ultra Long (ONT UL), high-throughput chromosome conformation capture (Hi-C), and Illumina sequencing data, to report the T2T genome assembly of navel orange varieties, LHH and Newhall. These assemblies provide valuable genomic resources for pangenome studies, functional gene identification, and molecular breeding of sweet oranges. Furthermore, by utilizing the 2 T2T genomes, we elucidated the evolutionary history of the sweet orange genome and characterized the genomic variations between the 2 varieties. This research offers critical data support for future genomic studies and breeding programs aimed at enhancing cold tolerance in sweet oranges.

## Materials and Methods

### Sample collection

Samples of the navel orange varieties LHH and Newhall were collected from an experimental orchard specializing in sweet orange varieties in Fengming Town, Yunyang County, Chongqing, at an elevation of 750 m. Fresh leaf samples were obtained from 4-year-old trees that had been grafted onto trifoliate orange rootstocks and planted at a density of 5 by 3 m. These samples were then used for subsequent DNA extraction, library construction, and sequencing.

### DNA isolation, library construction, and sequencing

Healthy, juvenile leaves were carefully selected and their surfaces were thoroughly rinsed with deionized water. After gently drying the leaves to eliminate surface moisture, we precisely cut the tissue into 50- to 100-mg fragments. These fragments were then placed into preprepared 2-mL cryovials and promptly frozen in liquid nitrogen for 3 to 4 hours. Following this, the samples were securely stored at −80°C for future DNA and RNA extraction. High-quality genomic DNA was extracted from the leaves of *C. sinensis* LHH and Newhall using the cetyl-trimethylammonium bromide (CTAB) method [16]. Qualified genomic DNA samples were then used for the construction of sequencing libraries. The size and quantity of library fragments were assessed using Qseq400 and Qubit, respectively, ensuring library quality. The qualified libraries were subsequently immobilized onto sequencing chips via bridge PCR methods. Illumina sequencing was eventually conducted, performing 150-bp paired-end sequencing on an Illumina sequencer. For PacBio Circular Consensus Sequencing (CCS), long-fragment library construction was carried out using genomic DNA extracted from leaf samples, following the manufacturer’s instructions. The genomic DNA was then sheared into 15-kb fragments. Sequencing of the constructed library was executed on the PacBio Sequel II platform (RRID:SCR_017990). After sequencing, low-quality reads and sequencing adapters were removed to obtain clean subreads. Regarding ONT UL sequencing, library preparation was conducted using the SQK-ULK001 kit, following the manufacturer’s instructions. Libraries were purified and sequenced using a PromethION sequencer. The Hi-C experiment protocol was as follows: (i) collect 1 g of tender leaves, rinse them thoroughly in icy water, and gently blot them dry with absorbent paper. (ii) Using scissors, carefully mince the sample tissue and place it into a 50-mL centrifuge tube. (iii) Transfer the minced tissue into a 50-mL centrifuge tube containing 35 mL NIB Buffer. (iv) Introduce 35 µL PMSF, 35 µL β-mercaptoethanol, and 2 mL 36% formaldehyde into the mixture. (v) Allow the mixture to react for 90 minutes on a vertical rotator. (vi) Add 2.5 mL of 2 M glycine and gently agitate the solution, either manually or on a shaker, for a minimum of 5 minutes to halt the reaction. (vii) Filter out the excess liquid and rinse the sample thoroughly with sterile water until all foam disappears. (viii) Blot the sample dry with absorbent paper, place it in a 50-mL centrifuge tube, and promptly freeze it in liquid nitrogen for future use. Upon passing quality checks, Illumina-based high-throughput sequencing was performed, generating 150-bp paired-end reads.

### Genome survey

Before genome assembly, a 21-mer–based survey was conducted on the LHH and Newhall genomes to estimate genome size, heterozygosity, repeat content, and ploidy level, providing critical reference information for subsequent assembly efforts. Jellyfish (v2.3.1) [17] was employed to count the 21-mers from the Illumina paired-end sequencing data. Following this, GenomeScope (RRID:SCR_017014) (v2.0) [18] was used to analyze the 21-mer frequency distributions. Smudgeplot (v0.2.5) [18] was utilized to estimate genome ploidy.

### Genome assembly and assessment

Based on the PacBio HiFi, ONT UL, and Hi-C sequencing data obtained from whole-genome sequencing, the LHH and Newhall navel orange genomes were *de novo* assembled. Hifiasm (v0.19) [19] was utilized for the initial assembly of the LHH and Newhall genomes, with ONT UL sequencing data integrated using the “–ul” parameter to enhance the assembly process. Subsequently, the initially assembled contigs were aligned to the NCBI NT database, the mitochondrial database, and the plastid database to filter out contaminants, mitochondrial sequences, and plastid sequences, resulting in clean contig sequences. Leveraging Hi-C contact signals, LACHESIS (RRID:SCR_017644) [20] was employed to group, order, and orient the contigs with the following parameters: “CLUSTER_MIN_RE_SITES = 100; CLUSTER_MAX_LINK_DENSITY = 2; ORDER_MIN_N_RES_IN_TRUNK = 15; ORDER_MIN_N_RES_IN_SHREDS = 15.” Subsequently, Juicebox (RRID:SCR_021172) [21] was used to manually inspect and adjust the scaffolding results from LACHESIS. TGS-Gapcloser (RRID:SCR_017633) (v1.2.1) [22] was utilized to close gaps in the anchored reference genome using ONT UL sequencing data. Ultimately, 8 and 7 gap-free chromosome assemblies were obtained for the LHH and Newhall sweet orange varieties, respectively. Potential telomeric repeat units within the genome were identified using TIDK. Subsequently, potential telomeric sequences were located with FindTelomeres (RRID:SCR_024403) based on these repeat units, enabling the acquisition of both telomeric positions and sequences. CentIER (v3.0) [23] was used to identify potential centromere regions. After completing the assembly of the 2 sweet orange genomes, various strategies were employed to verify their completeness and accuracy. The BUSCO (RRID:SCR_015008) [24] dataset, embryophyta_odb10, comprising 1,614 genes, was mapped to the genomes. Minimap2 (RRID:SCR_018550) (v2.26-r1175) [25] was then used to map the HiFi and ONT UL reads to the genomes. LTR_retriever (RRID:SCR_017623) (v2.9.8) [26] was employed to calculate the LTR Assembly Index (LAI) based on long terminal repeat (LTR) annotations, to evaluate the assembly quality of repetitive sequences in both genomes [27]. Merqury (RRID:SCR_022964) (v1.3) [28] was utilized to calculate the assembly consensus quality value (QV) for the 2 genomes.

### Genome annotation

*De novo* prediction of repetitive sequences was performed using RepeatModeler (RRID:SCR_015027) (v2.0.1) [29], while LTR_retriever (v2.9.0) [26] was utilized for *de novo* prediction of LTRs. The results from both tools were merged with Repbase (RRID:SCR_021169) [30] and deduplicated to construct a comprehensive repeat library for the 2 navel orange genomes. This repeat library was then used by RepeatMasker (RRID:SCR_012954) (v4.1.2) [31] for genome-wide repeat annotation, resulting in masked versions of the genomes. Augustus (RRID:SCR_008417) (v3.1.0) [31] and SNAP (RRID:SCR_007936) [32] were used for *de novo* gene model predictions for the LHH and Newhall genomes; GeMoMa (RRID:SCR_017646) (v1.7) [33] was employed for gene model predictions based on homologous genes from closely related species. In this study, 2 strategies were used for transcript-based gene model predictions. The first strategy involved processing RNA sequencing (RNA-seq) data with HISAT (RRID:SCR_015530) (v2.1.0) [34] and StringTie (RRID:SCR_016323) (v2.1.4) [35], followed by gene model prediction using GeneMarkS-T (RRID:SCR_017648) (v5.1) [36]. The second strategy used Trinity (RRID:SCR_013048) (v2.11) [37] for transcript assembly, and the assembled transcripts were then fed into PASA (RRID:SCR_014656) (v2.4.1) [38] for gene prediction. Finally, EVidenceModeler (RRID:SCR_014659) (v1.1.1) [39] was used to merge the gene model predictions from the 3 methods, and PASA (v2.4.1) [38] was used to adjust the merged annotations. HiCExplorer (RRID:SCR_022111) (v3.7.4) [40] was used to analyze A/B compartments from Hi-C data. Additionally, the NR, eggNOG (RRID:SCR_002456) [41], Gene Ontology (GO), KEGG [42], TrEMBL [43], KOG, SWISS-PROT [43], and Pfam (RRID:SCR_004726) [44] databases were employed to perform functional annotation of these gene sequences. tRNAscan-SE (RRID:SCR_008637) (v1.3.1) [45] was utilized for the recognition of transfer RNA (tRNA), and Barrnap (RRID:SCR_015995) (v0.9) was used for the prediction of ribosomal RNA (rRNA). MicroRNAs (miRNAs), small nucleolar RNAs (snoRNAs), and small nuclear RNAs (snRNAs) were identified based on the Rfam (RRID:SCR_007891) (v14.5) [46] database using Infernal (RRID:SCR_011809) (v1.1) [47].

### Comparative genomics and evolutionary analysis

MAFFT (RRID:SCR_011811) (v7.205) [48] was used to generate multiple sequence alignments (MSAs) for these single-copy orthologs, and Gblocks (RRID:SCR_015945) (v0.91b) [49] was employed to remove highly variable regions from the MSA. The MSA of single-copy orthologs was then concatenated. IQ-TREE (RRID:SCR_017254) (v1.6.11) [50] was used to construct a maximum likelihood (ML) phylogenetic tree based on the MSA, applying the model “JTT+F+I+G4” and a bootstrap value of 1,000. OrthoFinder (RRID:SCR_017118) (v2.4) [51] was used for gene family clustering. Computational Analysis of gene Family Evolution (CAFÉ: RRID:SCR_005983) (v4.2) [52] was employed to estimate the number of gene family members in the ancestral branches using the birth–death (λ) model, based on the evolutionary tree with divergence times and gene family clustering results. This approach allowed for the prediction of gene family contraction and expansion relative to the ancestors. In this study, a gene family was considered to have undergone significant expansion or contraction if both the family-wide *P* values and Viterbi *P* values were less than .05. The estimation of divergence times was performed using the mcmctree module in PAML (RRID:SCR_014932) (v4.9i) [53].

### Detection of variations

In this study, MUMandCo (v3.8) [54] was utilized to detect and classify structural variations from Mummer’s alignment results. Based on alignment results from Mummer (RRID:SCR_018171) (v4.0.0rc1) [54], ANNOVAR (RRID:SCR_012821) [55] was used to annotate the detected structural variations relative to gene positions. GenomeSyn (v1.2.7) [55] was used to integrate and present these data and perform genome collinearity analysis. BLASTp (RRID:SCR_001010) (v2.5.0) [56] was used to map the collected cold tolerance–related genes from rice to the Newhall genome. Minimap2 (v2.26-r1175) [25] was used to align the PacBio HiFi reads of the LHH genome to the Newhall genome. Subsequently, DeepVariant (v1.6.1) [57] was employed to perform single-nucleotide polymorphism (SNP) detection from the alignment results. The heterozygous SNPs identified in the SNP calling results were then filtered out, resulting in the final dataset used for presentation.

## Results

### Genome sequencing

For the LHH and Newhall varieties, 28.04 Gb (∼71×) and 26.07 Gb (∼79×) of PacBio HiFi reads, 20.58 Gb (∼51×) and 24.49 Gb (∼61×) of ONT UL reads, 18.25 Gb (∼43×) and 17.18 Gb (∼46×) of Illumina PE reads, and 50.69 Gb (∼136×) and 50.43 Gb (∼146×) of Hi-C reads were obtained, respectively (Table 1).

**Table 1:** Statistics of the clean data of the *C. sinensis* LHH and Newhall genomes

	*C. sinensis* LHH			*C. sinensis* Newhall		
Type	Total data (Gb)	Sequence depth (×)	Average size (bp)	Total data (Gb)	Sequence depth (×)	Average size (bp)
PacBio	28.04	71	13,757	26.07	79	13,965
ONT	20.58	51	98,866	24.49	61	95,293
Hi-C	50.69	136	150	50.43	146	150
Illumina	18.25	43	150	17.18	46	150

### Genome survey

**Figure 2:**
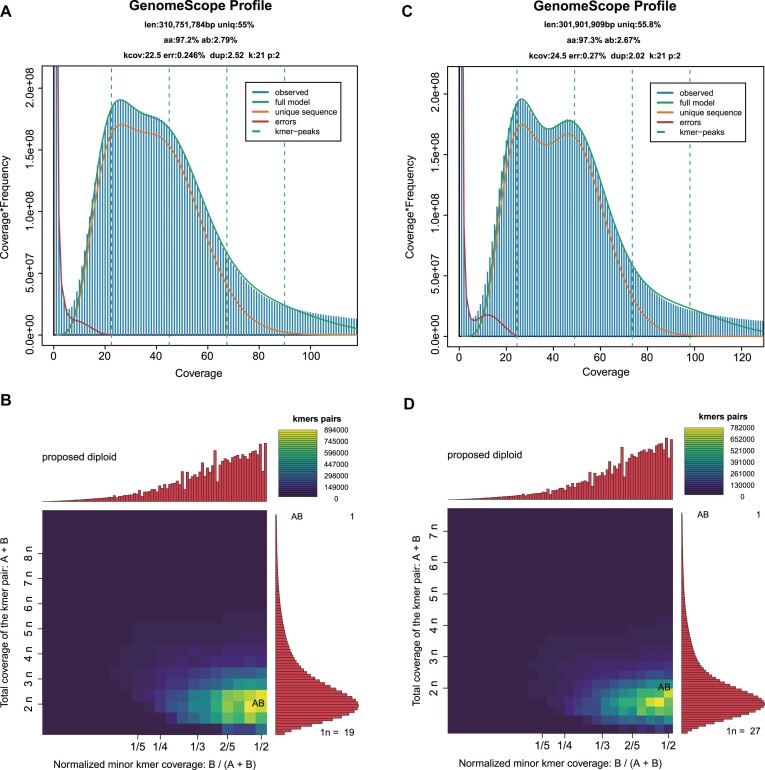
The genomic feature survey of *C. sinensis* LHH and Newhall genomes based on 21-mer. (A) The 21-mer spectra for *C. sinensis* LHH. (B) Smudgeplots for *C. sinensis* LHH. (C) The 21-mer spectra for *C. sinensis* Newhall. (D) Smudgeplots for *C. sinensis* Newhall.

The survey results revealed that the estimated genome sizes for LHH and Newhall were 310.75 Mb and 301.9 Mb, respectively, with heterozygosities of 2.79% and 2.67%. Both genomes exhibited high levels of heterozygosity and similar repeat content, 45.0% and 44.8%, respectively (Fig. 2A, C). Ploidy estimation results indicated that both LHH and Newhall genomes are heterozygous diploids, consistent with previously reported sweet orange genomes (Fig. 2B, D).

### Genome assembly and assessment

**Figure 3:**
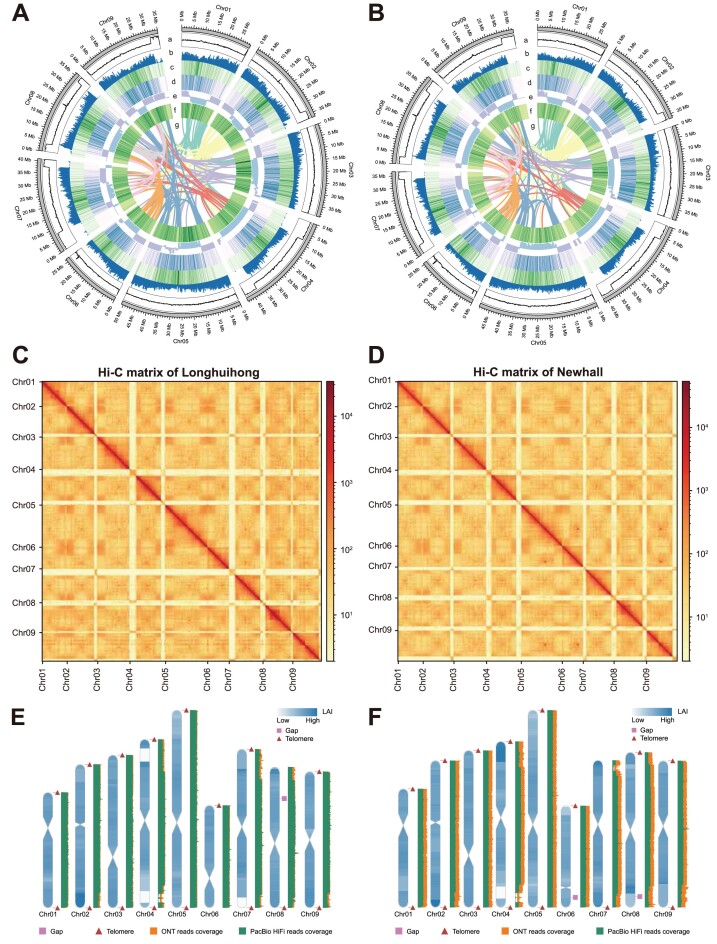
Assembly and assessment of the *C. sinensis* LHH and Newhall genomes. (A, B) Circos plot of the genomic landscape of the *C. sinensis* LHH genome (A) and *C. sinensis* Newhall genome (B). The circos plots show, outermost to innermost, GC content (a), gene density (b), LTR/*Gypsy* density, LTR/*Copia* density (d), A/B compartment (e), DNA transposon density (f), and syntenic regions within the genome (g). (C, D) Hi-C contact matrix of LHH genome (C) and Newhall genome (D). (E, F) The distribution landscape of centromeres, telomeres, LAI, HiFi reads coverage, and ONT reads coverage in the LHH genome (E) and Newhall genome (F).

Initial assembly results indicated that the LHH and Newhall genomes contained 160 and 141 contigs, respectively, with total lengths of 372.71 Mb and 370.23 Mb and contig N50 values of 36.91 Mb and 36.77 Mb (Table 2). After filtering contaminants from the preliminary assembly, Hi-C data were used to anchor the clean contigs to the chromosome level for the LHH and Newhall genomes (Fig. 3C, D). The statistics revealed that 338.55 Mb and 337.47 Mb of the clean contigs for LHH and Newhall, respectively, were anchored to 9 pseudochromosomes, with anchoring rates of 99.49% and 97.44% (Supplementary Table S1). Telomeric sequences were detected at both ends of 7 chromosomes in each genome, while the remaining chromosomes exhibited telomeric sequences at one end. Centromeric sequences were identified on all chromosomes in both genomes, indicating that both assemblies achieved a T2T level [58] (Fig. 3E, F, Supplementary Tables S2, 3). The total lengths of the T2T genomes for LHH and Newhall were 340.28 Mb and 346.33 Mb, respectively, consistent with the genome sizes estimated from the genome surveys.

**Table 2:** Assembly statistics of the *C. sinensis* LHH and Newhall genomes.

	*C. sinensis* LHH		*C. sinensis* Newhall	
Items	Contig	Scaffold	Contig	Scaffold
Sequence Number	24	9	141	9
Assembly size (Mb)	361.41	338.51	370.23	334.63
Longest SeqLen (Mb)	50.84	50.84	49.40	49.40
Average SeqLen (Mb)	15.06	37.61	2.62	37.18
N50 (Mb)	36.91	39.31	36.77	38.85

The BUSCO assessment results indicate that the completeness of the LHH and Newhall genomes is 99.07% and 99.19%, respectively (Supplementary Fig. S1, Supplementary Table S4). Additionally, the Core Eukaryotic Genes Mapping Approach (CEGMA) [59] was utilized for further evaluation, indicating completeness scores of 99.13% for the LHH genome and 99.56% for the Newhall genome. The mapping statistics showed that the HiFi reads had mapping rates of 99.11% for LHH and 99.58% for Newhall, with coverages of 99.98% and 99.99%, as well as average read depths of 71× and 68×, respectively. For the ONT UL reads, the mapping rates were 96.87% for LHH and 97.05% for Newhall, with coverages of 99.96% and 99.77%, as well as average read depths of 51× and 61×, respectively (Supplementary Table S5). These results underscore the high completeness of both genome assemblies. The Hi-C contact matrices for both genomes displayed smooth and continuous Hi-C signals, confirming the correct order and orientation of the genome assemblies (Fig. 3C, D). Based on the LAI, the genome assembly quality assessment shows that the LAI values for the LHH and Newhall genomes are 20.39 and 20.09, respectively, both meeting the standards for gold reference genomes [27]. Further analysis of LAI across different chromosomal regions indicated that most regions of the T2T genomes exhibited high LAI, and the mapping results for HiFi and ONT UL reads demonstrated uniform coverage in these areas (Fig. 3E, F). However, some regions showed relatively lower LAI, likely due to lower HiFi read coverage in those areas. Nevertheless, ONT UL reads successfully covered and filled these regions (Fig. 3E, F). Finally, the results of calculating the assembly consensus QV for the LHH and Newhall genomes showed that the QV score for LHH was 46.64, while for Newhall, it was 38.89. This indicates that the accuracies of the 2 genomes are 99.99% and 99.9%, respectively, demonstrating high accuracy [28].

### Genome annotation

The study initially performed repeat annotation on both genomes and masked the repetitive regions to improve the efficiency and accuracy of genome structural annotation. The results indicated that 205,650 and 197,501 transposable elements (TEs) were identified in the LHH and Newhall genomes, with total lengths of 115.70 Mb and 116.79 Mb, respectively, accounting for 34.00% and 33.72% of each genome (Supplementary Table S6). Among these, LTR/Gypsy elements were the most abundant, comprising 11.37% and 12.69% of the LHH and Newhall genomes, respectively (Supplementary Table S6). Furthermore, class I transposons, or retrotransposons, were found to be more prevalent than class II transposons, or DNA transposons, in both genomes, consistent with patterns observed in other plant species [60]. A comparison with the distribution of TEs revealed a high density of TEs in the central regions of the chromosomes, corresponding to the B compartment areas, which are characterized by low gene density, low transcriptional activity, and high chromatin condensation (Fig. 3A, B).

**Figure 4:**
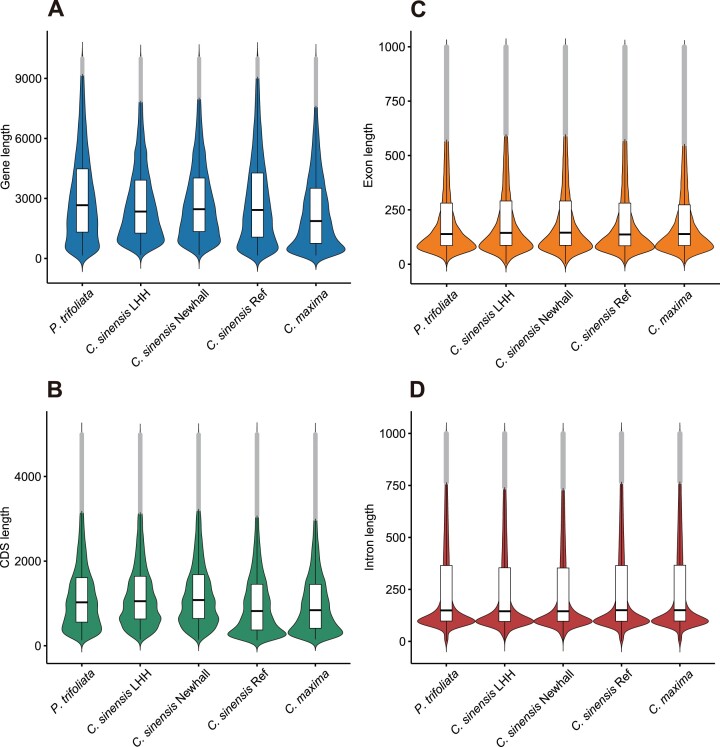
Statistical analysis and assessment of the annotation results for the *C. sinensis* LHH and Newhall genome. Comparison of gene length (A), CDS length (B), exon length (C), and intron length (D) distribution between the genomes of LHH, Newhall, and closely related species. *C. sinensis* Ref refers to the sweet orange reference genome obtained from the NCBI database, with the corresponding RefSeq ID GCF_022201045.2.

Subsequently, a comprehensive annotation strategy was implemented in the study, integrating *ab initio*, homology-based, and transcript evidence-based approaches to annotate gene structures in the repeat-masked LHH and Newhall genomes (Supplementary Table S7). The analysis identified 31,456 and 30,021 gene models in the LHH and Newhall genomes, respectively (Supplementary Fig. S2, Supplementary Table S7). Further statistical analysis of these gene models revealed that the distribution of annotated genes, coding sequences (CDS), exons, and intron lengths in both genomes were comparable to those reported in previously described sweet orange and closely related species genomes [61–63]. This consistency underscores the accuracy and reliability of the annotated gene models (Fig. 4).

Functional annotation results show that 96.66% and 97.6% of the genes in the LHH and Newhall genomes, respectively, were successfully annotated using these resources (Table 3). Moreover, the study focused on identifying noncoding RNAs in both genomes. The analysis revealed that 411 and 418 tRNAs, 714 and 3,920 rRNAs, and 167 and 166 miRNAs were annotated in the LHH and Newhall genomes, respectively (Supplementary Table S8).

**Table 3:** Statistics of function annotation results for *C. sinensis* LHH and Newhall.

	*C. sinensis* LHH		*C. sinensis* Newhall	
Database	Annotated number	Annotated ratio (%)	Annotated number	Annotated ratio (%)
GO	24,741	78.65	23,881	79.55
KEGG	23,558	74.89	22,846	76.1
KOG	16,155	51.36	15,641	52.1
Pfam	25,420	80.81	24,722	82.35
SWISS-PROT	21,482	68.29	20,922	69.69
TrEMBL	30,266	96.22	29,198	97.26
eggNOG	25,306	80.45	24,461	81.48
NR	30,106	95.71	29,078	96.86
Total	30,404	96.66	29,299	97.60

### Comparative genomics and evolutionary analysis

**Figure 5:**
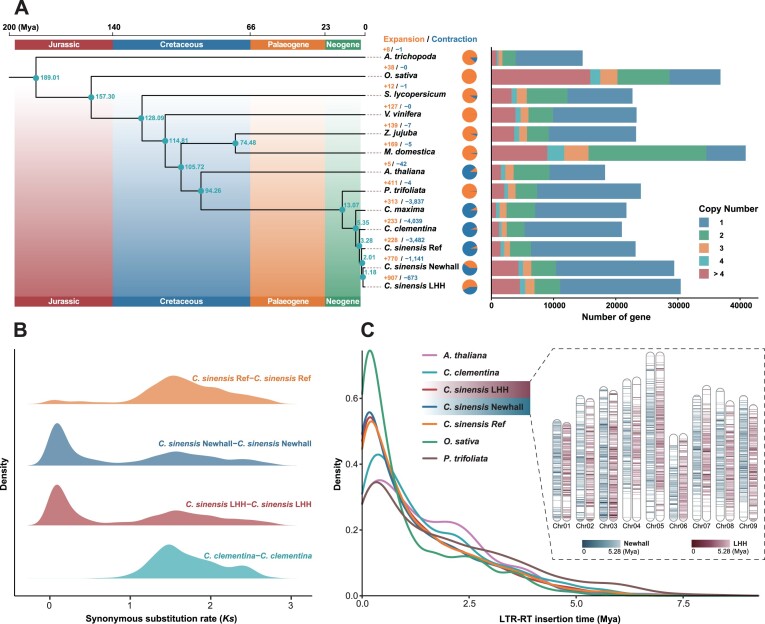
The evolutionary history of *C. sinensis* LHH and Newhall genomes. (A) Phylogenetic tree of *C. sinensis* LHH and Newhall along with 11 other plant genomes. Pie charts depict the expansion and contraction of gene families, with orange indicating expansion and blue indicating contraction. Corresponding values are displayed in matching colors above each chart. Nodes on the evolutionary tree are labeled with estimated divergence times (in Mya). The number of gene copies in each species’ genome is shown on the right side. (B) The distribution of *Ks* values illustrates WGT events in the evolution of the *C. sinensis* LHH and Newhall genomes. (C) The distribution of full-length LTR-RT insertions in LHH and Newhall genomes. The density plot illustrates the temporal distribution of full-length LTR-RT insertions in plant genomes, including LHH and Newhall genomes. The chromosome heatmap provides a comprehensive view of the spatial distribution and temporal information of full-length LTR-RT insertions on each chromosome of LHH and Newhall genomes.

Based on the T2T genomes of LHH and Newhall, the study aimed to elucidate the evolutionary history of the navel orange genome. A total of 392,184 genes from 13 species (*Amborella trichopoda, Oryza sativa, Solanum lycopersicum, Vitis vinifera, Ziziphus jujuba, Malus domestica, Arabidopsis thaliana, Poncirus trifoliata, Citrus maxima, Citrus clementina, Citrus sinensis* Ref, *Citrus sinensis* LHH, and *Citrus sinensis* Newhall) were clustered into 43,324 gene families. Among these, 3,331 gene families were found to be common to all 13 species. Specifically, 30,442 and 29,395 genes from LHH and Newhall were clustered into 22,554 and 22,022 gene families, respectively, with 282 and 122 gene families being unique to each (Supplementary Fig. S3A, Supplementary Table S9). Analysis of 5 species within the *Citrus* genus revealed that 13,531 gene families were common across all 5 species, while LHH and Newhall had 282 and 122 unique gene families, respectively (Supplementary Fig. S3B). A review of gene copy numbers indicated that the proportion of genes with varying copy numbers in the LHH and Newhall genomes was comparable, with single-copy genes being the most prevalent (Fig. 5A).

Through gene family clustering analysis, 926 single-copy orthologs were identified across the 13 species. Based on the phylogenetic analysis of these single-copy orthologs, the 5 *Citrus* species, along with the closely related species *P. trifoliata*, clustered within the same branch, demonstrating high accuracy and reliability of the phylogenetic tree. Furthermore, we estimated that the divergence time between *P. trifoliata* and the 5 *Citrus* species occurred approximately 13.07 million years ago (Mya), during the Miocene epoch (Fig. 5A).

The analysis of gene family expansion and contraction across the genomes revealed that LHH and Newhall have 907 and 770 expanded gene families, respectively. Additionally, LHH has 673 contracted gene families, while Newhall has 1,141 contracted gene families (Fig. 5A). The distribution of the synonymous substitution rate (*Ks*) indicates that the *C. sinensis* LHH and Newhall genomes have undergone an ancient whole-genome triplication (WGT) event (*Ks* = 1.56), which is consistent with previous findings [10] (Fig. 5B). The insertion of long terminal repeat retrotransposons (LTR-RTs) plays a crucial role in the evolution of plant genomes [64, 65]. The study analyzed the insertion times and genomic positions of LTR-RTs identified in LHH and Newhall. In the 3 *C. sinensis* genomes, a higher density of recent LTR-RT insertions was observed compared to its closely related species, *P. trifoliata*. This increased density may have contributed to the larger genome size of *C. sinensis* relative to *P. trifoliata* [66]. These LTR-RT insertions were widespread across sweet orange chromosomes and also found within euchromatic regions, likely due to the ongoing amplification of LTR-RTs [60]. Furthermore, certain regions exhibited a high-density distribution of recent insertions, suggesting the presence of active LTR-RTs in these areas (Fig. 5C).

### Genomic variation between the LHH and Newhall genomes

**Figure 6:**
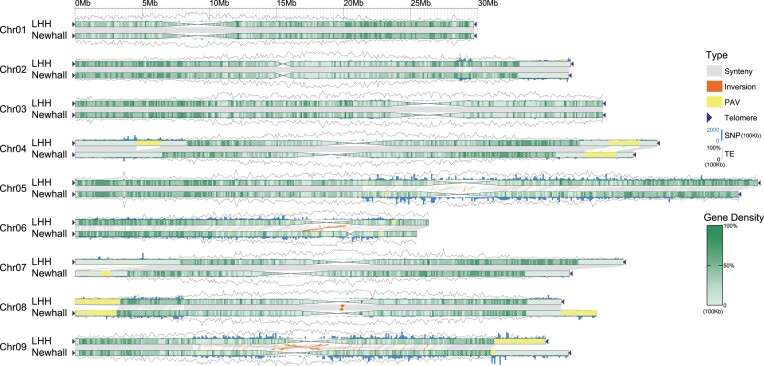
Variations between LHH and Newhall genomes. Collinear regions are connected by gray lines, while inversion regions are connected by orange lines. PAV regions are represented by yellow blocks. Black triangles indicate detected telomere repeat sequences. Gene density heatmaps are plotted on each chromosome with a 100-kb unit. SNP and TE percentage distribution are plotted above or below each chromosome in 100-kb units.

To elucidate the sequence differences between the genomes of navel orange LHH and Newhall, and to provide a foundational dataset for understanding their phenotypic diversity, collinear regions and variations between the 2 genomes were identified. Collinearity analysis was conducted between the genomes, and SNPs, as well as presence/absence variations (PAVs), were called. Subsequently, the results of genomic colinearity analysis revealed 6,075 collinear blocks between the 2 genomes. The lengths of these blocks amounted to 289.07 Mb in the LHH genome and 289.54 Mb in the Newhall genome. These blocks accounted for 372.71 Mb and 370.23 Mb, or 77.56% and 78.21% of the respective genomes, indicating that most regions in both genomes were conserved. The density of SNPs showed differential distributions across the chromosomes of the LHH and Newhall genomes. Chromosomes 3, 5, and 9 were observed to have an abundance of SNPs, with chromosome 9 containing the highest number at 410,667 (Fig. 6). Additionally, these 3 chromosomes exhibited more PAVs compared to the other chromosomes. Genome structural variations (SVs) can affect gene expression through various mechanisms [67] and have been reported to contribute to phenotypic diversity in eukaryotes, driving the diversity of functional genes in crops [68]. SVs are considered to have a greater impact on gene expression and protein function compared to SNPs [69]. A total of 2,886 SVs were detected between the LHH and Newhall genomes, with insertions and deletions being the most common types of structural variations, numbering 1,383 and 1,313, respectively (Supplementary Fig. S5A). Among these structural variations, 164 were further identified as haplotype-specific structural variations. The distribution and types of SVs on different chromosomes revealed that chromosomes 5, 6, and 9 had a higher number of structural variations. Genome-wide analysis indicated that insertions and deletions were the predominant types of structural variations. The positions of these SVs relative to gene models in the Newhall genome were further annotated. The results revealed significant differences in the distribution of SVs at various relative positions across the chromosomes. Chromosomes 5, 6, and 9 had the highest proportion of SVs located in exon regions, with 338,203, and 220 SVs, respectively, accounting for 31.98%, 25.66%, and 31.38% of the total SVs on each chromosome (Supplementary Fig. S5B). These findings indicate substantial genomic structural diversity between the LHH and Newhall genomes. The extensive variation information within these 2 genomes offers valuable resources for identifying and utilizing alleles associated with superior agronomic traits.

Previously reported rice cold tolerance–related genes were collected and mapped onto the Newhall genome to identify homologous genes. By integrating information on detected SVs, several homologous genes were found to have SVs in their upstream, downstream, or intronic regions. These SVs may be linked to the differences in cold resistance between Newhall and LHH (Supplementary Table S10).

### Reuse potential

This study successfully assembled 2 T2T reference genomes for navel orange, providing high-quality genomic resources for future multiomics and molecular biology research in sweet orange. These reference genomes will significantly enhance the utilization of multiomics data and improve the accuracy of downstream analyses.

The 2 navel orange varieties selected for this research exhibit notable differences in important agronomic traits such as flesh color, photosynthetic efficiency, and cold tolerance. Future studies can leverage the high-quality reference genomes generated in this study to construct a pangenome for sweet orange. By incorporating phenotypic information from representative materials, further screening of the various types of variations identified between the LHH and Newhall genomes in this study, including SNPs, SVs, and PAVs, can be undertaken. Subsequent *in vivo* and *in vitro* experiments can validate the impact of these structural variations on phenotypes, allowing for the characterization of genes related to important agronomic traits and the understanding of how these variations influence gene function.

Additionally, forthcoming research can use the 2 T2T genomes as references to analyze population resequencing data of sweet orange. Genome-wide association studies based on SNPs, PAVs, and SVs can then be employed to identify and exploit genomic variations with substantial effects.

Finally, high-quality genomes are crucial for accurate gene prediction. The T2T genomes of the 2 navel orange varieties will facilitate further improvement in gene annotation quality. Future studies can refine sweet orange gene annotations using full-length transcriptome sequencing and manually validate and correct the annotation result.

## Discussion

In this study, we performed *de novo* assembly and annotation of the genomes of 2 navel orange varieties that differ in key agronomic traits such as flesh color, photosynthetic efficiency, and cold tolerance. We utilized a comprehensive approach integrating PacBio HiFi, ONT UL, Hi-C, and Illumina PE sequencing data. With the support of advanced sequencing technologies and genome assembly algorithms and strategies, we successfully assembled 2 T2T reference genomes for sweet orange, achieving high continuity, completeness, and accuracy. These assemblies have unveiled the sequences of highly repetitive regions, including centromeres and telomeres, within the sweet orange genomes. Through comparative genomic analysis, we elucidated the evolutionary history of the sweet orange genomes, including WGD events, gene family expansions and contractions, divergence times between species, and the amplification of LTR-RTs. Collinear regions account for over 77% of the sequences between the 2 genomes, indicating significant conservation. On the other hand, extensive intraspecies variations were detected, including 2,886 SVs. We categorized these SVs and mapped their relative positions in the genome. These findings provide a valuable set of candidate genes, narrowing the scope for subsequent studies aimed at characterizing and validating genes associated with agronomic trait differences between the 2 sweet orange varieties. This study lays a high-quality data foundation for understanding the phenotypic and genetic diversity of sweet oranges.

The pangenome is a current research hotspot in plant genomics. Gap-free genomes can serve as high-quality references for constructing more comprehensive and accurate graph-based pangenomes, which, in turn, facilitate the study of polymorphisms in complex genomic regions [70]. The 2 T2T sweet orange reference genomes provided in this study enhance the existing sweet orange genomic resources. These genomes will support the construction of a graph-based pangenome for sweet orange, enable the detection and genotyping of a broader range of SVs, and, when combined with population resequencing data, help uncover the associations between sweet orange phenotypes and genotypes.

## Supplementary Material

giae084_supplement_Files

giae084_GIGA-D-24-00206_Original_Submission

giae084_GIGA-D-24-00206_Revision_1

giae084_Response_to_Reviewer_Comments_Original_Submission

giae084_Reviewer_1_Report_Original_Submission Upuli Nakandala -- 7/22/2024

giae084_Reviewer_2_Report_Original_Submission Tokurou Shimizu -- 7/23/2024

## Data Availability

The genome sequences and raw sequencing data for the Newhall and LHH navel orange genomes are available under NCBI BioProject ID PRJNA1122682. The raw sequencing data for the Newhall and LHH navel orange genomes are available from the National Genomics Data Center (NGDC) [71], under accession number PRJCA026660. The PacBio HiFi, Hi-C, ONT UL, Illumina PE, and RNA-seq sequencing data for this project can be accessed through NCBI BioProject PRJNA1122682, with corresponding numbers SRR29362821 to SRR29362830. The LHH and Newhall genomes have been deposited in DDBJ/ENA/GenBank under accession numbers JBFBVJ000000000 and JBFBVK000000000, respectively. The genomic data of Longhuihong and Newhall, as well as the other supporting data, can be found in the *GigaScience* database, GigaDB [72–74].

## Additional Files

**Supplementary Fig. S1**. BUSCO assessment results for the genomes of LHH and Newhall.

**Supplementary Fig. S2**. Statistics of integrated gene models predicted by 3 strategies. The Venn diagram depicts the quantities of gene models predicted by 3 strategies in LHH (A) and Newhall (B) genomes, along with their overlapping relationships.

**Supplementary Fig. S3**. Gene family clustering of 13 plant genomes. The Venn diagrams show unique and shared orthologous gene clusters among the 13 species (A) and the 5 citrus genomes (B).

**Supplementary Fig. S4**. Dot matrix comparison of the LHH and Newhall genomes. The x-axis represents the LHH genome coordinates, and the y-axis represents the Newhall genome coordinates.

**Supplementary Fig. S5**. (A) Composition of SVs between LHH and NHE genomes. The pie chart shows the proportion of different types of SVs across the whole genome. The stacked bar plot shows the types and quantities of SVs on each chromosome. (B) Positional distribution of SVs on each chromosome relative to the Newhall gene.

**Supplementary Table S1**. Statistics of Hi-C assembly results for the Newhall and LHH genomes.

**Supplementary Table S2**. Telomere location information for Newhall and LHH genomes.

**Supplementary Table S3**. Centromere location information for Newhall and LHH genomes.

**Supplementary Table S4**. Summary of BUSCO assessment results for LHH and Newhall genomes.

**Supplementary Table S5**. Mapping rate statistics of sequencing data for LHH and Newhall genomes.

**Supplementary Table S6**. Statistical information of TEs in LHH and Newhall genomes.

**Supplementary Table S7**. Gene model annotation results of LHH and Newhall genomes.

**Supplementary Table S8**. Summary of noncoding RNA annotation results in LHH and Newhall genomes.

**Supplementary Table S9**. Statistical summary of gene family clustering results.

**Supplementary Table S10**. SVs of homologous genes in the Newhall genome corresponding to reported cold tolerance genes in *O. sativa* Nipponbare.

## Abbreviations

BUSCO: Benchmarking Universal Single-Copy Orthologs; CCS: Circular Consensus Sequencing; CDS: coding sequence; Gb: gigabase; Hi-C: high-throughput chromosome conformation capture; HiFi: High Fidelity; KEGG: Kyoto Encyclopedia of Genes and Genomes; *Ks*: synonymous substitution rates; LAI: LTR Assembly Index; LTR: long terminal repeat; Mb: megabases; miRNA: microRNA; ML: maximum likelihood; MSA: multiple sequence alignment; Mya: million years ago; ONT UL: Oxford Nanopore Technologies Ultra Long; PAV: presence/absence variation; PE: pair end; Q-PCR: quantitative polymerase chain reaction; QV: quality value; snoRNA: small nucleolar RNA; SNP: single-nucleotide polymorphism; snRNA: small nuclear RNA; SV: structural variation; T2T: telomere-to-telomere; TE: transposable element; tRNA: transfer RNA; WGD: whole-genome duplication.
