# Supplementary material for: Construction and analysis of telomere-to-telomere genomes for 2 sweet oranges: Longhuihong and Newhall (Citrus sinensis)
Source: Gigascience. 2024 Nov 26;13:giae084. doi: 10.1093/gigascience/giae084 (PMC11590112; doi:10.1093/gigascience/giae084)
Supplement: giae084_supplement_Files [file giae084_supplement_files.zip › Supplementary Figure 1_5.pdf]

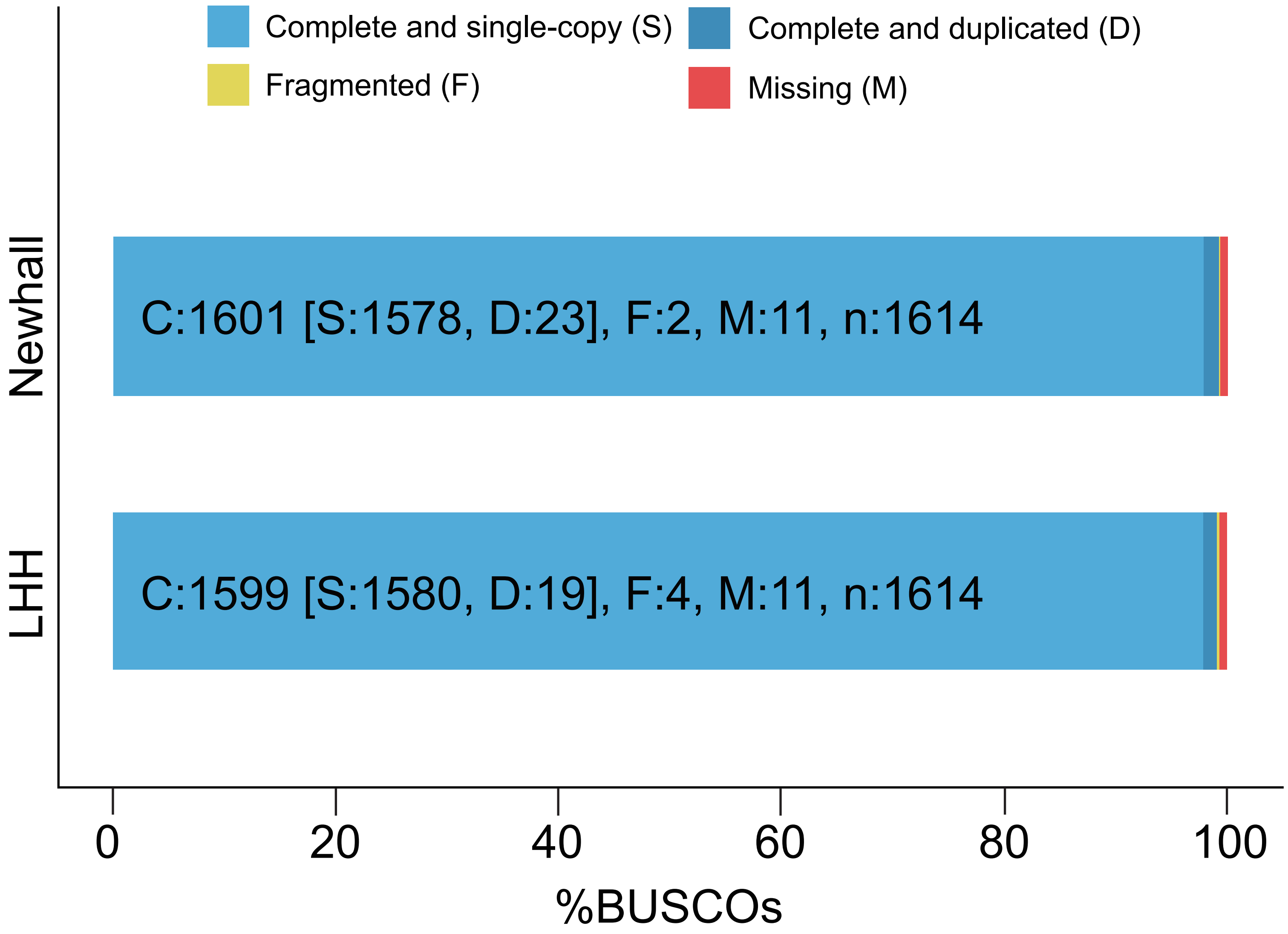

Supplementary Figure 1. BUSCO assessment results for the genomes of LHH and Newhall. The stacked bar chart shows the proportion composition of four types of BUSCOs. C: complete and single-copy BUSCO, D: complete and duplicated BUSCO, F: fragmented BUSCO, M: missing BUSCO.

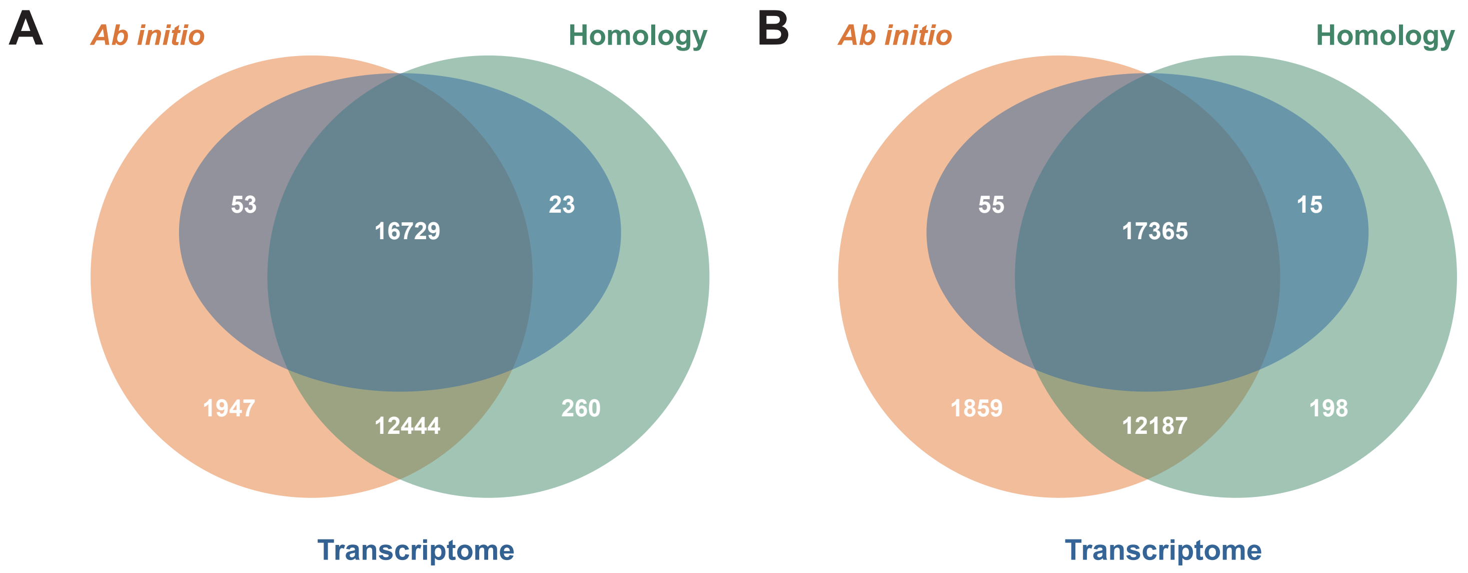

Supplementary Figure 2. Statistics of integrated gene models predicted by three strategies. The Venn diagram depicts the quantities of gene models predicted by three strategies in LHH (A) and Newhall (B) genome, along with their overlapping relationships.

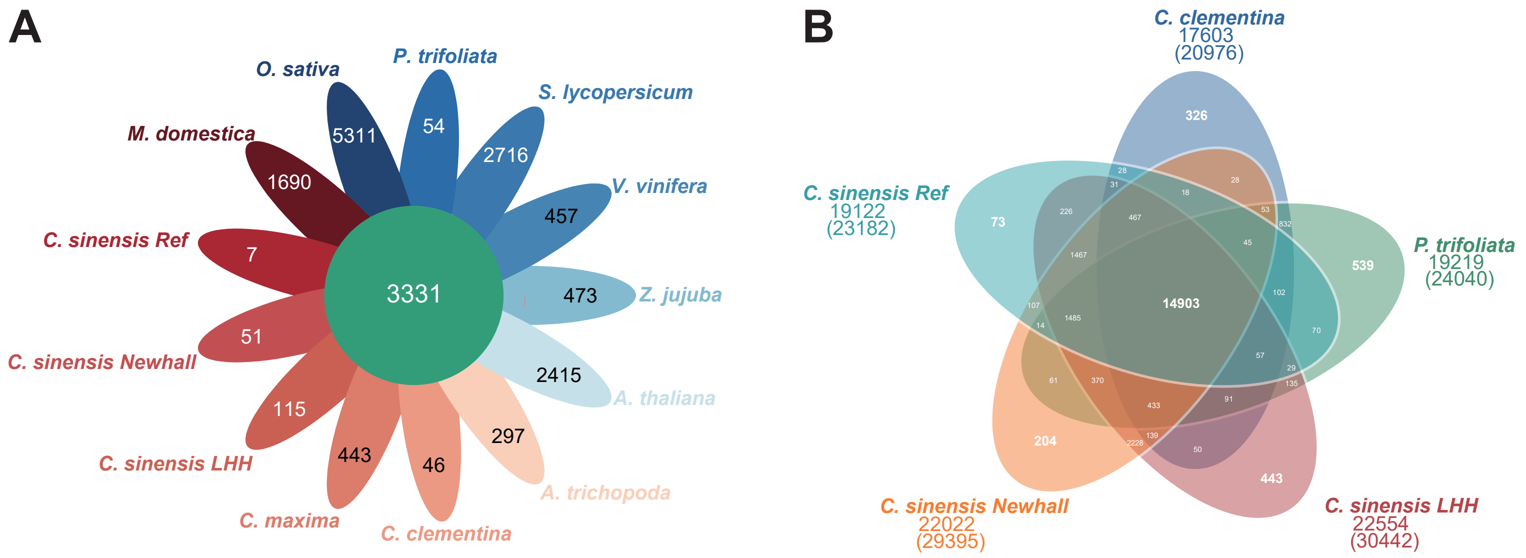

Supplementary Figure 3. Gene family clustering of 13 plant genomes. The Venn diagrams show unique and shared orthologous gene clusters among the 13 species (A) and the 5 *Citrus* genomes (B).

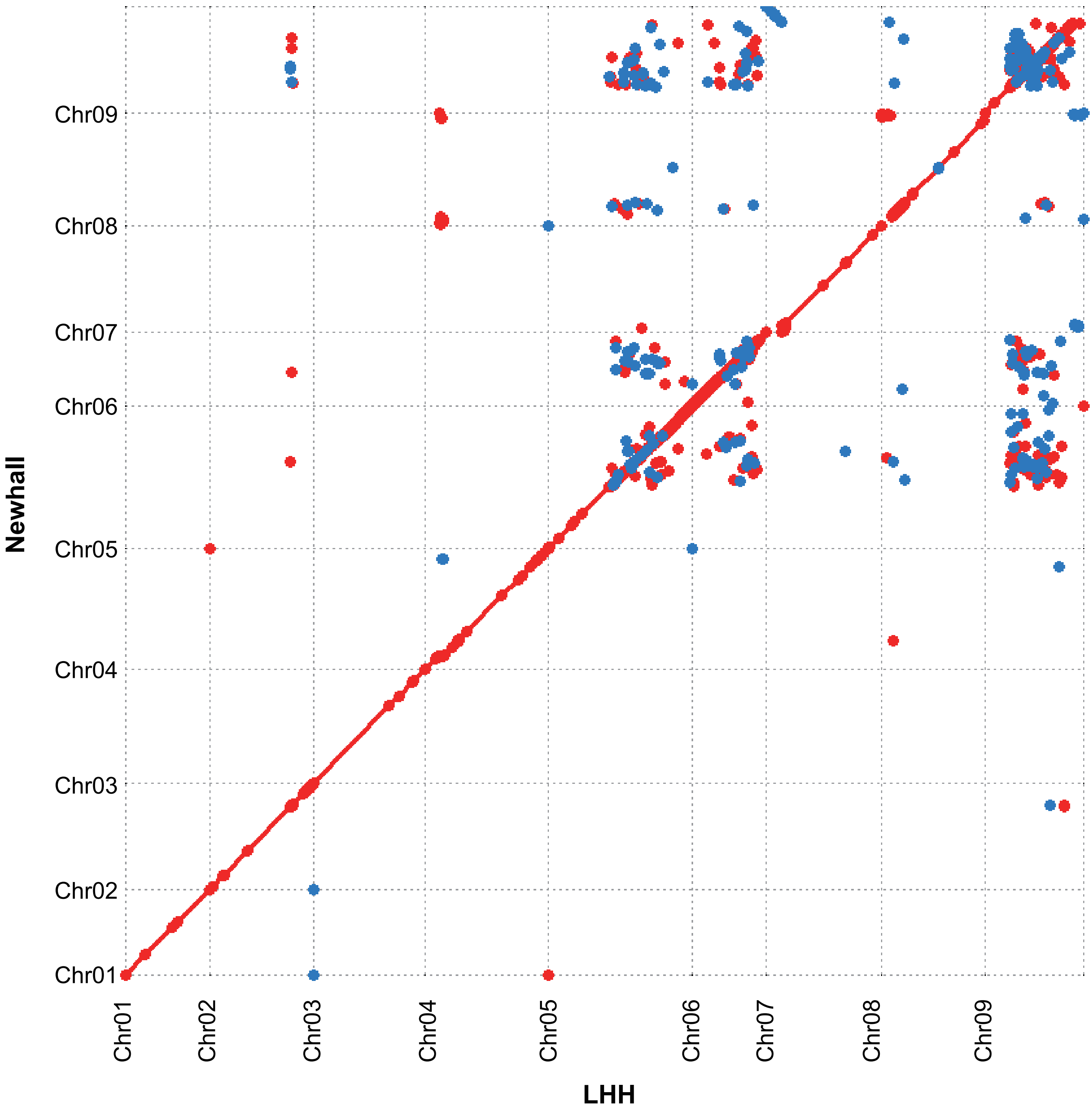

Supplementary Figure 4. Dot matrix comparison of the LHH and Newhall genomes. The x-axis represents the LHH genome coordinates, and the y-axis represents the Newhall genome coordinates.

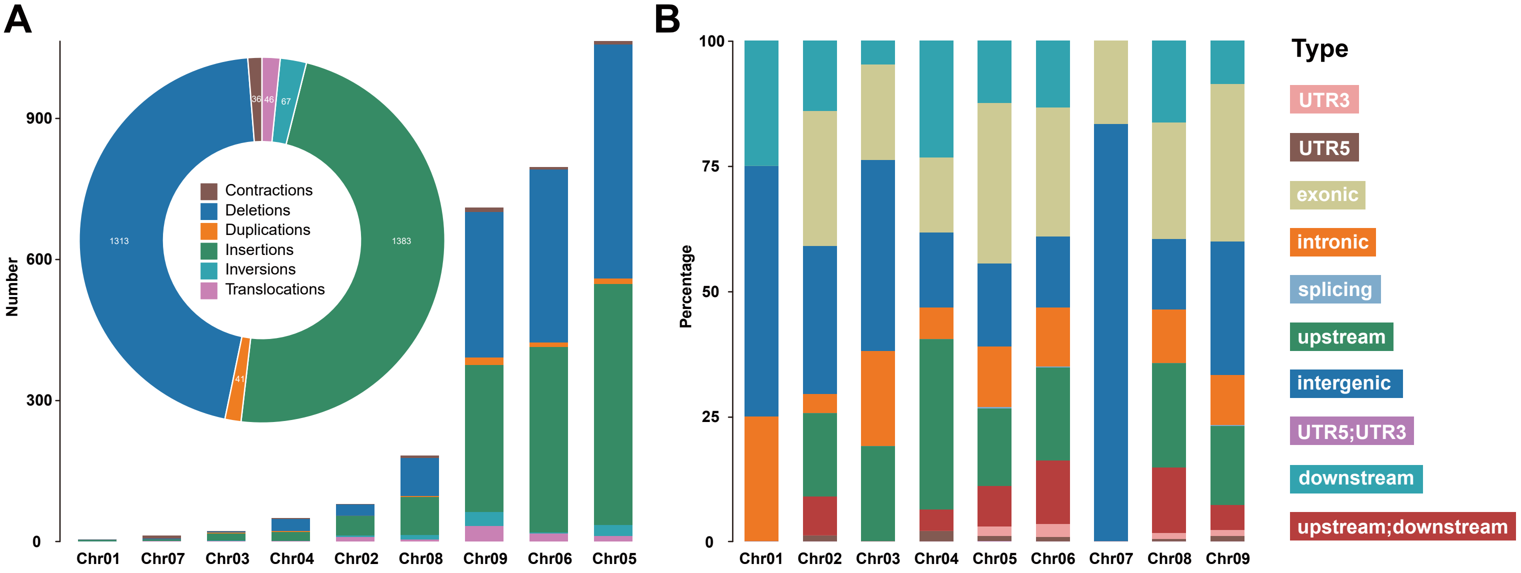

Supplementary Figure 5. (A), Composition of SVs between LHH and NHE genomes. The pie chart shows the proportion of different types of SVs across the whole genome. The stacked bar plot shows the types and quantities of SVs on each chromosome. (B), Positional distribution of SVs on each chromosome relative to the Newhall gene.
