# Supplementary material for: Construction and analysis of telomere-to-telomere genomes for 2 sweet oranges: Longhuihong and Newhall (Citrus sinensis)
Source: Gigascience. 2024 Nov 26;13:giae084. doi: 10.1093/gigascience/giae084 (PMC11590112; doi:10.1093/gigascience/giae084)
Supplement: giae084_GIGA-D-24-00206_Original_Submission [file giae084_giga-d-24-00206_original_submission.pdf]

GigaScience

Construction and analysis of telomere-to-telomere genomes for two sweet oranges: Longhuihong and Newhall (Citrus sinensis)

--Manuscript Draft--

**Manuscript Number:**	GIGA-D-24-00206
**Full Title:**	Construction and analysis of telomere-to-telomere genomes for two sweet oranges: Longhuihong and Newhall (Citrus sinensis)
**Article Type:**	Data Note
**Funding Information:**	Ministry of Agriculture and rural citrus industry cluster project and Chongqing academy of agricultural sciences municipal financial special project (NKY-2022AB005) — Dr. Lin Hong
**Abstract:**	Background<br><br>Sweet orange (Citrus sinensis Osbeck) is a fruit crop of high nutritional value that is widely consumed around the world. However, its susceptibility to low-temperature stress limits its cultivation and production in regions prone to frost damage, severely impacting the sustainable development of the sweet orange industry. Therefore, developing cold-resistant sweet orange varieties is of great necessity. Traditional hybrid breeding methods are not feasible due to the polyembryonic phenomenon in sweet oranges, necessitating the enhancement of its germplasm through molecular breeding. High-quality reference genomes are valuable for studying crop resistance to biotic and abiotic stresses. However, the lack of genomic resources for cold-resistant sweet orange varieties has hindered the progress in developing such varieties and researching their molecular mechanisms of cold resistance.<br><br>Findings<br><br>This study integrated PacBio HiFi, ONT, Hi-C, and Illumina sequencing data to assemble telomere-to-telomere (T2T) reference genomes for the cold-resistant sweet orange mutant 'Longhuihong' (Citrus sineses [L.] Osb. cv. LHH) and its wild-type counterpart 'Newhall' (Citrus sineses [L.] Osb. cv. Newhall). Comprehensive evaluations based on multiple criteria revealed that both genomes exhibit high continuity, completeness, and accuracy. The genome sizes were 340.28 Mb and 346.33 Mb, with contig N50 of 39.31 Mb and 36.77 Mb, respectively. In total, 31,456 and 30,021 gene models were annotated in the respective genomes. Leveraging these assembled genomes, comparative genomics analyses were performed, elucidating the evolutionary history of the sweet orange genome. Moreover, the study identified 2,886 structural variants (SVs) between the two genomes, with several SVs located in the upstream, downstream, or intronic regions of homologous genes known to be associated with cold resistance.<br><br>Conclusions<br><br>The study de novo assembled two T2T reference genomes of sweet orange varieties exhibiting different levels of cold tolerance. These genomes serve as valuable foundational resources for genomic research and molecular breeding aimed at enhancing cold tolerance in sweet oranges. Additionally, they expand the existing repository of reference genomes and sequencing data resources for Citrus sinensis. Moreover, these genomes provide a critical data foundation for comparative genomics analyses across different plant species.
**Corresponding Author:**	Jia-Ming Song<br>Southwest University<br>Chongqing, CHINA
**Corresponding Author Secondary Information:**	
**Corresponding Author's Institution:**	Southwest University

Powered by Editorial Manager® and ProduXion Manager® from Aries Systems Corporation

Corresponding Author's Secondary Institution:	
First Author:	Lin Hong
First Author Secondary Information:	
Order of Authors:	Lin Hong
	Xin-Dong Xu
	Lei Yang
	Min Wang
	Shuang Li
	Haijian Yang
	Si-Ying Ye
	Ling-Ling Chen
	Jia-Ming Song
Order of Authors Secondary Information:	
Additional Information:	
**Question**	**Response**
Are you submitting this manuscript to a special series or article collection?	No
**Experimental design and statistics**<br><br>Full details of the experimental design and statistical methods used should be given in the Methods section, as detailed in our Minimum Standards Reporting Checklist. Information essential to interpreting the data presented should be made available in the figure legends.<br><br>Have you included all the information requested in your manuscript?	Yes
**Resources**<br><br>A description of all resources used, including antibodies, cell lines, animals and software tools, with enough information to allow them to be uniquely identified, should be included in the Methods section. Authors are strongly encouraged to cite Research Resource Identifiers (RRIDs) for antibodies, model organisms and tools, where possible.	Yes

Powered by Editorial Manager® and ProduXion Manager® from Aries Systems Corporation

Have you included the information requested as detailed in our Minimum Standards Reporting Checklist?	
**Availability of data and materials**<br><br>All datasets and code on which the conclusions of the paper rely must be either included in your submission or deposited in publicly available repositories (where available and ethically appropriate), referencing such data using a unique identifier in the references and in the "Availability of Data and Materials" section of your manuscript.<br><br>Have you have met the above requirement as detailed in our Minimum Standards Reporting Checklist?	Yes

Powered by Editorial Manager® and ProduXion Manager® from Aries Systems Corporation

Manuscript

Click here to access/download;Manuscript;0612-Orange_GigaScience_DataNote_20240612_Manuscript_Clean

Construction and analysis of telomere-to-telomere genomes for two sweet oranges: Longhuihong and Newhall (*Citrus sinensis*)

Lin Hong[1,#,*], Xin-Dong Xu[2,3,#], Lei Yang[1,#], Min Wang[1], Shuang Li[1], Haijian Yang[1], Si-Ying Ye[2,3], Ling-Ling Chen[3], Jia-Ming Song[2,3]*, Lin Hong[1,#,*]

[1]Fruit Tree Research Institute, Chongqing Academy of Agricultural Sciences, Chongqing 401329, China

[2]Integrative Science Center of Germplasm Creation in Western China (CHONGQING) Science City and Southwest University, College of Agronomy and Biotechnology, Southwest University, Chongqing 400715, China

[3]State Key Laboratory for Conservation and Utilization of Subtropical Agro-bioresources, College of Life Science and Technology, Guangxi University, Nanning 530004, China

E-mail:

loquatvalue@163.com (L.H.);

1546509060@qq.com (X.-D.X.);

leir8512@126.com (L.Y.);

wm950918@126.com (M.W.);

sclishuang61@163.com (S.L.);

yanghaijian126@126.com (H.Y.);

siyingyesdfmu@163.com (S.-Y.Y.);

llchen@gxu.edu.cn (L.-L.C.);

jmsong@swu.edu.cn (J.-M.S.);

loquatvalue@163.com (L.H.)

[#]These authors contributed equally: Lin Hong, Xin-Dong Xu, Lei Yang

*Correspondence: Lin Hong (loquatvalue@163.com), Jia-Ming Song (jmsong@swu.edu.cn)

Abstract

Background: Sweet orange (*Citrus sinensis* Osbeck) is a fruit crop of high nutritional value that is widely consumed around the world. However, its susceptibility to low-temperature stress limits its cultivation and production in regions prone to frost damage, severely impacting the sustainable development of the sweet orange industry. Therefore, developing cold-resistant sweet orange varieties is of great necessity. Traditional hybrid breeding methods are not feasible due to the polyembryonic phenomenon in sweet oranges, necessitating the enhancement of its germplasm through molecular breeding. High-quality reference genomes are valuable for studying crop resistance to biotic and abiotic stresses. However, the lack of genomic resources for cold-resistant sweet orange varieties has hindered the progress in developing such varieties and researching their molecular mechanisms of cold resistance.

Findings: This study integrated PacBio HiFi, ONT, Hi-C, and Illumina sequencing data to assemble telomere-to-telomere (T2T) reference genomes for the cold-resistant sweet orange mutant ‘Longhuihong’ (*Citrus sineses* [L.] Osb. cv. LHH) and its wild-type counterpart ‘Newhall’ (*Citrus sineses* [L.] Osb. cv. Newhall). Comprehensive evaluations based on multiple criteria revealed that both genomes exhibit high continuity, completeness, and accuracy. The genome sizes were 340.28 Mb and 346.33 Mb, with contig N50 of 39.31 Mb and 36.77 Mb, respectively. In total, 31,456 and 30,021 gene models were annotated in the respective genomes. Leveraging these assembled genomes, comparative genomics analyses were performed, elucidating the evolutionary history of the sweet orange genome. Moreover, the study identified 2,886 structural variants (SVs) between the two genomes, with several SVs located in the upstream, downstream, or intronic regions of homologous genes known to be associated with cold resistance.

Conclusions: The study *de novo* assembled two T2T reference genomes of sweet orange varieties exhibiting different levels of cold tolerance. These genomes serve as valuable foundational resources for genomic research and molecular breeding aimed at enhancing cold tolerance in sweet oranges. Additionally, they expand the existing repository of reference genomes and sequencing data resources for *Citrus sinensis*. Moreover, these genomes provide a critical data foundation for comparative genomics analyses across different plant species.

Keywords: sweet orange; *Citrus sineses*; Longhuihong; Newhall; telomere-to-telomere genome; cold tolerance

Data Description

Context

The sweet orange (*Citrus sinensis* Osbeck), a member of the Rutaceae family and Citrus genus, is globally recognized as one of the most commercially valuable fruits due to its high content of bioactive compounds such as flavonoids, phenolic acids, alkaloids, carotenoids, and limonoids, which provide significant anti-inflammatory, anti-cancer, and antioxidant benefits. These properties make sweet oranges widely applicable in agriculture, food, and medicine [1,2]. Sweet oranges can be consumed fresh or juiced and are generally categorized into three types: blond oranges, navel oranges, and blood oranges [3]. Navel oranges, distinguished by a secondary fruitlet (resembling a navel) at the bottom of the fruit, have a high mutation rate that has facilitated their proliferation, with numerous cultivars being bred and disseminated worldwide [4]. To date, there are over 190 known varieties, with commonly cultivated types including ‘Washington’, ‘Newhall’, ‘Chislett’, ‘Powell’, ‘Lane Late’, ‘Barnfield’, and ‘Cara Cara’ [4,5]. Somatic mutation-based bud sport breeding has become a vital method for

developing new fruit tree varieties. It is estimated that around 80% of sweet orange cultivars originate from somatic mutations, with sweet oranges being cultivated in 114 countries globally. Moreover, the apomictic nature of sweet oranges allows for strict clonal propagation under natural conditions, making them an ideal species for studying somatic mutations [6,7].

The genome is considered a foundational element in biological research. In 2000, the first plant genome, the *Arabidopsis thaliana* genome, was published by scientists [8]. Over the past two decades, with rapid advancements in sequencing technology, assembly algorithms, genetics, and bioinformatics [9], nearly 900 plant species' genomes have been published to date (based on data from NCBI and GWH). In 2012, the first genome of the sweet orange was sequenced. To reduce assembly complexity, double haploid sequencing was utilized by researchers, resulting in the successful mapping of 87% of the sweet orange genome. The total assembly size was determined to be 320 Mb, with a contig N50 of 49.89 Kb, a scaffold N50 of 1.69 Mb, and 29,445 protein-coding genes were identified [10]. In 2014, the genome sequences of seven citrus species, including clementine, mandarin, pummelo, sweet orange, and sour orange, were completed. Higher-quality genomes for the Citrus genus were produced, significantly improving upon the previously published sweet orange genome (contig N50 = 119 Kb, scaffold N50 = 6.8 Mb). Evolution and domestication of the Citrus genus were explored, indicating that the cultivated pummelo originated from the ancestral species *C. maxima*, while the cultivated mandarin was derived from *C. reticulata* with introgressions from *C. maxima* [11]. In 2017, single-molecule sequencing technology was employed to complete the genomes of four representative citrus species, achieving a contig N50 of 2.2 Mb, which is over 18 times greater than previously reported citrus genomes. Comparative genomic analysis revealed that the citrus-specific genomic regions primarily consist of repeat sequences and genes of unknown function, with approximately one-fifth of

the genes having known functions and being enriched in biological pathways related to resistance, proteolysis, and pectin degradation [12]. In 2021, a bud sports population was combined with genomics strategies to improve the scope and accuracy of somatic mutation predictions. The genomes of a double haploid sweet orange and six diploids were first assembled, leaving an average of only three gaps per chromosome across the nine chromosomes [7]. In 2023, a genomic map of the Citrus subfamily was constructed, with de novo assembly of the genomes of 12 Citrus genus and closely related species. The Contig N50 of these genomes ranged from 1.6 to 16.8 Mb, with assembly sizes ranging from 217.8 to 419.1 Mb, thus covering over 90% of their estimated genome sizes. The number of annotated gene models in these genomes ranged from 22,907 to 31,413 [13]. In 2023, a complete and haplotype-resolved T2T genome of the lemon variety perfume lemon was assembled by researchers. The assembled genome is 633.0 Mb in size, with a contig N50 of 35.6 Mb and zero gaps, achieving a fully gap-free assembly. Further multi-omics analyses identified candidate genes associated with the biosynthesis of flavor compounds and resistance to Huanglongbing (HLB), providing a foundation for accelerating molecular breeding programs and the discovery of functional genes [14]. In 2024, a study has reported the T2T genome of the ‘Nei Xiu’, a bud mutation originating from Tarocco blood orange[15].

‘Longhuihong’ (LHH) is a bud mutation of the ‘Newhall’ navel orange, known for its enhanced cold tolerance. The low-temperature stress during winter stimulates the synthesis of anthocyanins in LHH fruits, leading to visibly red juice sacs (Fig. 1A). The leaves display distinct curling and prominent veins, making its variation characteristics highly noticeable (Fig. 1B). Variety trials have demonstrated that LHH possesses several agronomic traits superior to those of Newhall, indicating that it may harbor multiple advantageous genotypes with significant development potential. However,

the lack of available genomic resources for LHH currently limits the exploration and utilization of the genetic basis behind its desirable traits—such as cold tolerance, high photosynthetic efficiency, and high anthocyanin content. This poses a significant challenge to molecular breeding efforts for sweet oranges.

This study leveraged state-of-the-art sequencing technologies, including PacBio High Fidelity (HiFi), Oxford Nanopore Technologies Ultra Long (ONT UL), high-throughput chromosome conformation capture (Hi-C), and Illumina sequencing data, to report the T2T genome assembly of navel orange varieties, LHH and Newhall. These assemblies provide valuable genomic resources for pangenome studies, functional gene identification, and molecular breeding of sweet oranges. Furthermore, by utilizing the two T2T genomes, we elucidated the evolutionary history of the sweet orange genome and characterized the genomic variations between the two varieties. This research offers critical data support for future genomic studies and breeding programs aimed at enhancing cold tolerance in sweet oranges.

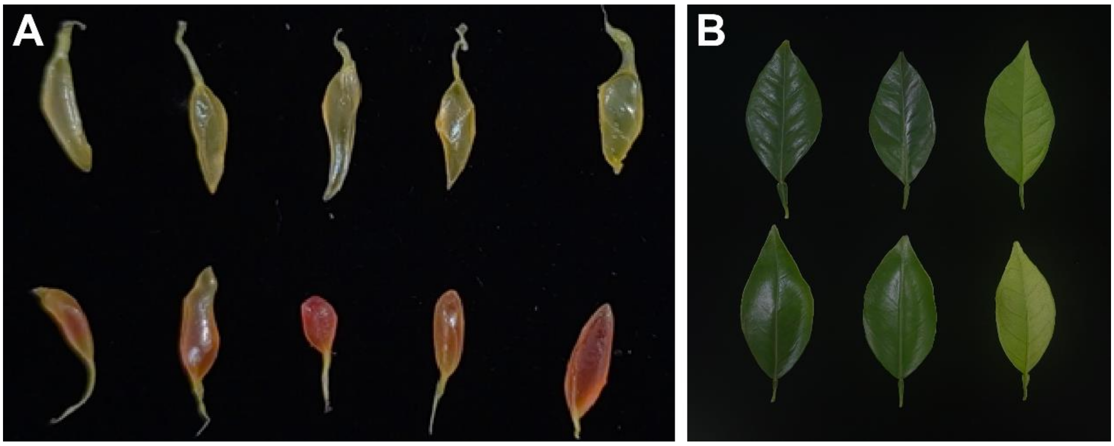

Figure 1: Morphological characteristics of *C. sineses* LHH and Newhall. (A), Comparison of juice sac characteristics between navel orange cultivar Newhall (top) and LHH (bottom). (B), Comparison of leaf characteristics between navel orange cultivar ‘LHH’ (top) and ‘Newhall’ (bottom).

Sample collection

Samples of the navel orange varieties LHH and Newhall were collected from a mid-to-high altitude experimental orchard specializing in sweet orange varieties in Fengming Town, Yunyang County, Chongqing, at an elevation of 750 meters. Fresh leaf samples were obtained from 4-year-old trees that had been grafted onto trifoliate orange rootstocks and planted at a density of 5 meters by 3 meters. These samples were then used for subsequent DNA extraction, library construction, and sequencing.

DNA isolation, library construction, and sequencing

The experimental procedures for DNA extraction used in Illumina sequencing were performed according to the standard protocol provided by Illumina. Qualified genomic DNA samples were then used for the construction of sequencing libraries. The size and quantity of library fragments were assessed using Qseq400 and Qubit, respectively, ensuring library quality. The qualified libraries were subsequently immobilized onto sequencing chips via bridge PCR methods. Illumina sequencing was eventually conducted, performing 150 bp paired-end sequencing on an Illumina sequencer. For PacBio Circular Consensus Sequencing (CCS), long-fragment library construction was carried out using genomic DNA extracted from leaf samples, adhering to the manufacturer's protocol. The genomic DNA was then sheared into 15 kb fragments. Sequencing of the constructed library was executed on the PacBio Sequel II platform. Post-sequencing, low-quality reads and sequencing adapters were removed to obtain clean subreads. Regarding ONT UL sequencing, library preparation was conducted using the SQK-ULK001 kit, following the standard protocol. Libraries were purified and sequenced

using a PromethION sequencer. Fresh leaf tissue samples from the two sweet orange varieties were utilized for Hi-C library construction. The concentration and insert size of the constructed libraries were evaluated using Qubit 2.0 and Agilent 2100, respectively, while quantitative PCR (Q-PCR) was used to determine the effective concentration of the Hi-C libraries. Upon passing quality checks, Illumina-based high-throughput sequencing was performed, generating 150 bp paired-end reads.

Ultimately, for the LHH and Newhall varieties, 28.04 Gb (~71×) and 26.07 Gb (~79×) of PacBio HiFi reads, 20.58 Gb (~51×) and 24.49 Gb (~61×) of ONT UL reads, 18.25 Gb (~43×) and 17.18 Gb (~46×) of Illumina PE reads, as well as 50.69 Gb (~136×) and 50.43 Gb (~146×) of Hi-C reads were obtained, respectively (Table 1).

Table 1: Statistics of the clean data of the *C. sinensis* LHH and Newhall genomes

	C. sinensis LHH			*C. sinensis* Newhall		
Type	Total data (Gb)	Sequence depth (×)	Average size (bp)	Total data (Gb)	Sequence depth (×)	Average size (bp)
PacBio	28.04	71	13,757	26.07	79	13,965
ONT	20.58	51	98,866	24.49	61	95,293
Hi-C	50.69	136	150	50.43	146	150
Illumina	18.25	43	150	17.18	46	150

Genome survey

Before genome assembly, a 21-mer-based survey was conducted on the LHH and Newhall genomes to estimate genome size, heterozygosity, repeat content, and ploidy level, providing critical reference information for subsequent assembly efforts. Jellyfish (v2.3.1) [16] was employed to count the 21-mers from the Illumina paired-end sequencing data. Following this, GenomeScope (v2.0) [17] was used to analyze the 21-mer frequency distributions. Smudgeplot (v0.2.5) [17] was utilized to

estimate genome ploidy. The survey results revealed that the estimated genome sizes for LHH and Newhall were 310.75 Mb and 301.9 Mb, respectively, with heterozygosities of 2.79% and 2.67%. Both genomes exhibited high levels of heterozygosity and similar repeat content, 45.0% and 44.8%, respectively (Fig. 1A, C). Ploidy estimation results indicated that both LHH and Newhall genomes are heterozygous diploids, consistent with previously reported sweet orange genomes (Fig. 1B, D).

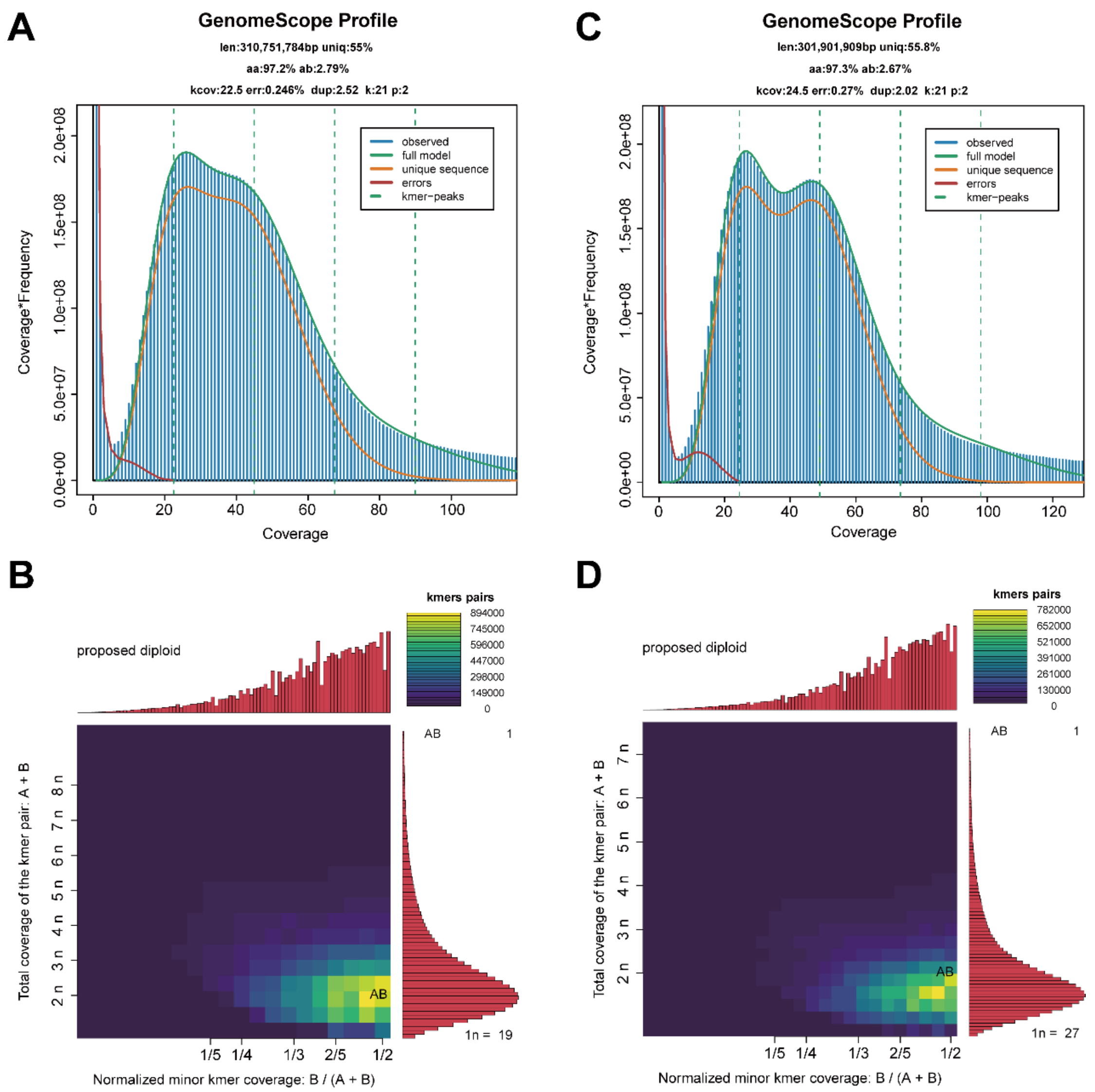

Figure 2: The genomic feature survey of *C. sinensis* LHH and Newhall genomes based on 21-mer. (A), 21-mer spectra for *C. sineses* LHH. (B), Smudgeplots for *C. sineses* LHH. (C), 21-mer spectra for *C. sineses* Newhall. (D), Smudgeplots for *C. sineses* Newhall.

Genome assembly and assessment

Based on the PacBio HiFi, ONT UL, and Hi-C sequencing data obtained from whole-genome sequencing, the LHH and Newhall navel orange genomes were de novo assembled. Hifiasm (v0.19) [18] was utilized for the initial assembly of the LHH and Newhall genomes, with ONT UL sequencing data integrated using the "–ul" parameter to enhance the assembly process. Initial assembly results indicated that the LHH and Newhall genomes contained 160 and 141 contigs, respectively, with total lengths of 372.71 Mb and 370.23 Mb, and contig N50 values of 36.91 Mb and 36.77 Mb. Subsequently, the initially assembled contigs were aligned to the NCBI NT database, the mitochondrial database (https://ftp.ncbi.nlm.nih.gov/refseq/release/mitochondrion/), and the plastid database (https://ftp.ncbi.nlm.nih.gov/refseq/release/plastid/) to filter out contaminants, mitochondrial sequences, and plastid sequences, resulting in clean contig sequences. Leveraging Hi-C contact signals, LACHESIS [19] was employed to group, order, and orient the contigs with the parameters: "CLUSTER_MIN_RE_SITES = 100; CLUSTER_MAX_LINK_DENSITY = 2; ORDER_MIN_N_RES_IN_TRUNK = 15; ORDER_MIN_N_RES_IN_SHREDS = 15." Subsequently, Juicebox [20] was used to manually inspect and adjust the scaffolding results from LACHESIS, achieving chromosome-level genomes for both LHH and Newhall sweet orange (Fig. 2C, D). The statistics revealed that 338.55 Mb and 337.47 Mb of the clean contigs for LHH and Newhall, respectively, were anchored to 9 pseudochromosomes, with anchoring rates of 99.49% and 97.44% (Table S1). TGS-Gapcloser (v1.2.1) [21] was utilized to close gaps in the anchored reference genome using ONT UL sequencing data. Ultimately, 8 and 7 gap-free chromosome assemblies were obtained

for the LHH and Newhall sweet orange varieties, respectively. Potential telomeric repeat units within the genome were identified using TIDK (https://github.com/tolkit/telomeric-identifier). Subsequently, potential telomeric sequences were located with FindTelomeres (https://github.com/JanaSperschneider/FindTelomeres) based on these repeat units, enabling the acquisition of both telomeric positions and sequences. The identification of potential centromeric repeats was accomplished using the Centromics tool (https://github.com/ShuaiNIEgithub/Centromics) with third-generation sequencing reads, which were then mapped back to the genome to determine centromeric positions and sequences. Telomeric sequences were detected at both ends of 7 chromosomes in each genome, while the remaining chromosomes exhibited telomeric sequences at one end. Centromeric sequences were identified on all chromosomes in both genomes, indicating that both assemblies achieved a T2T level [22] (Fig. 3E, F, Table S2, 3). The total lengths of the T2T genomes for LHH and Newhall were 340.28 Mb and 346.33 Mb, respectively, consistent with the genome sizes estimated from the genome surveys.

After completing the assembly of the two sweet orange genomes, various strategies were employed to verify their completeness and accuracy. The Benchmarking Universal Single-Copy Orthologs (BUSCO) [23] dataset, embryophyta_odb10, comprising 1614 genes, was mapped to the genomes, yielding BUSCO completeness scores of 99.07% for LHH and 99.19% for Newhall (Fig. S1, Table S4). Additionally, the Core Eukaryotic Genes Mapping Approach (CEGMA) [24] was utilized for further evaluation, indicating completeness scores of 99.13% for the LHH genome and 99.56% for the Newhall genome. Minimap2 (v2.26-r1175) [25] was then used to map the HiFi and ONT UL reads to the genomes. The mapping statistics showed that the HiFi reads had mapping rates of 99.11% for LHH and 99.58% for Newhall, with coverages of 99.98% and 99.99%, and average read depths of 71×

and 68×, respectively. For the ONT UL reads, the mapping rates were 96.87% for LHH and 97.05% for Newhall, with coverages of 99.96% and 99.77%, and average read depths of 51× and 61×, respectively (Table S5). These results underscore the high completeness of both genome assemblies. The Hi-C contact matrices for both genomes displayed smooth and continuous Hi-C signals, confirming the correct order and orientation of the genome assemblies (Fig. 3C, D). LTR_retriever (v2.9.8) [26] was employed to calculate the LTR Assembly Index (LAI) based on Long Terminal Repeat (LTR) annotations, to evaluate the assembly quality of repetitive sequences in both genomes [27]. The results showed an LAI of 20.39 for LHH and 20.09 for Newhall, meeting the gold reference genome standard [27]. Further analysis of LAI across different chromosomal regions indicated that most regions of the T2T genomes exhibited high LAI, and the mapping results for HiFi and ONT UL reads demonstrated uniform coverage in these areas (Fig. 3E, F). However, some regions showed relatively lower LAI, likely due to lower HiFi read coverage in those areas. Nevertheless, ONT UL reads successfully covered and filled these regions (Fig. 3E, F). Finally, Merqury (v1.3) [28] was utilized to calculate the quality values (QV) for the two genomes, yielding QV scores of 46.64 for LHH and 38.89 for Newhall, indicating high genome accuracy.

Table 2: Assembly statistics of the *C. sinensis* LHH and Newhall genomes

	C. sinensis LHH		*C. sinensis* Newhall	
Items	Contig	Scaffold	Contig	Scaffold
Sequence Number	24	9	141	9
Assembly Size (Mb)	361.41	338.51	370.23	334.63
Longest SeqLen (Mb)	50.84	50.84	49.40	49.40
Average SeqLen (Mb)	15.06	37.61	2.62	37.18
N50 (Mb)	36.91	39.31	36.77	38.85

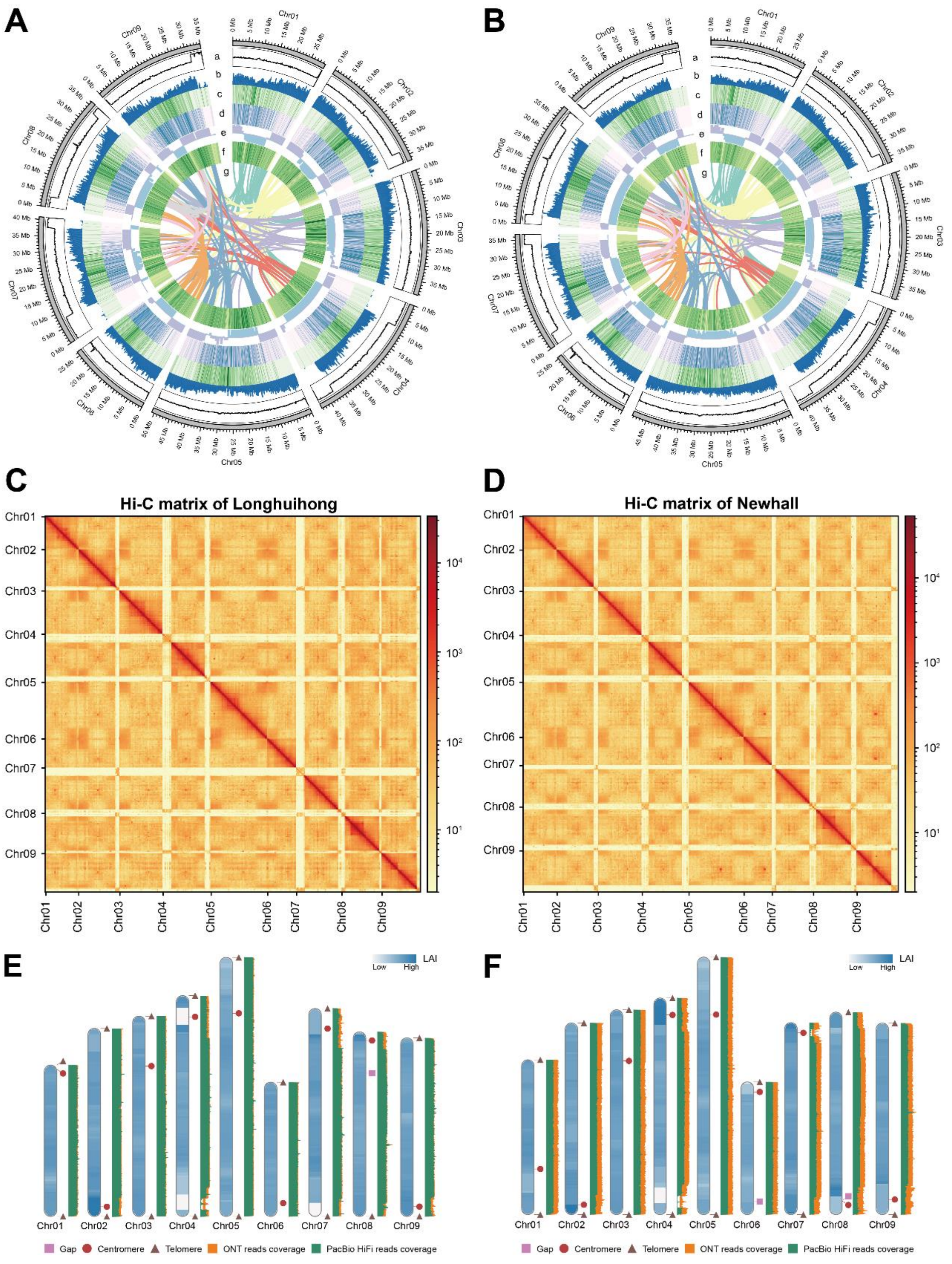

Figure 3: Assembly and assessment of the *C. sinensis* LHH and Newhall genomes. (A-B), Circos plot of the genomic landscape of the *C. sinensis* LHH genome (A) and *C. sinensis* Newhall genome (B). The circos plots show, outermost to innermost, GC content (a), gene density (b), LTR/*Gypsy* density,

LTR/*Copia* density (d), A/B compartment (e), DNA transposon density (f), and syntenic regions within the genome (g). (C-D), Hi-C contact matrix of LHH genome (C) and Newhall genome (D). (E-F), The distribution landscape of centromeres, telomeres, LAI, HiFi reads coverage, and ONT reads coverage in the LHH genome (E) and Newhall genome (F).

Genome annotation

The study initially performed repeat annotation on both genomes and masked the repetitive regions to improve the efficiency and accuracy of genome structural annotation. *De novo* prediction of repetitive sequences was performed using RepeatModeler (v2.0.1) [29], while LTR_retriever (v2.9.0) [26] was utilized for de novo prediction of LTRs. The results from both tools were merged with Repbase [30] and deduplicated to construct a comprehensive repeat library for the two navel orange genomes. This repeat library was then used by RepeatMasker (v4.1.2) [31] for genome-wide repeat annotation, resulting in masked versions of the genomes. The results indicated that 205,650 and 197,501 transposable elements (TEs) were identified in the LHH and Newhall genomes, with total lengths of 115.70 Mb and 116.79 Mb, respectively, accounting for 34.00% and 33.72% of each genome (Table S6). Among these, LTR/Gypsy elements were the most abundant, comprising 11.37% and 12.69% of the LHH and Newhall genomes, respectively (Table S6). Furthermore, class I transposons, or retrotransposons, were found to be more prevalent than class II transposons, or DNA transposons, in both genomes, consistent with patterns observed in other plant species [32]. HiCExplorer (v3.7.4) [33] was used to analyze A/B compartments from Hi-C data. A comparison with the distribution of TEs revealed a high density of TEs in the central regions of the chromosomes, corresponding to the B compartment areas, which are characterized by low gene density, low transcriptional activity, and high

chromatin condensation (Fig. 3A, B).

Subsequently, a comprehensive annotation strategy was implemented in the study, integrating Ab initio, homology-based, and transcript evidence-based approaches to annotate gene structures in the repeat-masked LHH and Newhall genomes. The analysis identified 31,456 and 30,021 gene models in the LHH and Newhall genomes, respectively (Fig. S2, Table S7). Further statistical analysis of these gene models revealed that the distribution of annotated genes, coding sequences (CDS), exons, and intron lengths in both genomes were comparable to those reported in previously described sweet orange and closely related species genomes. This consistency underscores the accuracy and reliability of the annotated gene models (Fig. 4).

Additionally, the NR, eggNOG [34], GO, KEGG [35], TrEMBL [36], KOG, SWISS-PROT [36] and Pfam [37] databases were employed to perform functional annotation of these gene sequences. As a result, 96.66% and 97.6% of the genes in the LHH and Newhall genomes, respectively, were successfully annotated using these resources. Moreover, the study focused on identifying non-coding RNAs in both genomes. tRNAscan-SE (v1.3.1) [38] was utilized for the recognition of transfer RNA (tRNA), and Barrnap (v0.9) (https://github.com/tseemann/barrnap) was used for the prediction of ribosomal RNA (rRNA). MicroRNAs (miRNAs), small nucleolar RNAs (snoRNAs), and small nuclear RNAs (snRNAs) were identified based on the Rfam (v14.5) [39] database using Infernal (v1.1) [40] The analysis revealed that 411 and 418 tRNAs, 714 and 3,920 rRNAs, and 167 and 166 miRNAs were annotated in the LHH and Newhall genomes, respectively (Table S8).

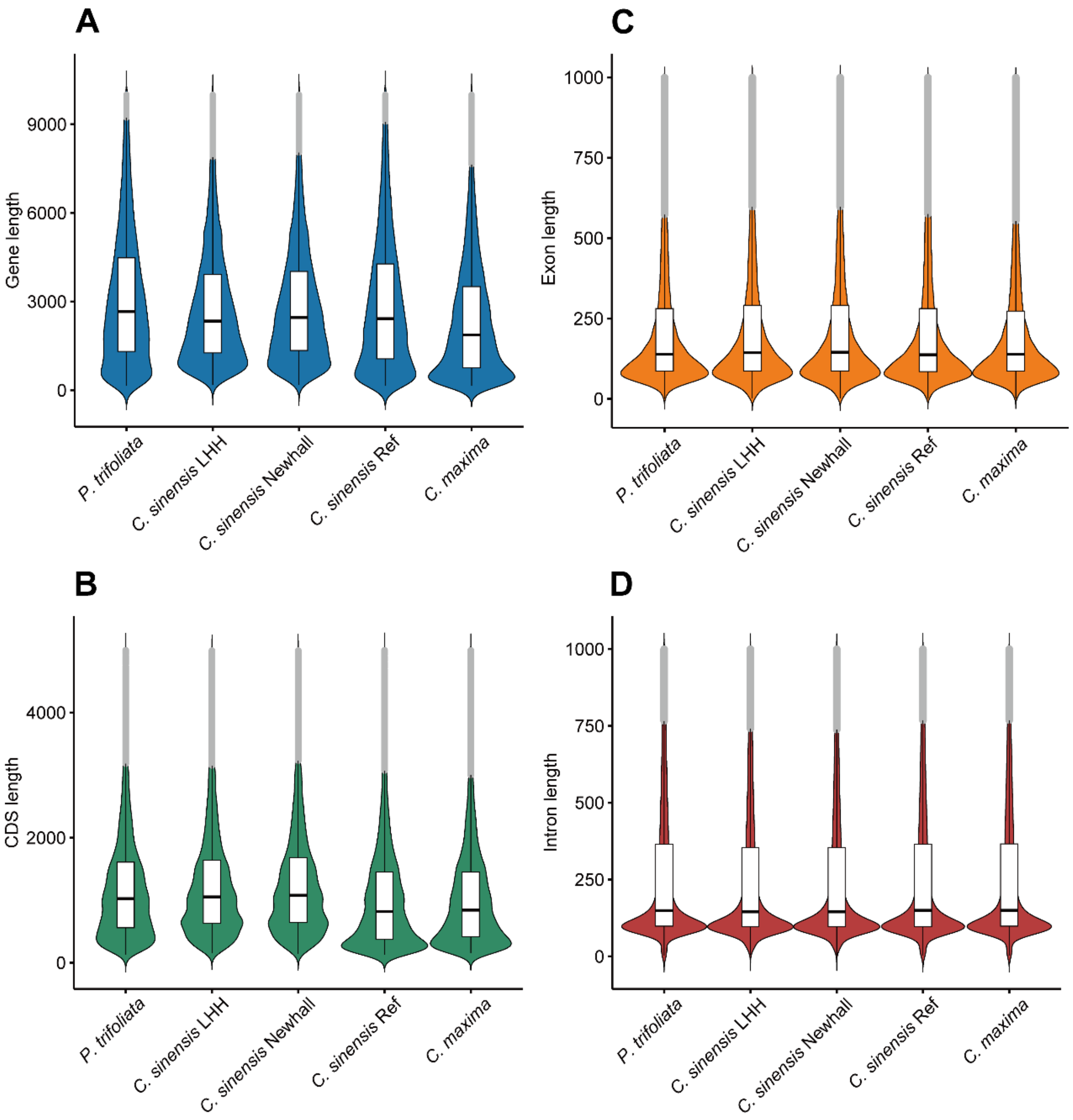

Figure 4: Statistical analysis and assessment of the annotation results for the *C. sinensis* LHH and Newhall genome. Comparison of gene length (A), CDS length (B), exon length (C) and intron length (D) distribution between the genomes of LHH, Newhall and closely related species.

Table 3: Statistics of function annotation results for *C. sinensis* LHH and Newhall

	C. sinensis LHH		*C. sinensis* Newhall	
Database	Annotated number	Annotated ratio (%)	Annotated number	Annotated ratio (%)
GO	24,741	78.65	23,881	79.55
KEGG	23,558	74.89	22,846	76.1

KOG	16,155	51.36	15,641	52.1
Pfam	25,420	80.81	24,722	82.35
SWISS-PROT	21,482	68.29	20,922	69.69
TrEMBL	30,266	96.22	29,198	97.26
eggNOG	25,306	80.45	24,461	81.48
NR	30,106	95.71	29,078	96.86
Total	30,404	96.66	29,299	97.60

Comparative genomics and evolutionary analysis

Based on the T2T genomes of LHH and Newhall, the study aimed to elucidate the evolutionary history of the navel orange genome. OrthoFinder (v2.4) [41] was utilized to cluster a total of 392,184 genes from 13 species, including *C. sinensis* LHH and Newhall, into 43,324 gene families. Among these, 3,331 gene families were found to be common to all 13 species. Specifically, 30,442 and 29,395 genes from LHH and Newhall were clustered into 22,554 and 22,022 gene families, respectively, with 282 and 122 gene families being unique to each (Fig. S3A, Table S9). Analysis of five species within the Citrus genus revealed that 13,531 gene families were common across all five species, while LHH and Newhall had 282 and 122 unique gene families, respectively (Fig. S3B). A review of gene copy numbers indicated that the proportion of genes with varying copy numbers in the LHH and Newhall genomes was comparable, with single-copy genes being the most prevalent (Fig. 5A).

Through gene family clustering analysis, 926 single-copy orthologs were identified across the 13 species. MAFFT (v7.205) [42] was used to generate multiple sequence alignments (MSA) for these single-copy orthologs, and Gblocks (v0.91b) [43] was employed to remove highly variable regions from the MSA. The MSA of single-copy orthologs was then concatenated. IQ-TREE (v1.6.11) [44] was used to construct a maximum likelihood (ML) phylogenetic tree based on the MSA, applying the

model “JTT+F+I+G4” and a bootstrap value of 1000. The results indicated that the five Citrus species, along with the closely related species P. trifoliata, clustered within the same branch, demonstrating high accuracy and reliability of the phylogenetic tree. Furthermore, using PAML (v4.9i) [45] and the mcmctree module, we estimated that the divergence time between P. trifoliata and the five Citrus species occurred approximately 13.07 million years ago (Mya), during the Miocene epoch (Fig. 5A).

CAFE (v4.2) [46] was used to analyze gene family expansions and contractions in each species. The results revealed 907 and 770 expanded gene families in LHH and Newhall, respectively, as well as 673 contracted gene families in LHH and 1,141 in Newhall (Fig. 5A). The distribution of synonymous substitution rates (*Ks*) was used to analyze whole-genome duplication (WGD) events in the navel orange genomes. The LHH and Newhall genomes were found to have undergone two rounds of WGD, one ancient WGD event (*Ks* = 1.56), consistent with previous studies [10], and one recent WGD event (*Ks* = 0.07), which could be detected due to increased genome completeness (Fig. 5B). The insertion of Long Terminal Repeat Retrotransposons (LTR-RTs) plays a crucial role in the evolution of plant genomes [47,48]. The study analyzed the insertion times and genomic positions of LTR-RTs identified in LHH and Newhall. Results indicated that LTR-RTs in both navel orange genomes had been continuously amplifying over the past ~5 Mya and exhibited a recent burst of insertions. A similar pattern was observed in the closely related species *C. clementina*. These LTR-RT insertions were widespread across sweet orange chromosomes and were also found within euchromatic regions, likely due to the ongoing amplification of LTR-RTs [32]. Furthermore, certain regions exhibited a high-density distribution of recent insertions, suggesting the presence of active LTR-RTs in these areas (Fig. 5C).

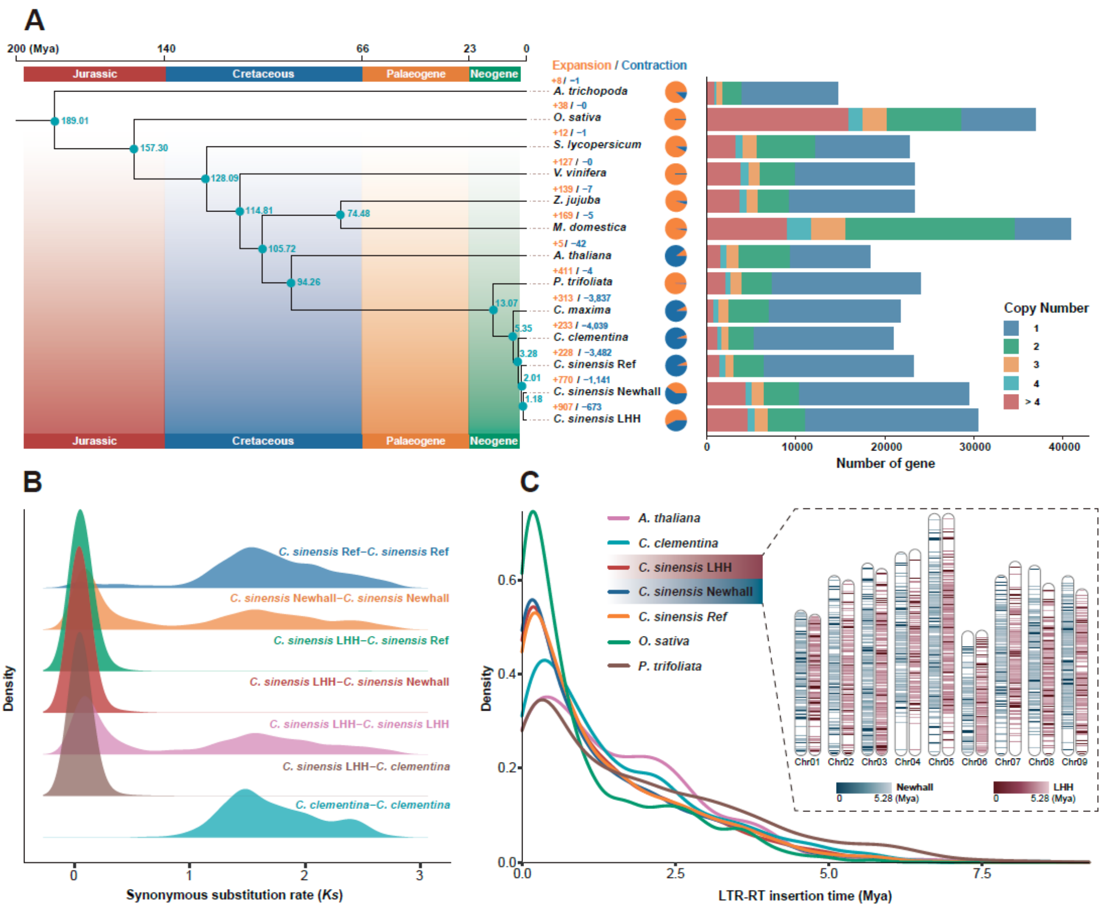

Figure 5: The evolutionary history of *C. sinensis* LHH and Newhall genomes. (A), Phylogenetic tree of *C. sinensis* LHH and Newhall along with 11 other plant genomes. Pie charts depict the expansion and contraction of gene families, with orange indicating expansion and blue indicating contraction. Corresponding values are displayed in matching colors above each chart. Nodes on the evolutionary tree are labeled with estimated divergence times (in Mya). The number of gene copies in each species' genome is shown on the right side. (B), The distribution of *Ks* values illustrates WGD events in the evolution of the *C. sinensis* LHH and Newhall genomes. (C), The distribution of full-length LTR-RT insertions in LHH and Newhall genomes. The density plot illustrates the temporal distribution of full-length LTR-RT insertions in plant genomes, including LHH and Newhall genomes. The chromosome heatmap provides a comprehensive view of the spatial distribution and temporal information of full-length LTR-RT insertions on each chromosome of LHH and Newhall genomes.

Genomic variation between the LHH and Newhall genomes

To elucidate the sequence differences between the genomes of navel orange LHH and Newhall, and to provide a foundational dataset for understanding their phenotypic diversity, collinear regions and variations between the two genomes were identified. Based on alignment results from Mummer (v4.0.0rc1) [49], collinearity analysis was conducted between the genomes, and single-nucleotide polymorphisms (SNPs), as well as presence/absence variations (PAVs), were called. Subsequently, GenomeSyn (v1.2.7) [50] was used to integrate and present these data. Results revealed that 6,075 collinear blocks were identified between the two genomes, with lengths of collinear blocks being 289.07 Mb and 289.54 Mb in the LHH and Newhall genomes, respectively. These blocks account for 372.71 Mb and 370.23 Mb, or 77.56% and 78.21% of the respective genomes, indicating that most regions in both genomes are conserved. The density of SNPs showed differential distributions across the chromosomes of the LHH and Newhall genomes. Chromosomes 3, 5, and 9 were observed to have an abundance of SNPs, with chromosome 9 containing the highest number at 410,667 (Fig. 6A). Additionally, these three chromosomes exhibited more PAVs compared to the other chromosomes. Genome structural variations (SVs) can affect gene expression through various mechanisms [51] and have been reported to contribute to phenotypic diversity in eukaryotes, driving the diversity of functional genes in crops [52]. SVs are considered to have a greater impact on gene expression and protein function compared to SNPs [53]. In this study, MUMandCo (v3.8) [54] was utilized to detect and classify structural variations from Mummer's alignment results. A total of 2,886 SVs were detected between the LHH and Newhall genomes, with insertions and deletions being the most common types of structural variations, numbering 1,383 and 1,313, respectively (Fig. 6B). The distribution and types

of SVs on different chromosomes revealed that chromosomes 5, 6, and 9 had a higher number of structural variations. Genome-wide analysis indicated that insertions and deletions were the predominant types of structural variations. To further annotate these structural variations relative to their positions in the Newhall genome, ANNOVAR [55] was employed. Results showed significant differences in the proportion of each type of SV on different chromosomes. Chromosomes 5, 6, and 9 had the highest proportion of SVs located in exon regions, with 338, 203, and 220 SVs, respectively, accounting for 31.98%, 25.66%, and 31.38% of the total SVs on each chromosome (Fig. 6C). These findings indicate substantial genomic structural diversity between the LHH and Newhall genomes. The extensive variation information within these two genomes offers valuable resources for identifying and utilizing alleles associated with superior agronomic traits.

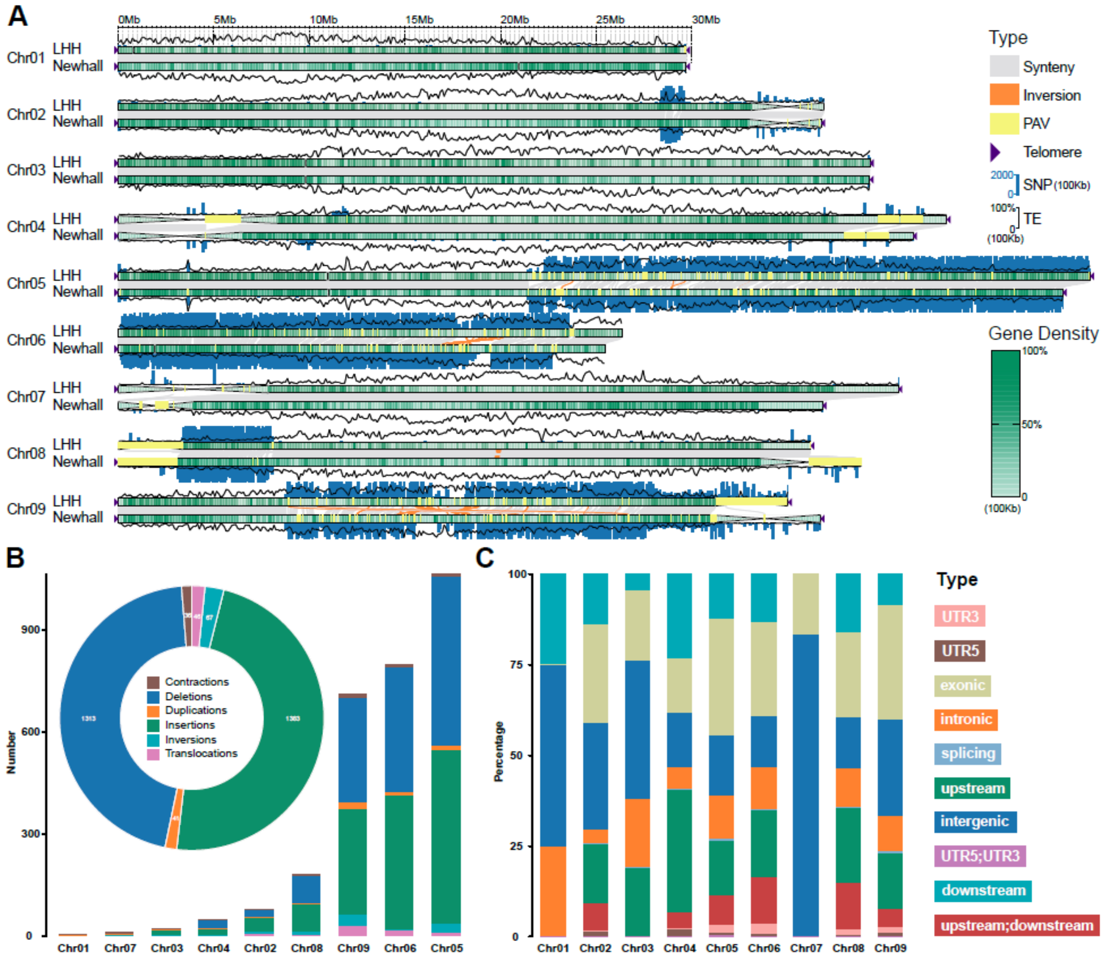

Figure 6: Variations between LHH and Newhall genomes. (A), Collinearity between LHH and Newhall genomes. Collinear regions are connected by grey lines, while inversion regions are connected by orange lines. PAV regions are represented by yellow blocks. Black triangles indicate detected telomere repeat sequences. Gene density heatmaps are plotted on each chromosome with a 100-kb unit. SNP and TE percentage distribution are plotted above or below each chromosome in 100-kb units. (B), Composition of SVs between LHH and NHE genomes. The pie chart shows the proportion of different types of SVs across the whole genome. The stacked bar plot shows the types and quantities of SVs on each chromosome. (C), Positional distribution of SVs on each chromosome relative to the Newhall gene. This study gathered previously reported cold-resistant genes in rice and mapped them onto the Newhall genome using BLASTp [56]. By integrating information on detected

SVs, several homologous genes were found to have SVs in their upstream, downstream, or intronic regions. These SVs may be linked to the differences in cold resistance between Newhall and LHH (Table S10).

Reuse potential

This study successfully assembled two T2T reference genomes for navel orange, providing high-quality genomic resources for future multi-omics and molecular biology research in sweet orange. These reference genomes will significantly enhance the utilization of multi-omics data and improve the accuracy of downstream analyses.

The two navel orange varieties selected for this research exhibit notable differences in important agronomic traits such as flesh color, photosynthetic efficiency, and cold tolerance. Future studies can leverage the high-quality reference genomes generated in this study to construct a pangenome for sweet orange. By incorporating phenotypic information from representative materials, further screening of the various types of variations identified between the LHH and Newhall genomes in this study, including SNPs, SVs, and PAVs, can be undertaken. Subsequent in vivo and in vitro experiments can validate the impact of these structural variations on phenotypes, allowing for the characterization of genes related to important agronomic traits and the understanding of how these variations influence gene function.

Additionally, forthcoming research can use the two T2T genomes as references to analyze population resequencing data of sweet orange. Genome-wide association studies (GWAS) based on SNPs, PAVs, and SVs can then be employed to identify and exploit genomic variations with substantial effects.

Finally, high-quality genomes are crucial for accurate gene prediction. The T2T genomes of the two navel orange varieties will facilitate further improvement in gene annotation quality. Future studies can refine sweet orange gene annotations using full-length transcriptome sequencing and manually validate and correct the annotation result.

Discussion

In this study, we performed de novo assembly and annotation of the genomes of two navel orange varieties that differ in key agronomic traits such as flesh color, photosynthetic efficiency, and cold tolerance. We utilized a comprehensive approach integrating PacBio HiFi, ONT UL, Hi-C, and Illumina PE sequencing data. With the support of advanced sequencing technologies and genome assembly algorithms and strategies, we successfully assembled two T2T reference genomes for sweet orange, achieving high continuity, completeness, and accuracy. These assemblies have unveiled the sequences of highly repetitive regions, including centromeres and telomeres, within the sweet orange genomes. Through comparative genomic analysis, we elucidated the evolutionary history of the sweet orange genomes, including WGD events, gene family expansions and contractions, divergence times between species, and the amplification of LTR-RTs. Collinear regions account for over 77% of the sequences between the two genomes, indicating significant conservation. On the other hand, extensive intraspecies variations were detected, including 2,886 structural variations (SVs). We categorized these SVs and mapped their relative positions in the genome. These findings provide a valuable set of candidate genes, narrowing the scope for subsequent studies aimed at characterizing and validating genes associated with agronomic trait differences between the two sweet orange varieties. This study lays a high-quality data foundation for understanding the phenotypic and genetic diversity of sweet

oranges.

The pangenome is a current research hotspot in plant genomics. Gap-free genomes can serve as high-quality references for constructing more comprehensive and accurate graph-based pangenomes, which, in turn, facilitate the study of polymorphisms in complex genomic regions [57]. The two T2T sweet orange reference genomes provided in this study enhance the existing sweet orange genomic resources. These genomes will support the construction of a graph-based pangenome for sweet orange, enable the detection and genotyping of a broader range of structural variations (SVs), and, when combined with population resequencing data, help uncover the associations between sweet orange phenotypes and genotypes.

Data Availability

The genome sequences and raw sequencing data for the Newhall and LHH navel orange genomes are available under NCBI BioProject ID PRJNA1122682. The raw sequencing data for the Newhall and LHH navel orange genomes are available from the National Genomics Data Center (NGDC, https://bigd.big.ac.cn) [58], under accession number PRJCA026660. All supporting data can be found in the GigaScience GigaDB database [59].

Additional Files

Supplementary Figure 1. BUSCO assessment results for the genomes of LHH and Newhall.

Supplementary Figure 2. Statistics of integrated gene models predicted by three strategies. The Venn diagram depicts the quantities of gene models predicted by three strategies in LHH (A) and Newhall (B) genomes, along with their overlapping relationships.

Supplementary Figure 3. Gene family clustering of 13 plant genomes. The Venn diagrams show unique and shared orthologous gene clusters among the 13 species (A) and the 5 Citrus genomes (B).

Supplementary Table 1. Statistics of Hi-C assembly results for the Newhall and LHH genomes

Supplementary Table 2. Telomere location information for Newhall and LHH genomes

Supplementary Table 3. Centromere location information for Newhall and LHH genomes

Supplementary Table 4. Summary of BUSCO assessment results for LHH and Newhall genomes

Supplementary Table 5. Mapping rate statistics of sequencing data for LHH and Newhall genomes

Supplementary Table 6. Statistical information of TEs in LHH and Newhall genomes

Supplementary Table 7. Gene model annotation results of LHH and Newhall genomes

Supplementary Table 8. Summary of non-coding RNA annotation results in LHH and Newhall genomes

Supplementary Table 9. Statistical summary of gene family clustering results

Supplementary Table 10. SVs of homologous genes in the Newhall genome corresponding to reported cold tolerance genes in *O. sativa* Nipponbare

Abbreviations

T2T: telomere-to-telomere; Mb: megabases; Gb: gigabase; ONT UL: Oxford Nanopore Technologies Ultra Long; HiFi: High Fidelity; Hi-C: high-throughput chromosome conformation capture; CCS: Circular Consensus Sequencing; PE: pair end; Q-PCR: quantitative polymerase chain reaction; LTR: long terminal repeat; LAI: LTR Assembly Index; QV: quality value; TEs: transposable elements; CDS: coding sequence; tRNA: transfer RNA; miRNA: microRNA; snoRNA: small nucleolar RNA; snRNA: small nuclear RNA; MSA: multiple sequence alignment; ML: maximum

likelihood; Mya: million years ago; WGD: whole genome duplication; *Ks*: synonymous substitution rates; SNPs: single-nucleotide polymorphisms; PAVs: presence/absence variations; SVs: structural variations; GWAS: genome-wide association studies.

Competing Interests

The authors declare that they have no competing interests.

Funding

This work was financed by Ministry of Agriculture and rural citrus industry cluster project and Chongqing academy of agricultural sciences municipal financial special project (NKY-2022AB005)

Authors' Contributions

L.H. and J.-M.S. conceived the idea, supervised the work, and revised the manuscript. M.W., S.L. and H.Y. prepared the plant materials. L.H., J.-M.S., X.-D.X., L.Y., S.-Y.Y. and L.-L.C. analyzed the data. L.H., J.-M.S., X.-D.X. and L.Y. wrote the original draft and revised the manuscript. L.H., X.-D.X. and L.Y. contributed equally to this work. All authors have read and approved the final manuscript.

Acknowledgments

We thank Biomarker Technologies Co., Ltd for assisting in sequencing.

References

1. Safdar MN, Kausar T, Jabbar S, Mumtaz A, Ahad K, Saddozai AA. Extraction and quantification of

polyphenols from kinnow (Citrus reticulate L.) peel using ultrasound and maceration techniques. *J Food Drug Anal*. 2017; doi: 10.1016/j.jfda.2016.07.010.

2. Zou Z, Xi W, Hu Y, Nie C, Zhou Z. Antioxidant activity of Citrus fruits. *Food Chem*. 2016; doi: 10.1016/j.foodchem.2015.09.072.

3. Farag MA, Abib B, Ayad L, Khattab AR. Sweet and bitter oranges: An updated comparative review of their bioactives, nutrition, food quality, therapeutic merits and biowaste valorization practices. *Food Chem*. 2020; doi: 10.1016/j.foodchem.2020.127306.

4. Seminara S, Bennici S, Di Guardo M, Caruso M, Gentile A, La Malfa S, et al.. Sweet Orange: Evolution, Characterization, Varieties, and Breeding Perspectives. *Agriculture*. Multidisciplinary Digital Publishing Institute; 2023; doi: 10.3390/agriculture13020264.

5. Carlos Merino, Aurea Hervalejo, Arturo Salguero, David GonzÃ¡lez, Francisco J. Arenas-Arenas. YIELD AND FRUIT QUALITY OF TWO EARLY MATURING ORANGE CULTIVARS, “NAVELINA” AND “FUKUMOTO”, IN ANDALUSIA, SPAIN. *Acta Horticulturae*. International Society for Horticultural Science (ISHS), Leuven, Belgium;

6. Wu GA, Terol J, Ibanez V, López-García A, Pérez-Román E, Borredá C, et al.. Genomics of the origin and evolution of Citrus. *Nature*. Nature Publishing Group; 2018; doi: 10.1038/nature25447.

7. Wang L, Huang Y, Liu Z, He J, Jiang X, He F, et al.. Somatic variations led to the selection of acidic and acidless orange cultivars. *Nat Plants*. Nature Publishing Group; 2021; doi: 10.1038/s41477-021-00941-x.

8. The Arabidopsis Genome Initiative. Analysis of the genome sequence of the flowering plant Arabidopsis thaliana. *Nature*. Nature Publishing Group; 2000; doi: 10.1038/35048692.

9. Shendure J, Balasubramanian S, Church GM, Gilbert W, Rogers J, Schloss JA, et al.. DNA sequencing at 40: past, present and future. *Nature*. 2017; doi: 10.1038/nature24286.

10. Xu Q, Chen L-L, Ruan X, Chen D, Zhu A, Chen C, et al.. The draft genome of sweet orange (Citrus sinensis). *Nat Genet*. Nature Publishing Group; 2013; doi: 10.1038/ng.2472.

11. Wu GA, Prochnik S, Jenkins J, Salse J, Hellsten U, Murat F, et al.. Sequencing of diverse mandarin, pummelo and orange genomes reveals complex history of admixture during citrus domestication. *Nat Biotechnol*. 2014; doi: 10.1038/nbt.2906.

12. Wang X, Xu Y, Zhang S, Cao L, Huang Y, Cheng J, et al.. Genomic analyses of primitive, wild and cultivated citrus provide insights into asexual reproduction. *Nat Genet*. 2017; doi: 10.1038/ng.3839.

13. Huang Y, He J, Xu Y, Zheng W, Wang S, Chen P, et al.. Pangenome analysis provides insight into the evolution of the orange subfamily and a key gene for citric acid accumulation in citrus fruits. *Nat Genet*. Nature Publishing Group; 2023; doi: 10.1038/s41588-023-01516-6.

14. Bao Y, Zeng Z, Yao W, Chen X, Jiang M, Sehrish A, et al.. A gap-free and haplotype-resolved lemon genome provides insights into flavor synthesis and huanglongbing (HLB) tolerance. *Hortic Res*. 2023; doi: 10.1093/hr/uhad020.

15. Yang L, Deng H, Wang M, Li S, Wang W, Yang H, et al.. A high-quality chromosome-scale genome

assembly of blood orange, an important pigmented sweet orange variety. *Sci Data*. 2024; doi: 10.1038/s41597-024-03313-0.

16. Marçais G, Kingsford C. A fast, lock-free approach for efficient parallel counting of occurrences of k-mers. *Bioinformatics*. 2011; doi: 10.1093/bioinformatics/btr011.

17. Ranallo-Benavidez TR, Jaron KS, Schatz MC. GenomeScope 2.0 and Smudgeplot for reference-free profiling of polyploid genomes. *Nat Commun*. 2020; doi: 10.1038/s41467-020-14998-3.

18. Cheng H, Concepcion GT, Feng X, Zhang H, Li H. Haplotype-resolved de novo assembly using phased assembly graphs with hifiasm. *Nat Methods*. 2021; doi: 10.1038/s41592-020-01056-5.

19. Burton JN, Adey A, Patwardhan RP, Qiu R, Kitzman JO, Shendure J. Chromosome-scale scaffolding of de novo genome assemblies based on chromatin interactions. *Nat Biotechnol*. Nature Publishing Group; 2013; doi: 10.1038/nbt.2727.

20. Durand NC, Robinson JT, Shamim MS, Machol I, Mesirov JP, Lander ES, et al.. Juicebox Provides a Visualization System for Hi-C Contact Maps with Unlimited Zoom. *Cell Syst*. 2016; doi: 10.1016/j.cels.2015.07.012.

21. Xu M, Guo L, Gu S, Wang O, Zhang R, Peters BA, et al.. TGS-GapCloser: A fast and accurate gap closer for large genomes with low coverage of error-prone long reads. *Gigascience*. 2020; doi: 10.1093/gigascience/giaa094.

22. Xie L, Gong X, Yang K, Huang Y, Zhang S, Shen L, et al.. Technology-enabled great leap in deciphering plant genomes. *Nat Plants*. Nature Publishing Group; 2024; doi: 10.1038/s41477-

024-01655-6.

23. Seppey M, Manni M, Zdobnov EM. BUSCO: Assessing Genome Assembly and Annotation Completeness. *Methods Mol Biol*. 2019; doi: 10.1007/978-1-4939-9173-0_14.

24. Parra G, Bradnam K, Korf I. CEGMA: a pipeline to accurately annotate core genes in eukaryotic genomes. *Bioinformatics*. 2007; doi: 10.1093/bioinformatics/btm071.

25. Li H. Minimap2: pairwise alignment for nucleotide sequences. *Bioinformatics*. 2018; doi: 10.1093/bioinformatics/bty191.

26. Ou S, Jiang N. LTR_retriever: A Highly Accurate and Sensitive Program for Identification of Long Terminal Repeat Retrotransposons. *Plant Physiol*. 2018; doi: 10.1104/pp.17.01310.

27. Ou S, Chen J, Jiang N. Assessing genome assembly quality using the LTR Assembly Index (LAI). *Nucleic Acids Res*. 2018; doi: 10.1093/nar/gky730.

28. Rhie A, Walenz BP, Koren S, Phillippy AM. Merqury: reference-free quality, completeness, and phasing assessment for genome assemblies. *Genome Biol*. 2020; doi: 10.1186/s13059-020-02134-9.

29. Flynn JM, Hubley R, Goubert C, Rosen J, Clark AG, Feschotte C, et al.. RepeatModeler2 for automated genomic discovery of transposable element families. *Proc Natl Acad Sci U S A*. 2020; doi: 10.1073/pnas.1921046117.

30. Bao W, Kojima KK, Kohany O. Repbase Update, a database of repetitive elements in eukaryotic genomes. *Mob DNA*. 2015; doi: 10.1186/s13100-015-0041-9.

31. Tarailo-Graovac M, Chen N. Using RepeatMasker to identify repetitive elements in genomic sequences. *Curr Protoc Bioinformatics*. 2009; doi: 10.1002/0471250953.bi0410s25.

32. Song J-M, Guan Z, Hu J, Guo C, Yang Z, Wang S, et al.. Eight high-quality genomes reveal pan-genome architecture and ecotype differentiation of Brassica napus. *Nat Plants*. 2020; doi: 10.1038/s41477-019-0577-7.

33. Wolff J, Rabbani L, Gilsbach R, Richard G, Manke T, Backofen R, et al.. Galaxy HiCExplorer 3: a web server for reproducible Hi-C, capture Hi-C and single-cell Hi-C data analysis, quality control and visualization. *Nucleic Acids Res*. 2020; doi: 10.1093/nar/gkaa220.

34. Huerta-Cepas J, Szklarczyk D, Heller D, Hernández-Plaza A, Forslund SK, Cook H, et al.. eggNOG 5.0: a hierarchical, functionally and phylogenetically annotated orthology resource based on 5090 organisms and 2502 viruses. *Nucleic Acids Res*. 2019; doi: 10.1093/nar/gky1085.

35. Kanehisa M, Sato Y, Kawashima M, Furumichi M, Tanabe M. KEGG as a reference resource for gene and protein annotation. *Nucleic Acids Res*. 2016; doi: 10.1093/nar/gkv1070.

36. Boeckmann B, Bairoch A, Apweiler R, Blatter M-C, Estreicher A, Gasteiger E, et al.. The SWISS-PROT protein knowledgebase and its supplement TrEMBL in 2003. *Nucleic Acids Res*. 2003; doi: 10.1093/nar/gkg095.

37. Finn RD, Mistry J, Schuster-Böckler B, Griffiths-Jones S, Hollich V, Lassmann T, et al.. Pfam: clans, web tools and services. *Nucleic Acids Res*. 2006; doi: 10.1093/nar/gkj149.

38. Lowe TM, Eddy SR. tRNAscan-SE: a program for improved detection of transfer RNA genes in

genomic sequence. *Nucleic Acids Res*. 1997; doi: 10.1093/nar/25.5.955.

39. Griffiths-Jones S, Moxon S, Marshall M, Khanna A, Eddy SR, Bateman A. Rfam: annotating non-coding RNAs in complete genomes. *Nucleic Acids Res*. 2005; doi: 10.1093/nar/gki081.

40. Nawrocki EP, Eddy SR. Infernal 1.1: 100-fold faster RNA homology searches. *Bioinformatics*. 2013; doi: 10.1093/bioinformatics/btt509.

41. Emms DM, Kelly S. OrthoFinder: solving fundamental biases in whole genome comparisons dramatically improves orthogroup inference accuracy. *Genome Biol*. 2015; doi: 10.1186/s13059-015-0721-2.

42. Katoh K, Misawa K, Kuma K, Miyata T. MAFFT: a novel method for rapid multiple sequence alignment based on fast Fourier transform. *Nucleic Acids Res*. 2002; doi: 10.1093/nar/gkf436.

43. Castresana J. Selection of conserved blocks from multiple alignments for their use in phylogenetic analysis. *Mol Biol Evol*. 2000; doi: 10.1093/oxfordjournals.molbev.a026334.

44. Minh BQ, Schmidt HA, Chernomor O, Schrempf D, Woodhams MD, von Haeseler A, et al.. IQ-TREE 2: New Models and Efficient Methods for Phylogenetic Inference in the Genomic Era. *Mol Biol Evol*. 2020; doi: 10.1093/molbev/msaa015.

45. Yang Z. PAML 4: phylogenetic analysis by maximum likelihood. *Mol Biol Evol*. 2007; doi: 10.1093/molbev/msm088.

46. De Bie T, Cristianini N, Demuth JP, Hahn MW. CAFE: a computational tool for the study of gene family evolution. *Bioinformatics*. 2006; doi: 10.1093/bioinformatics/btl097.

47. Li S-F, She H-B, Yang L-L, Lan L-N, Zhang X-Y, Wang L-Y, et al.. Impact of LTR-Retrotransposons on Genome Structure, Evolution, and Function in Curcurbitaceae Species. *International Journal of Molecular Sciences*. Multidisciplinary Digital Publishing Institute; 2022; doi: 10.3390/ijms231710158.

48. Choi JY, Purugganan MD. Evolutionary Epigenomics of Retrotransposon-Mediated Methylation Spreading in Rice. *Mol Biol Evol*. 2018; doi: 10.1093/molbev/msx284.

49. Marçais G, Delcher AL, Phillippy AM, Coston R, Salzberg SL, Zimin A. MUMmer4: A fast and versatile genome alignment system. *PLoS Comput Biol*. 2018; doi: 10.1371/journal.pcbi.1005944.

50. Zhou Z-W, Yu Z-G, Huang X-M, Liu J-S, Guo Y-X, Chen L-L, et al.. GenomeSyn: a bioinformatics tool for visualizing genome synteny and structural variations. *J Genet Genomics*. 2022; doi: 10.1016/j.jgg.2022.03.013.

51. Hollox EJ, Zuccherato LW, Tucci S. Genome structural variation in human evolution. *Trends Genet*. 2022; doi: 10.1016/j.tig.2021.06.015.

52. Chawla HS, Lee H, Gabur I, Vollrath P, Tamilselvan-Nattar-Amutha S, Obermeier C, et al.. Long-read sequencing reveals widespread intragenic structural variants in a recent allopolyploid crop plant. *Plant Biotechnology Journal*. 2021; doi: 10.1111/pbi.13456.

53. Yuan Y, Bayer PE, Batley J, Edwards D. Current status of structural variation studies in plants. *Plant Biotechnology Journal*. 2021; doi: 10.1111/pbi.13646.

54. O'Donnell S, Fischer G. MUM&Co: accurate detection of all SV types through whole-genome

alignment. *Bioinformatics*. 2020; doi: 10.1093/bioinformatics/btaa115.

55. Wang K, Li M, Hakonarson H. ANNOVAR: functional annotation of genetic variants from high-throughput sequencing data. *Nucleic Acids Res*. 2010; doi: 10.1093/nar/gkq603.

56. Cock PJA, Chilton JM, Grüning B, Johnson JE, Soranzo N. NCBI BLAST+ integrated into Galaxy. *Gigascience*. 2015; doi: 10.1186/s13742-015-0080-7.

57. Wang S, Qian Y-Q, Zhao R-P, Chen L-L, Song J-M. Graph-based pan-genomes: increased opportunities in plant genomics. *J Exp Bot*. 2023; doi: 10.1093/jxb/erac412.

58. CNCB-NGDC Members and Partners. Database Resources of the National Genomics Data Center, China National Center for Bioinformation in 2023. *Nucleic Acids Res*. 2023; doi: 10.1093/nar/gkac1073.

59. Sneddon TP, Li P, Edmunds SC. GigaDB: announcing the GigaScience database. *GigaScience*. 2012; doi: 10.1186/2047-217X-1-11.

Figure 1

Click here to access/download;Figure;Figure 1.pdf

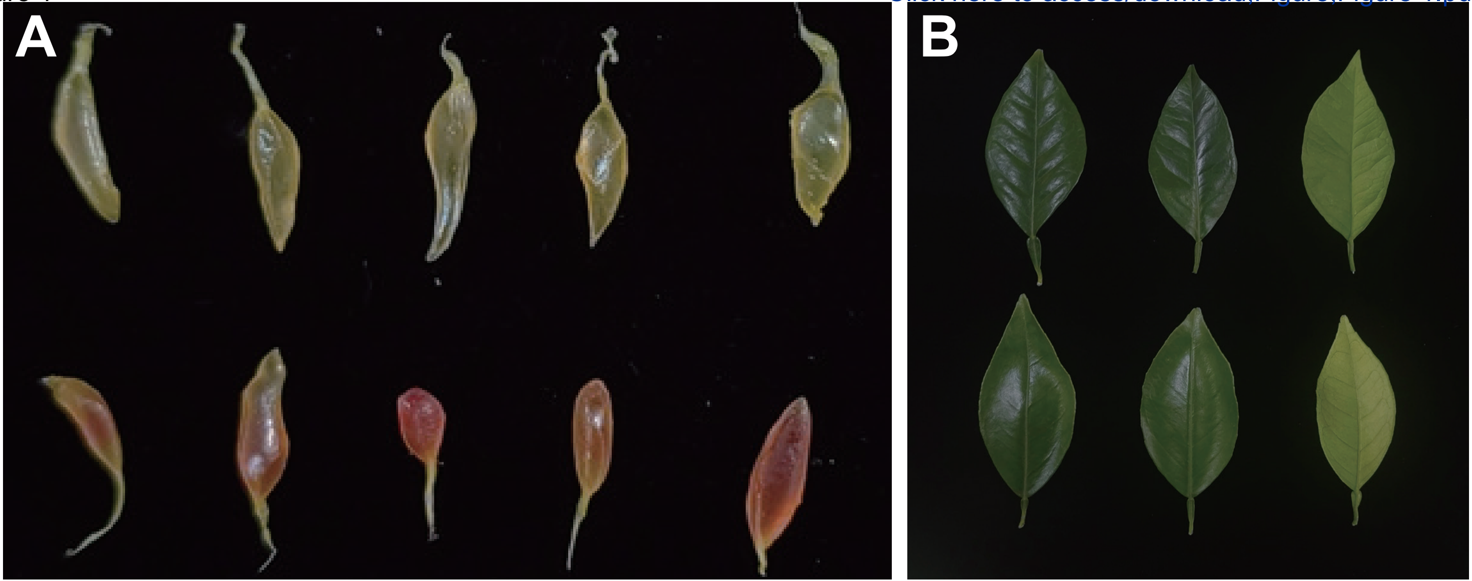

Figure 2

Click here to access/download;Figure;Figure 2.pdf

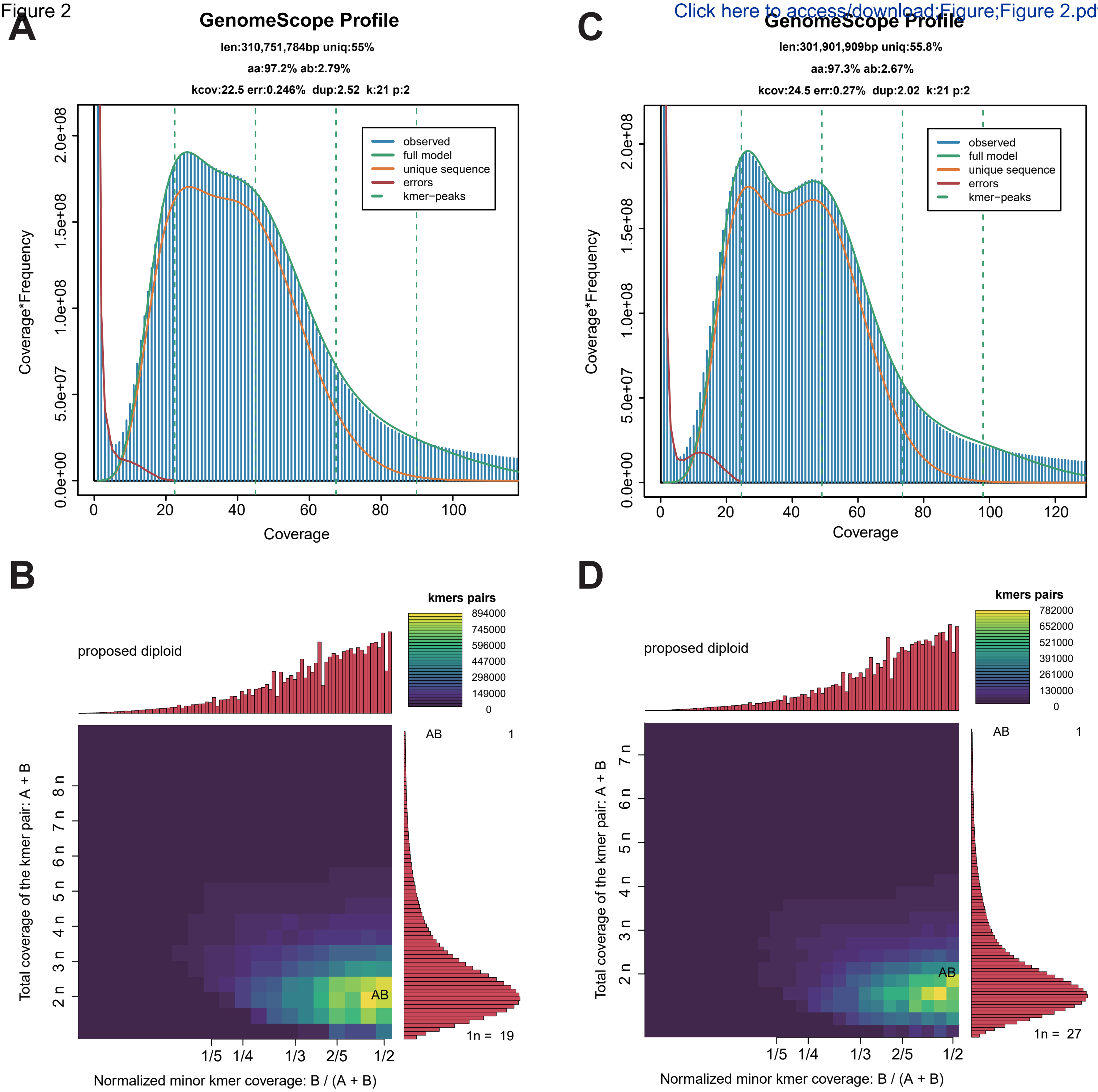

Figure 3

Click here to access/download;Figure;Figure 3.pdf

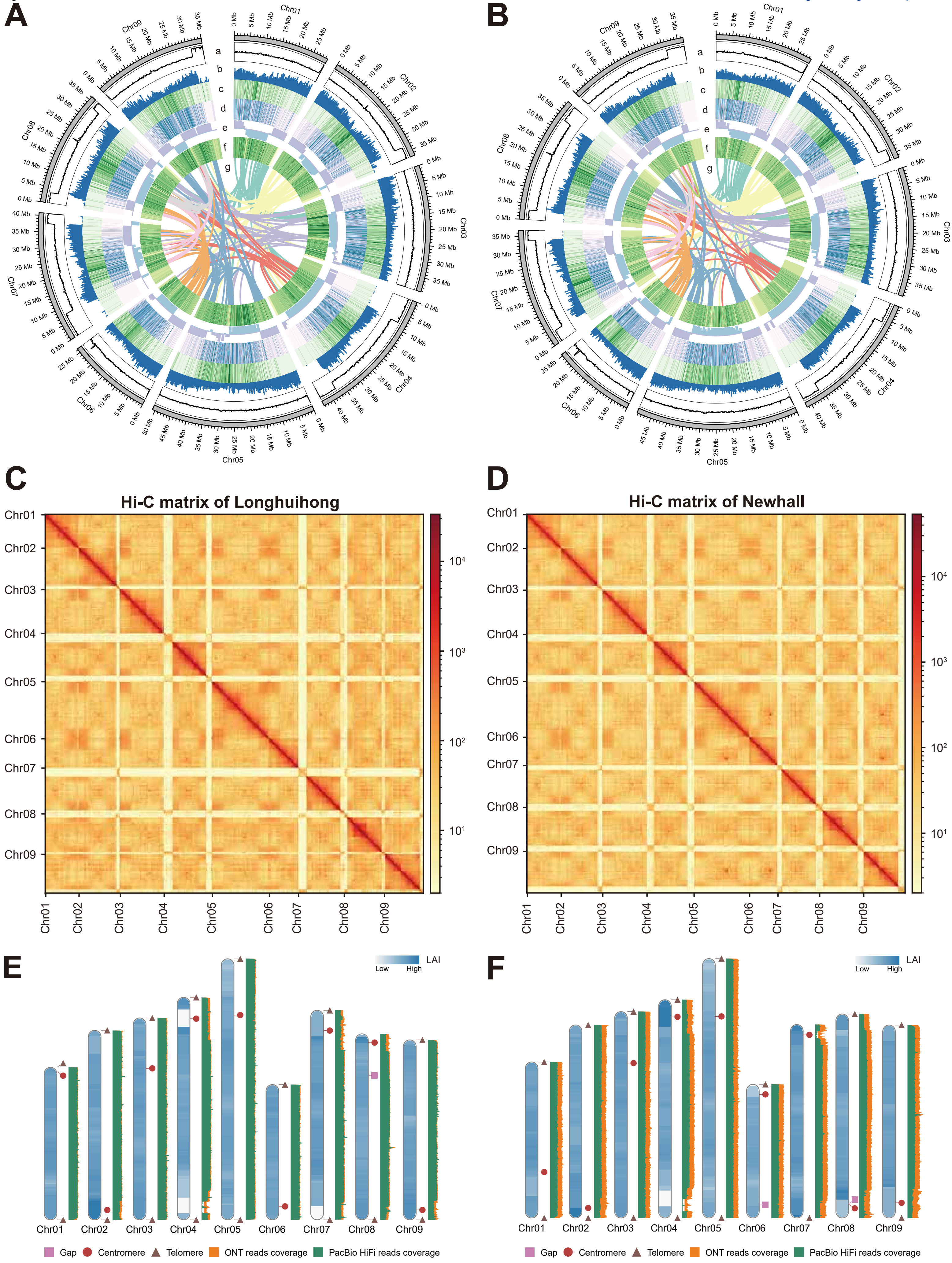

Figure 4

Click here to access/download;Figure;Figure 4.pdf

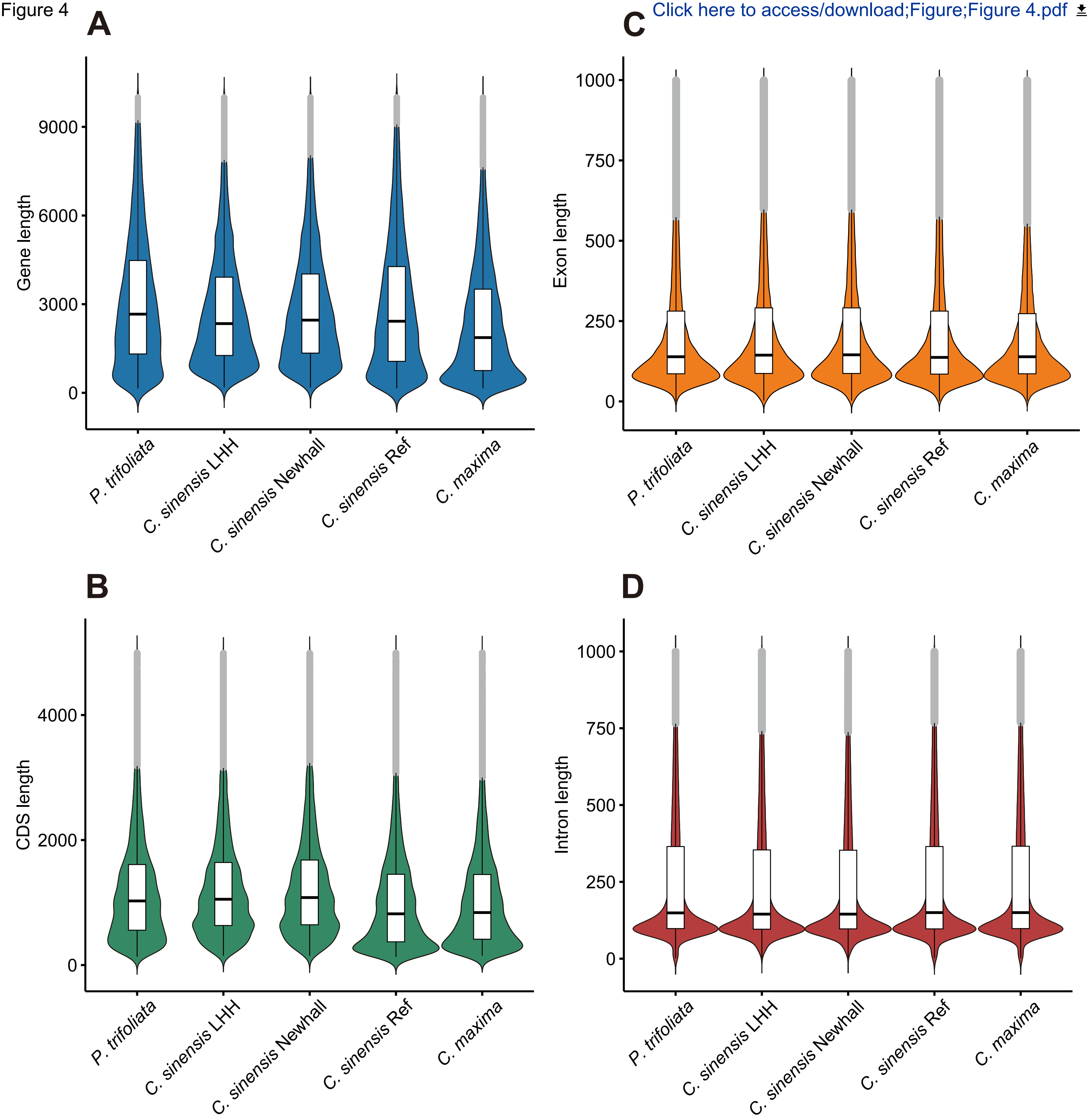

Figure 5

Click here to access/download;Figure;Figure 5.pdf

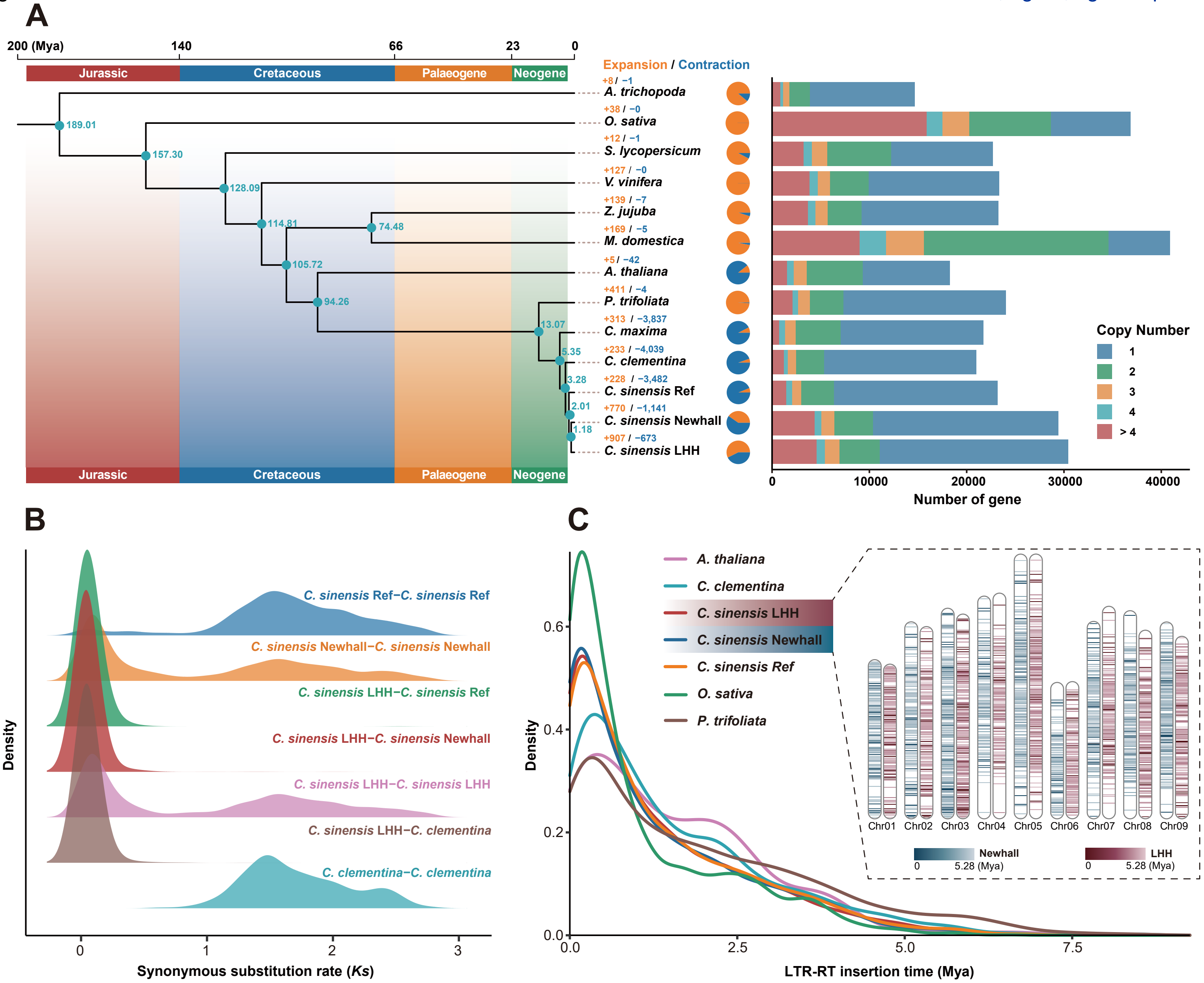

Figure 6

Click here to access/download;Figure;Figure 6.pdf

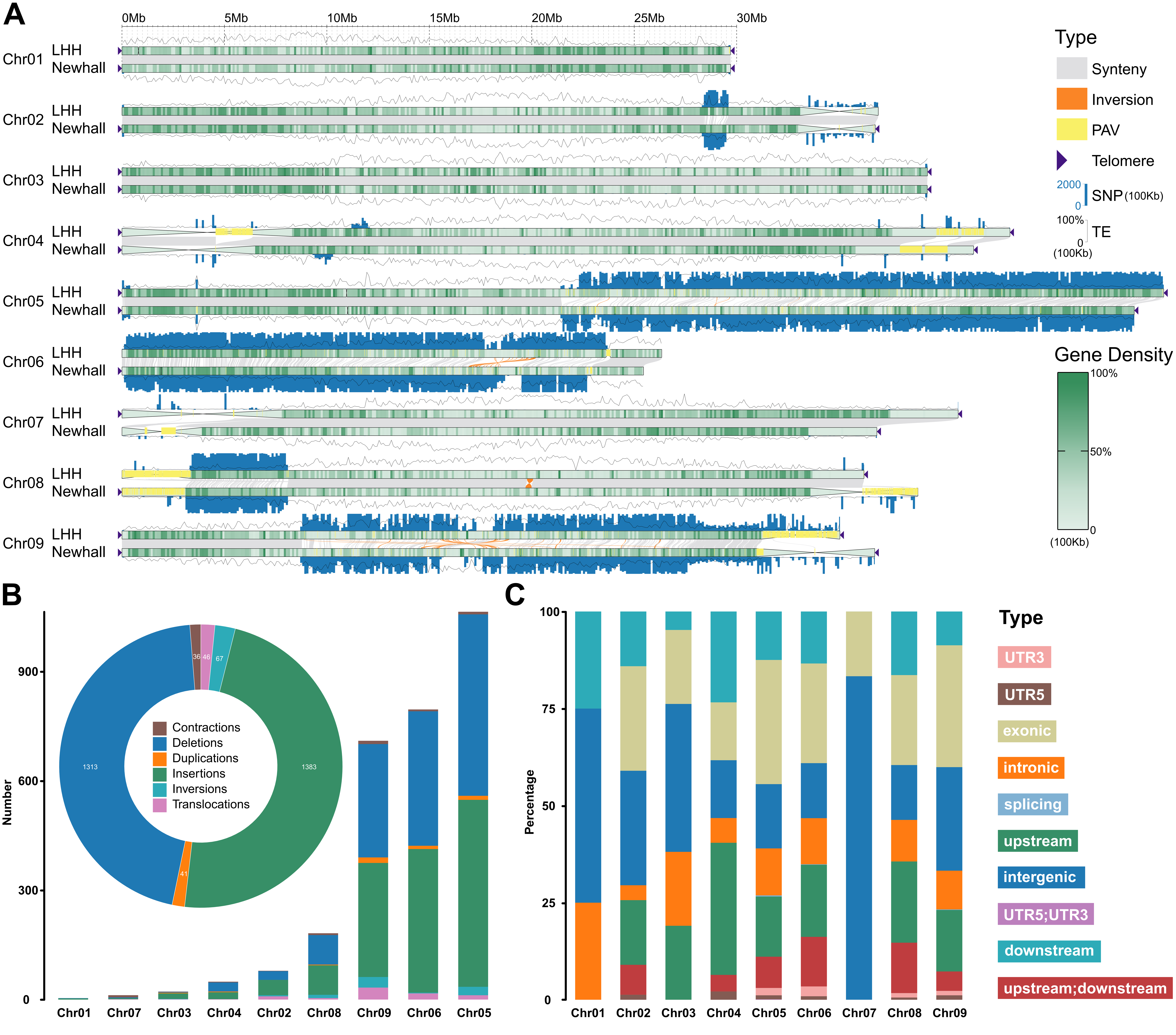

Supplementary Figure 1

Click here to access/download
Supplementary Material
Supplementary Figure 1.pdf

Supplementary Figure 2

Click here to access/download
Supplementary Material
Supplementary Figure 2.pdf

Supplementary Figure 3

Click here to access/download
Supplementary Material
Supplementary Figure 3.pdf

Supplementary Tables 1-10

Click here to access/download
Supplementary Material
Supplementary Tables 1-10.xlsx
