# Supplementary material for: Construction and analysis of telomere-to-telomere genomes for 2 sweet oranges: Longhuihong and Newhall (Citrus sinensis)
Source: Gigascience. 2024 Nov 26;13:giae084. doi: 10.1093/gigascience/giae084 (PMC11590112; doi:10.1093/gigascience/giae084)
Supplement: giae084_GIGA-D-24-00206_Revision_1 [file giae084_giga-d-24-00206_revision_1.pdf]

GigaScience

Construction and analysis of telomere-to-telomere genomes for two sweet oranges: Longhuihong and Newhall (Citrus sinensis)

--Manuscript Draft--

**Manuscript Number:**	GIGA-D-24-00206R1
**Full Title:**	Construction and analysis of telomere-to-telomere genomes for two sweet oranges: Longhuihong and Newhall (Citrus sinensis)
**Article Type:**	Data Note
**Funding Information:**	Ministry of Agriculture and rural citrus industry cluster project and Chongqing academy of agricultural sciences municipal financial special project (NKY-2022AB005) — Dr. Lin Hong
**Abstract:**	Background<br><br>Sweet orange (Citrus sinensis Osbeck) is a fruit crop of high nutritional value that is widely consumed around the world. However, its susceptibility to low-temperature stress limits its cultivation and production in regions prone to frost damage, severely impacting the sustainable development of the sweet orange industry. Therefore, developing cold-resistant sweet orange varieties is of great necessity. Traditional hybrid breeding methods are not feasible due to the polyembryonic phenomenon in sweet oranges, necessitating the enhancement of its germplasm through molecular breeding. High-quality reference genomes are valuable for studying crop resistance to biotic and abiotic stresses. However, the lack of genomic resources for cold-resistant sweet orange varieties has hindered the progress in developing such varieties and researching their molecular mechanisms of cold resistance.<br><br>Findings<br><br>This study integrated PacBio HiFi, ONT, Hi-C, and Illumina sequencing data to assemble telomere-to-telomere (T2T) reference genomes for the cold-resistant sweet orange mutant 'Longhuihong' (Citrus sineses [L.] Osb. cv. LHH) and its wild-type counterpart 'Newhall' (Citrus sineses [L.] Osb. cv. Newhall). Comprehensive evaluations based on multiple criteria revealed that both genomes exhibit high continuity, completeness, and accuracy. The genome sizes were 340.28 Mb and 346.33 Mb, with contig N50 of 39.31 Mb and 36.77 Mb, respectively. In total, 31,456 and 30,021 gene models were annotated in the respective genomes. Leveraging these assembled genomes, comparative genomics analyses were performed, elucidating the evolutionary history of the sweet orange genome. Moreover, the study identified 2,886 structural variants (SVs) between the two genomes, with several SVs located in the upstream, downstream, or intronic regions of homologous genes known to be associated with cold resistance.<br><br>Conclusions<br><br>The study de novo assembled two T2T reference genomes of sweet orange varieties exhibiting different levels of cold tolerance. These genomes serve as valuable foundational resources for genomic research and molecular breeding aimed at enhancing cold tolerance in sweet oranges. Additionally, they expand the existing repository of reference genomes and sequencing data resources for Citrus sinensis. Moreover, these genomes provide a critical data foundation for comparative genomics analyses across different plant species.
**Corresponding Author:**	Jia-Ming Song<br>Southwest University<br>Chongqing, CHINA
**Corresponding Author Secondary Information:**	
**Corresponding Author's Institution:**	Southwest University

Powered by Editorial Manager® and ProduXion Manager® from Aries Systems Corporation

**Corresponding Author's Secondary Institution:**	
**First Author:**	Lin Hong
**First Author Secondary Information:**	
**Order of Authors:**	Lin Hong
	Xin-Dong Xu
	Lei Yang
	Min Wang
	Shuang Li
	Haijian Yang
	Si-Ying Ye
	Ling-Ling Chen
	Jia-Ming Song
**Order of Authors Secondary Information:**	
**Response to Reviewers:**	Point-by-point response to reviewers' comments<br>We sincerely appreciate the reviewers for their insightful comments. We have thoroughly reviewed their feedback and made the necessary revisions to the manuscript. All modifications have been highlighted throughout both the revised manuscript and the figures.<br>Reviewer #1:<br>This study has generated two high quality collapsed genomes for two varieties of navel oranges; LHH and Newhall. They have performed the genome assemblies, annotations, structural variations detections, comparison of these two genomes with respect to other available genomes, and WGD analysis. Overall, this work provides valuable genomic resource for future research on sweet orange and other citrus as well.<br>However, the methodology is not clearly and comprehensively explained for each analysis that have been performed here. For ex: If you use a specific tool for a particular analysis, it's always good to mention which type of input data you used (genome assemblies/whole protein or CDS files/the longest protein/CDS files), what are the steps of that analysis, specific threshold values (BLAST e-values).<br>Also, it would be good to indicate which tools the authors used to generate the figures in the figure legends. You can give the tool name and the reference as well. You also can mention it in the methodology and this is important to replicate the things in the future for another research.<br>some clarifications are required as listed below.<br>1. It would be good if you can give details about the method of leaf collections for DNA and RNA extractions, and Hi-C experiments for the other readers to know and replicate them in the future. (ex: collection under dry ice or leaves snap frozen)<br>[Response]: We genuinely appreciate your invaluable suggestions. In response, we have incorporated detailed descriptions of these experimental procedures into the methods section of our manuscript. The revised methodology is outlined below:<br>Healthy, juvenile leaves are carefully selected and their surfaces are thoroughly rinsed with deionized water. After gently drying the leaves to eliminate surface moisture, we precisely cut the tissue into 50-100 mg fragments. These fragments are then placed into pre-prepared 2 mL cryovials and promptly frozen in liquid nitrogen for a duration of 3-4 hours. Following this, the samples are securely stored at -80°C for future DNA and RNA extraction.<br>Hi-C experiment protocol:<br>(1)Collect 1 gram of tender leaves, rinse them thoroughly in icy water, and gently blot them dry with absorbent paper.<br>(2)Using scissors, carefully mince the sample tissue and place it into a 50 mL centrifuge tube.<br>(3)Transfer the minced tissue into a 50 mL centrifuge tube containing 35 mL of NIB Buffer.<br>(4)Introduce 35 μL of PMSF, 35 μL of β-mercaptoethanol, and 2 mL of 36%

formaldehyde into the mixture.
(5)Allow the mixture to react for 90 minutes on a vertical rotator.
(6)Add 2.5 mL of 2M glycine and gently agitate the solution, either manually or on a shaker, for a minimum of 5 minutes to halt the reaction.
(7)Filter out the excess liquid and rinse the sample thoroughly with sterile water until all foam disappears.
(8)Blot the sample dry with absorbent paper, place it in a 50 mL centrifuge tube, and promptly freeze it in liquid nitrogen for future use.
2. Which method you used for DNA extraction? Was it CTAB? that was not mentioned in the manuscript.
[Response]: Thank you for pointing out this issue. The DNA extraction method used in our study is indeed CTAB. We have updated the manuscript to include this information and apologize for the omission.
3. Line 188 – It would be good to indicate the exact version of hifiasm you used for assemblies because each new release has fixed different issues in assemblies and has incorporated new improvements.
[Response]: We sincerely appreciate your feedback. In response, we have included and highlighted the version information for hifiasm (v0.19.8) [1] in the relevant section of our manuscript.
4. Any reason why you didn't generate haplotype-resolved assemblies for these two genomes? HiFiasm can integrate HiC data to give fully phased haplotypes and the haplotype-resolved assemblies are important in detecting the allele specific structural variations in genomes rather than collapsed genomes. The availability of Hi-C data is an added advantage of resolving the phased haplotypes.
[Response]: Thank you sincerely for providing valuable suggestions. Based on your suggestion, we have included the assemblies of the LHH and Newhall haploid genomes (Fig. R1). Using the haplotype-resolved genome assemblies, we have further identified allele-specific structural variations, with a total of 164 such structural variants identified.

Figure R1: Synteny between the LHH and Newhall diploid genomes (https://cbi.gxu.edu.cn/zwzhou/xdxu/Figures/Figure_R1.pdf).

5. Line 213 – 216 You have indicated that "Telomeric sequences were detected at both ends of 7
chromosomes in each genome, while the remaining chromosomes exhibited telomeric sequences at one end.". If that is the case, how you define that these assemblies are T2T level without having telomeric repeats at both ends of all the chromosomes? It seems that two chromosomes of the two varieties don't have telomeric repeat at one end.
[Response]: We sincerely appreciate your valuable suggestions for our research. In this paper, the definition of the T2T genome is based on the recent publication [2], where a reference genome is considered T2T when more than half of its chromosomes have reached the T2T level. It is upon this criterion that we have defined the reference genome used in our study as a T2T genome.
6. Line 240-242 Is there a specific threshold in Merqury tool to identify the completeness of the assemblies? What is the minimum QV value for a complete genome?
[Response]: Thank you for raising valuable questions about our work. According to definitions in the literature, a higher assembly consensus quality value (QV) indicates greater accuracy, with Q30 corresponding to 99.9% accuracy and Q40 to 99.99% accuracy [3]. In our study, the QV of the assembled LHH and Newhall genomes reached 46.64 and 38.89, respectively, thus reflecting high accuracy.
7. Figure 3 E, F images – Some chromosomes have white regions within them. What these white regions indicate?
[Response]: We sincerely appreciate the questions raised about our research. The white areas in Figures 3E and 3F are due to weaker Hi-C contact signals in these regions compared to others. These regions, observed to be located near telomeres, may have lower Hi-C data coverage. However, upon inspection, these regions had already achieved chromosome-level assembly in the initial contig assembly. Therefore, Hi-C contact information was not used for the assembly of these regions, and their accuracy is not affected.
8. Table S1-S3, S5, S6, S8 Citrus sineses – spelling mistakes

	[Response]: Thank you very much for pointing out this error. We have corrected the issue in the relevant sections and thoroughly reviewed the manuscript content. We apologize for any inconvenience this may have caused.<br>9. Figure 4 – What is the C. sinensis ref? haven't mentioned which genome used as the reference.<br>[Response]: We sincerely appreciate you pointing out the missing information. In Figure 4, "C. sinensis ref" refers to the sweet orange reference genome obtained from the NCBI database, with the corresponding RefSeq ID GCF_022201045.2. We have added this information to the revised manuscript and highlighted it accordingly. We deeply apologize for the oversight.<br>10. This manuscript does not have separate sections clearly mentioned as the background, Methodology, and Results. Please adhere to the journal guidelines. The methods and results have been combined under the sub topics of "Genome survey, Genome assembly and assessment, genome annotation).<br>[Response]: Thank you sincerely for providing valuable suggestions. In accordance with your suggestions, we have made the necessary adjustments to the structure of the manuscript and sincerely apologize for the structural issues present in the article.<br>11. The authors have selected the gene models coming from the final integration of different gene annotation methods (Table S7). However, it is not clear how the integration was done. Was there a specific software or a tool or was it done manually? This information was not found in a methodology section. Please explain. It's better to have these materials in the methodology section.<br>[Response]: We sincerely appreciate you pointing out the missing information in our manuscript. In this study, EVidenceModeler (v1.1.1) [4] was used to merge multiple versions of genome annotations. We have added this information to the Materials and Methods section of the manuscript and highlighted it accordingly. We deeply apologize for the omission of this detail.<br>12. Lines 301-302 What's the basis of using other non-citrus genomes for this analysis? The comparison among citrus genomes makes sense. It would be good to know what do you expect by comparing citrus genomes with other genomes. Also, on which basis did you select those genome assemblies? Are they high quality? Because all the subsequent analysis such as gene family clustering, expansion and contraction etc. depend upon the accuracy and the completeness of the assemblies. Therefore, it's always safe and good to use high quality, highly contiguous, complete genomes for any comparative analysis to get accurate results.<br>[Response]: Thank you for your valuable questions regarding our work. The inclusion of multiple non-Citrus species aims to use the known divergence times between these species to provide a more accurate estimation of the divergence times among the Citrus species of interest. Additionally, this approach enhances the accuracy and reliability of gene family clustering and the analysis of gene family expansion and contraction.<br>13. Line 316, 318 P. trifoliata should be Italy sized.<br>[Response]: We sincerely appreciate you pointing out this issue. We have made the necessary corrections at the relevant locations in the manuscript and have reviewed the entire document. The modified sections have been highlighted. We deeply apologize for any inconvenience caused.<br>14. A clear and comprehensive description of the methodology for CAFÉ analysis and the WGD analysis should be provided. Which tools you used and the steps you followed would be important in enabling the replication of work for others.<br>[Response]: We sincerely appreciate your valuable suggestions regarding our work. Based on your recommendations, we have made the necessary additions. In this study, the Computational Analysis of gene Family Evolution (CAFÉ) software (v4.2) [5] was used to estimate the number of gene family members in ancestral branches through the birth-death (λ) model, based on phylogenetic trees with divergence times and gene family clustering results. This approach allowed us to predict the contraction and expansion of gene families relative to ancestors in each species. The criteria for defining significant expansion or contraction in this study are that both family-wide P-values and Viterbi P-values are less than 0.05. Additionally, the WGD (v1.1.1) [6] software was used for analyzing whole-genome duplication (WGD) events.<br>15. Line 325 - Ancient polyploidization event which was common to all eudicots is a WGT event. All studies for genome assemblies of common citrus (Wu et al 2014 – C. clementina, Feng 2021 and Xu 2013 – C. sinensis), other Australian citrus genomes (Nakandala, 2024) and related species such as P. trifoliata (Peng 2021) revealed only one common, ancient WGT event. So, it would be good if you can make sure the

results are correct.
[Response]: Thank you sincerely for providing valuable suggestions. Based on your suggestion, we have re-examined the whole-genome duplication (WGD) events in the two sweet orange genomes. Our analysis revealed that the first peak was caused by repetitive sequences in sweet orange rather than a WGD peak. This situation has also been observed in previous studies of Poncirus trifoliata [7]. Therefore, consistent with earlier reports, our results also support that the sweet orange genome has undergone only one whole-genome triplication (WGT) event (Fig. R2). We sincerely appreciate you pointing out this issue. We have made the necessary revisions in the manuscript, highlighted the changes, and apologize for any inconvenience caused.

Figure R2: The distribution of Ks values illustrates WGT events in the evolution of the C. sinensis LHH and Newhall genomes (https://cbi.gxu.edu.cn/zwzhou/xdxu/Figures/Figure_R2.pdf).

16. The figure 5B indicates the Ks plots for two species. Was it based on orthologous genes between the two genomes? Usually, the polyploidization events for a particular genome is based on the paralogous gene pairs of that particular species (self-genome comparison). All the other previous studies have revealed species-specific WGD events using Ks plots of paralogous gene pairs of that particular species. The orthologous ks peaks for two species might give information about the divergence events between those two species, but not the WGD events of a particular species.
[Response]: Thank you sincerely for providing valuable suggestions. We sincerely appreciate you pointing out this issue. Based on your suggestion, we have removed the Ks plots of orthologous gene pairs that are unrelated to WGT events in Figure 5 (Fig. R2). We apologize for any inconvenience this may have caused.

Figure R2: The distribution of Ks values illustrates WGT events in the evolution of the C. sinensis LHH and Newhall genomes (https://cbi.gxu.edu.cn/zwzhou/xdxu/Figures/Figure_R2.pdf).

17. Lines 329-331 – Do you have any explanation for the implications of the LTR insertions in these genomes? What is the purpose of finding the LTR element insertions? For ex: does it have a relationship with genome size expansion of these genomes?
[Response]: Thank you sincerely for providing valuable suggestions. In response to your suggestions, we have provided additional explanations for this section of the manuscript. The identification of LTR insertions aims to explore the distribution landscape and evolutionary dynamics of LTRs in the LHH and Newhall genomes. In the three C. sinensis genomes, a higher density of recent LTR-RT insertions was observed compared to its closely related species, P. trifoliata. This increased density may have contributed to the larger genome size of C. sinensis relative to P. trifoliata [8].
18. Line 354 – The authors have not mentioned how the collinearity blocks were detected. There are specific tools like MCScanX to detect the collinear genes. Did you use such a tool? or was it based on Mummer alignments? Mummer does the alignment between two genomes and other types of tools are required to detect the synteny (ex: syri) or collinearity (MCScanX). Please elaborate how you performed the collinearity analysis. Ex: MCSCanX based collinearity requires protein fasta files for the detection of collinear genes, but not the genome assembly files. Also, collinearity is a special type of synteny which defines the same genes in the same order in the homologous chromosomes of two genomes.
[Response]: Thank you sincerely for providing valuable suggestions. Thank you very much for pointing out the missing information. In this study, we used GenomeSyn [9] for genome synteny analysis. This tool is based on the alignment results of the LHH and Newhall genomes using Mummer (v4.0.0beta2) [10] to construct and visualize syntenic regions between the two genomes. Following your suggestion, we have added the synteny analysis based on the gene sequences of LHH and Newhall (Fig. R3). This analysis was performed using MCScan-Python (https://github.com/tanghaibao/jcvi/wiki/MCscan-(Python-version)).

Powered by Editorial Manager® and ProduXion Manager® from Aries Systems Corporation

Figure R3: Synteny analysis between the LHH and Newhall genomes (https://cbi.gxu.edu.cn/zwzhou/xdxu/Figures/Figure_R3.pdf).

19. If the homologous gene identification was based on BLASTp, which e value was used?
[Response]: Thank you very much for pointing out the missing information. In the identification of homologous genes based on BLASTp [11], we used an e-value of 1e-5. We have updated the manuscript to include this information and sincerely apologize for the omission.
20. Figure 6B and 6C – It's not clear how these two images present the SVs between the two genomes. Do these bar graphs represent the number (Figure 6A) and the percentage (Figure 6C) of SVs of the two genomes or in one genome?
[Response]: Thank you very much for your valuable question. Figures 6A and 6C illustrate the structural variations between the LHH and Newhall T2T genomes. Figure 6A presents the types and counts of structural variations between homologous chromosomes of the LHH and Newhall T2T genomes. Figure 6B shows the percentage of structural variations located at different positions on homologous chromosomes, relative to the total number of structural variations between these chromosomes in the LHH and Newhall T2T genomes.
21. Line 422 – What are the differences in between the two varieties in terms of flesh color and photosynthetic efficiency?
[Response]: Thank you for your valuable questions regarding our research. In terms of flesh color, under the same cultivation conditions, the 'Longhuihong' fruit exhibits a distinct red color in the juice cells (indicative of anthocyanin accumulation), while 'Newhall' juice cells are a standard orange-yellow. Regarding photosynthetic rates, the parameters for 'Longhuihong' spring shoot leaves are significantly higher compared to those of 'Newhall', with increases of 33.4% in net photosynthetic rate, 55.6% in stomatal conductance, 20.9% in intercellular CO2 concentration, and 33.1% in transpiration rate. Analysis of variance (ANOVA) indicates that the differences in photosynthetic parameters between the two varieties are statistically significant [12].
22. Don't you have any phenotypic or expression data for these two varieties?
[Response]: Thank you sincerely for providing valuable suggestions. Based on your suggestion, we have added the phenotypic information for the two varieties. In terms of photosynthetic rates, the parameters for 'Longhuihong' spring shoot leaves are significantly higher compared to 'Newhall', with increases of 33.4% in net photosynthetic rate, 55.6% in stomatal conductance, 20.9% in intercellular CO2 concentration, and 33.1% in transpiration rate. Analysis of variance (ANOVA) indicates that the differences in these photosynthetic parameters between the two varieties are statistically significant [12].
In this study, RNA sequencing was performed on pooled tissues from both varieties to obtain gene expression information. However, since the primary focus of this research is on the assembly and comparative genomics analysis of the two sweet orange genomes, our RNA-seq data were only utilized for gene annotation based on transcript evidence.
23. The raw sequence data could be found in the given NCBI bioproject. Please give the accession numbers of the deposited genome assemblies and annotations in NCBI or other databases. Only the bioproject number will not be sufficient for the others to get access to this data in the future.
[Response]: Thank you sincerely for providing valuable suggestions. In response to your suggestion, we have updated the data availability section of the manuscript with detailed information on the publicly available data. The PacBio HiFi, Hi-C, ONT UL, Illumina PE, and RNA-seq sequencing data for this project can be accessed through NCBI BioProject PRJNA1122682, with corresponding numbers SRR29362821 to SRR29362830 (Table R1). The LHH and Newhall genomes have been deposited in DDBJ/ENA/GenBank under accession numbers JBFBVJ000000000 and JBFBVK000000000, respectively.

Table R1. NCBI accession numbers for LHH and Newhall genome sequencing data

Accession	Title
SRR29362821	Newhall RNA-Seq
SRR29362822	LHH RNA-Seq
SRR29362823	Newhall Illumina PE
SRR29362824	Newhall Hi-C
SRR29362825	Newhall ONT UL

Powered by Editorial Manager® and ProduXion Manager® from Aries Systems Corporation

SRR29362826Newhall PacBio HiFi
SRR29362827LHH Illumina PE
SRR29362828LHH Hi-C
SRR29362829LHH ONT UL
SRR29362830LHH PacBio HiFi

Reviewer #2:
This is a study to construct the entire genome of two sweet orange mutant strains. This research is important for understanding the cold-resistance of sweet oranges, which is the ultimate goal. The objectives and approach of the study are appropriate and rationale. Genome assembly follows standard procedures, and the analysis of SV and functional annotation were also performed according to standard procedures. The BUSCO score suggests that the quality of the assembled genome is high, and it also indicates that the full-length sequence retains telomere structure at both ends of the chromosome.
While the results presented are generally understandable, some descriptions are unclear, some seem erroneous, and some require confirmation. The following is a list of such cases. Please check them and correct them appropriately.

1. Although the amounts of reads used in the analysis of the two sweet orange mutant lines tested are almost the same, discrepancies are observed in the assembly size (Table 2; 361.41 Mb/370.23 Mb) and estimated gene number (31,456/30,021). This study identified 2,886 structural variants between these two genomes; however, I feel that number is quite large as they are mutants.
Since many studies take a similar approach, I highly encourage authors to also discuss possible problems with the genome assembly process.
[Response]: Thank you for your valuable suggestions regarding our research. Based on your advice, we have reviewed the genome assembly and confirmed that the variations observed were not due to assembly errors but rather due to sequence differences between the haploid genomes. Consequently, we have included genome assemblies specific to the LHH and Newhall haplotypes (Fig. R1) and identified haplotype-specific structural variations. Our analysis detected a total of 164 haplotype-specific structural variations. This information has been incorporated into the manuscript.

Figure R1: Synteny between the LHH and Newhall diploid genomes (https://cbi.gxu.edu.cn/zwzhou/xdxu/Figures/Figure_R1.pdf).

2. Figure 3. E and F: Why are the estimated positions of CENs different in Chr 01 and 08 between these two assemblies? Any possible causes, if any, should be indicated in the results and discussion.
[Response]: Thank you for your valuable suggestions regarding our research. In response to the issues you pointed out, we used the recently published CentIER (v3.0) to re-evaluate the centromere regions. This tool has been reported to outperform similar software by more than 20% in various accuracy prediction metrics [13]. Through this re-evaluation, centromeres on multiple chromosomes, including Chr 01 and Chr 08, have been corrected (Fig. R4). We have updated the relevant information in Figure 3E, F, and the supplementary materials of the manuscript.

Figure R4: The distribution landscape of centromeres, telomeres, LAI, HiFi reads coverage, and ONT reads coverage in the LHH genome (A) and Newhall genome (B) (https://cbi.gxu.edu.cn/zwzhou/xdxu/Figures/Figure_R4.pdf).

3. Also, in Figure 3F, almost the entire length of the assembled chromosome appears to be covered by the ONT reads, while in Figure 3E, only some regions appear to be covered by the ONT reads. This is not consistent with the description in text L226-228.
[Response]: Thank you for pointing out this issue. The coverage depicted in Figure E does not indicate that ONT reads are specifically targeting localized regions. Instead, both HiFi and ONT reads cover most of the regions, with similar coverage levels, leading to overlap. Figure F illustrates that the higher coverage observed for ONT reads is consistent with the sequencing data statistics: the ONT read coverage for the

LHH genome is 51×, whereas for the Newhall genome, it is 61×.
4. Figure 6 is too large; I encourage splitting A, B, and C into single figures and providing B and C as supplements.
[Response]: Thank you for your valuable suggestions regarding our research. We have revised Figure 6 as requested and moved panels 6B and 6C to the supplementary figures section.
5. Although the similarities between the two assembled orange genomes are shown in Figure 6A, it is difficult to understand the correspondence between these two genomes. Please also add a Matrix plot (mummer plot) of all chromosomes.
[Response]: Thank you for your suggestions on our article. Following your advice, we have added a matrix plot (MUMmer plot) showing the genome comparison results to the supplementary figures (Fig. R5).

Figure R5: Dot matrix comparison of the C. sinensis LHH and Newhall genomes (https://cbi.gxu.edu.cn/zwzhou/xdxu/Figures/Figure_R5.pdf). The x-axis represents the LHH genome coordinates, and the y-axis represents the Newhall genome coordinates.

6. Figure 6A What do the pale green triangles at the chromosome ends of Chr02, Chr04, Chr07, Chr08, and Chr09 mean? Also, how were the SNPs shown here called?
[Response]: Thank you for your suggestions on our research. The triangular markers at the chromosome ends indicate the presence of telomeres at these locations. The SNP calling method used in this study is as follows: Minimap2 (v2.26-r1175) [14] was employed to align the PacBio HiFi reads of the LHH genome to the Newhall genome. Subsequently, DeepVariant (v1.6.1) [15] was used for SNP detection from the alignment results. Heterozygous SNPs identified in the SNP calling results were filtered out, resulting in the final dataset used for presentation. We have supplemented this information in the Materials and Methods section of the manuscript and sincerely apologize for the omission.
7. L72: This sentence says that about 80% of sweet oranges are derived from somatic mutations, but it is unclear on what basis. Does this 80% represent the percentage of sports and nucellar seedlings? If the remaining ca. 20% are neither sports nor nucellar seedlings, then they are sweet orange hybrids, not sweet oranges. This needs to be corrected.
[Response]: Thank you for your excellent suggestion. Currently, bud mutation selection is the primary source of cultivated sweet orange varieties. It is estimated that about 80% of sweet orange varieties originate from bud mutation selection based on somatic variation. Apart from bud mutation selection, the remaining 20% of sweet orange varieties are primarily developed through radiation and chemical mutagenesis methods.
8. L136: What do you mean for 'mid-to-high altitude'? Is this a proper noun?
[Response]: Thank you very much for raising questions about our research. We intended to convey the elevation of the experimental field. We have revised the manuscript to specify the exact elevation of the experimental field as 750 meters and sincerely apologize for any confusion caused by the previous description.
9. L103 What do you mean 'perfume lemon'?
[Response]: Thank you for your questions regarding our research. The types of cultivated citrus varieties we typically refer to include sweet oranges, mandarins, grapefruits, lemons, and their hybrids. ‘Perfume lemon’ refers to a variety of lemon known for its distinctive fragrance.
10. L145 Reference is required for this protocol.
[Response]: Thank you for your valuable suggestions. In response to your comments, we have reviewed and corrected the relevant section of the manuscript, and we have added the appropriate references. The modified content has been highlighted for clarity. We sincerely apologize for the incomplete information in this section.
11. L157 Insert 'DNA' before 'concentration'.
[Response]: Thank you for your valuable suggestions on our article. Based on your recommendations, we have made the necessary revisions in the corresponding sections of the manuscript.
12. L178 I suppose this 'Fig. 1B, D' would be 'Fig. 2B, D'.
[Response]: Thank you very much for pointing out this issue with our manuscript. It should indeed be Fig. 2B, D. We have corrected and highlighted the changes in the relevant sections of the manuscript and have re-checked the document. We apologize for the labeling error.

13. L201 Is 'Fig. 2C' 'Fig. 3C'?
[Response]: Thank you very much for pointing out this issue in our manuscript. It should indeed be Fig. 3C. We have made the necessary corrections and highlighted the changes in the relevant sections of the manuscript. We have also carefully re-checked the document and apologize for the labeling error.
14. L279 Cite reference for those 'closely related species genomes'.
[Response]: Thank you for pointing out the missing information in our manuscript. Following your suggestion, we have added the references to the manuscript and apologize for the oversight regarding this information.
15. L289 714 and 3,920 rRNA, these numbers show a large discrepancy. Confirm them.
[Response]: Thank you for your valuable suggestions regarding our research. Following your advice, we have reviewed the identified rRNA quantity and can confirm that it is indeed 3,920.
16. L302 Please describe the names of those 13 species, respectively in parenthesis.
[Response]: Thank you for pointing out the missing information in our manuscript. Following your suggestion, we have added the full names of the 13 species to the manuscript and apologize for the oversight regarding this information.
17. L319 Did you use known calibration point for the divergence time estimation?
[Response]: Thank you for the valuable questions you have raised about our research. In our study of divergence events, we obtained fossil dates from the TimeTree website (http://www.timetree.org/): 179.9-204.9 Mya for A. trichopoda vs. C. maxima, 0.44-0.48 Mya for C. maxima vs. C. sinensis Ref, 1.47-5.73 Mya for C. maxima vs. C. clementina, and 90-100.5 Mya for C. maxima vs. A. thaliana. We have now included this information and the specific analysis methods in the manuscript, highlighting these additions. We apologize for the oversight regarding this information.
18. L329-331 This sentence looks strange. Navel orange is a mutant line of sweet orange, and sweet orange has been recognized to emerge around 2,000 years ago, not 5 Mya. Furthermore, Newhall is the selection of navel orange discovered in 1912 in UC Riverside. Revise this sentence sounds coincide with natural history of sweet orange and its mutant selections.
[Response]: Thank you for highlighting this issue with our research. We have removed the inappropriate expressions from our manuscript and sincerely apologize for these inaccuracies.
19. Figure 5B Describe the unit of Density (vert axis).
[Response]: Thank you for your suggestions regarding our research. The y-axis unit of the density plot does not have a specific physical unit because density is a relative measure. It represents the density value of the data at that point. Typically, it does not have a direct unit but can be understood as ‘the probability per unit interval.’
References
1. Cheng H, Concepcion GT, Feng X, Zhang H, Li H. Haplotype-resolved de novo assembly using phased assembly graphs with hifiasm. Nat Methods. 2021; doi: 10.1038/s41592-020-01056-5.
2. Xie L, Gong X, Yang K, Huang Y, Zhang S, Shen L, et al.. Technology-enabled great leap in deciphering plant genomes. Nat Plants. Nature Publishing Group; 2024; doi: 10.1038/s41477-024-01655-6.
3. Rhie A, Walenz BP, Koren S, Phillippy AM. Merqury: reference-free quality, completeness, and phasing assessment for genome assemblies. Genome Biol. 2020; doi: 10.1186/s13059-020-02134-9.
4. Haas BJ, Salzberg SL, Zhu W, Pertea M, Allen JE, Orvis J, et al.. Automated eukaryotic gene structure annotation using EVidenceModeler and the Program to Assemble Spliced Alignments. Genome Biol. 2008; doi: 10.1186/gb-2008-9-1-r7.
5. Han MV, Thomas GWC, Lugo-Martinez J, Hahn MW. Estimating gene gain and loss rates in the presence of error in genome assembly and annotation using CAFE 3. Mol Biol Evol. 2013; doi: 10.1093/molbev/mst100.
6. Zwaenepoel A, Van de Peer Y. wgd-simple command line tools for the analysis of ancient whole-genome duplications. Bioinforma Oxf Engl. 2019; doi: 10.1093/bioinformatics/bty915.
7. Peng Z, Bredeson JV, Wu GA, Shu S, Rawat N, Du D, et al.. A chromosome-scale reference genome of trifoliate orange (Poncirus trifoliata) provides insights into disease resistance, cold tolerance and genome evolution in Citrus. Plant J. 2020; doi: 10.1111/tpj.14993.
8. Zhang T, Qiao Q, Novikova PY, Wang Q, Yue J, Guan Y, et al.. Genome of Crucihimalaya himalaica, a close relative of Arabidopsis, shows ecological adaptation

Powered by Editorial Manager® and ProduXion Manager® from Aries Systems Corporation

	to high altitude. Proc Natl Acad Sci U S A. 2019; doi: 10.1073/pnas.1817580116.<br>9. Zhou Z-W, Yu Z-G, Huang X-M, Liu J-S, Guo Y-X, Chen L-L, et al.. GenomeSyn: a bioinformatics tool for visualizing genome synteny and structural variations. J Genet Genomics Yi Chuan Xue Bao. 2022; doi: 10.1016/j.jgg.2022.03.013.<br>10. Marçais G, Delcher AL, Phillippy AM, Coston R, Salzberg SL, Zimin A. MUMmer4: A fast and versatile genome alignment system. PLoS Comput Biol. 2018; doi: 10.1371/journal.pcbi.1005944.<br>11. Cock PJA, Chilton JM, Grüning B, Johnson JE, Soranzo N. NCBI BLAST+ integrated into Galaxy. GigaScience. 2015; doi: 10.1186/s13742-015-0080-7.<br>12. Yuan, Gaopeng, Chun, changpin, Peng, Liangzhi, Huang, Zongjun, Huang, Tongqi, Yang, Huidong, et al.. A Comparative Analysis of 'Newhall' Navel Orange and Its Bud Sport 'Longhuihong.' Fruit Tree J. 2017; doi: 10.13925/j.cnki.gsxb.20170096.<br>13. Xu D, Yang J, Wen H, Feng W, Zhang X, Hui X, et al.. CentIER: accurate centromere identification for plant genome. Plant Commun. Elsevier; 2024; doi: 10.1016/j.xplc.2024.101046.<br>14. Li H. Minimap2: pairwise alignment for nucleotide sequences. Bioinforma Oxf Engl. 2018; doi: 10.1093/bioinformatics/bty191.<br>15. Poplin R, Chang P-C, Alexander D, Schwartz S, Colthurst T, Ku A, et al.. A universal SNP and small-indel variant caller using deep neural networks. Nat Biotechnol. 2018; doi: 10.1038/nbt.4235.
**Additional Information:**	
**Question**	**Response**
Are you submitting this manuscript to a special series or article collection?	No
**Experimental design and statistics**<br><br>Full details of the experimental design and statistical methods used should be given in the Methods section, as detailed in our Minimum Standards Reporting Checklist. Information essential to interpreting the data presented should be made available in the figure legends.<br><br>Have you included all the information requested in your manuscript?	Yes
**Resources**<br><br>A description of all resources used, including antibodies, cell lines, animals and software tools, with enough information to allow them to be uniquely identified, should be included in the Methods section. Authors are strongly encouraged to cite Research Resource Identifiers (RRIDs) for antibodies, model organisms and tools, where possible.<br><br>Have you included the information	Yes

requested as detailed in our Minimum Standards Reporting Checklist?	
**Availability of data and materials**<br><br>All datasets and code on which the conclusions of the paper rely must be either included in your submission or deposited in publicly available repositories (where available and ethically appropriate), referencing such data using a unique identifier in the references and in the “Availability of Data and Materials” section of your manuscript.<br><br>Have you have met the above requirement as detailed in our Minimum Standards Reporting Checklist?	Yes

Manuscript

Click here to access/download;Manuscript;Manuscript_Oct 9.docx

Construction and analysis of telomere-to-telomere genomes for two sweet oranges: Longhuihong and Newhall (*Citrus sinensis*)

Lin Hong[1,#,*], Xin-Dong Xu[2,3,#], Lei Yang[1,#], Min Wang[1], Shuang Li[1], Haijian Yang[1], Si-Ying Ye[2,3], Ling-Ling Chen[3], Jia-Ming Song[2,3]*.

[1]Fruit Tree Research Institute, Chongqing Academy of Agricultural Sciences, Chongqing 401329, China

[2]Integrative Science Center of Germplasm Creation in Western China (CHONGQING) Science City and Southwest University, College of Agronomy and Biotechnology, Southwest University, Chongqing 400715, China

[3]State Key Laboratory for Conservation and Utilization of Subtropical Agro-bioresources, College of Life Science and Technology, Guangxi University, Nanning 530004, China

E-mail:

loquatvalue@163.com (L.H.);

1546509060@qq.com (X.-D.X.);

leir8512@126.com (L.Y.);

wm950918@126.com (M.W.);

sclishuang61@163.com (S.L.);

yanghaijian126@126.com (H.Y.);

siyingyesdfmu@163.com (S.-Y.Y.);

llchen@gxu.edu.cn (L.-L.C.);

jmsong@swu.edu.cn (J.-M.S.);

loquatvalue@163.com (L.H.)

[#]These authors contributed equally: Lin Hong, Xin-Dong Xu, Lei Yang

*Correspondence: Lin Hong (loquatvalue@163.com), Jia-Ming Song (jmsong@swu.edu.cn)

Lin Hong [0000-0003-2268-1225]; Xin-Dong Xu [0009-0000-8610-7850]; Jia-Ming Song [0000-0002-6636-4152]

Abstract

Background: Sweet orange (*Citrus sinensis* Osbeck) is a fruit crop of high nutritional value that is widely consumed around the world. However, its susceptibility to low-temperature stress limits its cultivation and production in regions prone to frost damage, severely impacting the sustainable development of the sweet orange industry. Therefore, developing cold-resistant sweet orange varieties is of great necessity. Traditional hybrid breeding methods are not feasible due to the polyembryonic phenomenon in sweet oranges, necessitating the enhancement of its germplasm through molecular breeding. High-quality reference genomes are valuable for studying crop resistance to biotic and abiotic stresses. However, the lack of genomic resources for cold-resistant sweet orange varieties has hindered the progress in developing such varieties and researching their molecular mechanisms of cold resistance.

Findings: This study integrated PacBio HiFi, ONT, Hi-C, and Illumina sequencing data to assemble telomere-to-telomere (T2T) reference genomes for the cold-resistant sweet orange mutant 'Longhuihong' (*Citrus sinensis* [L.] Osb. cv. LHH) and its wild-type counterpart 'Newhall' (*Citrus sinensis* [L.] Osb. cv. Newhall). Comprehensive evaluations based on multiple criteria revealed that both genomes exhibit high continuity, completeness, and accuracy. The genome sizes were 340.28 Mb and 346.33 Mb, with contig N50 of 39.31 Mb and 36.77 Mb, respectively. In total, 31,456 and 30,021 gene models were annotated in the respective genomes. Leveraging these assembled genomes, comparative genomics analyses were performed, elucidating the evolutionary history of the sweet orange genome. Moreover, the study identified 2,886 structural variants (SVs) between the two genomes, with several SVs located in the upstream, downstream, or intronic regions of homologous genes known to be associated with cold resistance.

Conclusions: The study *de novo* assembled two T2T reference genomes of sweet orange varieties exhibiting different levels of cold tolerance. These genomes serve as valuable foundational resources for genomic research and molecular breeding aimed at enhancing cold tolerance in sweet oranges. Additionally, they expand the existing repository of reference genomes and sequencing data resources for *Citrus sinensis*. Moreover, these genomes provide a critical data foundation for comparative genomics analyses across different plant species.

Keywords: sweet orange; *Citrus sinensis*; Longhuihong; Newhall; telomere-to-telomere genome; cold tolerance

Introduction

The sweet orange (*Citrus sinensis* Osbeck; NCBI:txid2711), a member of the Rutaceae family and Citrus genus, is globally recognized as one of the most commercially valuable fruits due to its high content of bioactive compounds such as flavonoids, phenolic acids, alkaloids, carotenoids, and limonoids, which provide significant anti-inflammatory, anti-cancer, and antioxidant benefits. These properties make sweet oranges widely applicable in agriculture, food, and medicine [1,2]. Sweet oranges can be consumed fresh or juiced and are generally categorized into three types: blond oranges, navel oranges, and blood oranges [3]. Navel oranges, distinguished by a secondary fruitlet (resembling a navel) at the bottom of the fruit, have a high mutation rate that has facilitated their proliferation, with numerous cultivars being bred and disseminated worldwide [4]. To date, there are over 190 known varieties, with commonly cultivated types including ‘Washington’, ‘Newhall’, ‘Chislett’, ‘Powell’, ‘Lane Late’, ‘Barnfield’, and ‘Cara Cara’ [4,5]. Somatic mutation-based bud sport breeding has become a vital method for developing new fruit tree varieties. It is estimated that around 80% of sweet

orange cultivars originate from somatic mutations, with sweet oranges being cultivated in 114 countries globally. Moreover, the apomictic nature of sweet oranges allows for strict clonal propagation under natural conditions, making them an ideal species for studying somatic mutations [6,7].

The genome is considered a foundational element in biological research. In 2000, the first plant genome, the *Arabidopsis thaliana* genome, was published by scientists [8]. Over the past two decades, with rapid advancements in sequencing technology, assembly algorithms, genetics, and bioinformatics [9], nearly 900 plant species' genomes have been published to date (based on data from NCBI and GWH). In 2012, the first genome of the sweet orange was sequenced. To reduce assembly complexity, double haploid sequencing was utilized by researchers, resulting in the successful mapping of 87% of the sweet orange genome. The total assembly size was determined to be 320 Mb, with a contig N50 of 49.89 Kb, a scaffold N50 of 1.69 Mb, and 29,445 protein-coding genes were identified [10]. In 2014, the genome sequences of seven citrus species, including clementine, mandarin, pummelo, sweet orange, and sour orange, were completed. Higher-quality genomes for the Citrus genus were produced, significantly improving upon the previously published sweet orange genome (contig N50 = 119 Kb, scaffold N50 = 6.8 Mb). Evolution and domestication of the Citrus genus were explored, indicating that the cultivated pummelo originated from the ancestral species *C. maxima*, while the cultivated mandarin was derived from *C. reticulata* with introgressions from *C. maxima* [11]. In 2017, single-molecule sequencing technology was employed to complete the genomes of four representative citrus species, achieving a contig N50 of 2.2 Mb, which is over 18 times greater than previously reported citrus genomes. Comparative genomic analysis revealed that the citrus-specific genomic regions primarily consist of repeat sequences and genes of unknown function, with approximately one-fifth of the genes having known functions and being enriched in biological pathways related to resistance,

proteolysis, and pectin degradation [12]. In 2021, a bud sports population was combined with genomics strategies to improve the scope and accuracy of somatic mutation predictions. The genomes of a double haploid sweet orange and six diploids were first assembled, leaving an average of only three gaps per chromosome across the nine chromosomes [7]. In 2023, a genomic map of the Citrus subfamily was constructed, with de novo assembly of the genomes of 12 Citrus genus and closely related species. The Contig N50 of these genomes ranged from 1.6 to 16.8 Mb, with assembly sizes ranging from 217.8 to 419.1 Mb, thus covering over 90% of their estimated genome sizes. The number of annotated gene models in these genomes ranged from 22,907 to 31,413 [13]. In 2023, a complete and haplotype-resolved T2T genome of the lemon variety perfume lemon was assembled by researchers. The assembled genome is 633.0 Mb in size, with a contig N50 of 35.6 Mb and zero gaps, achieving a fully gap-free assembly. Further multi-omics analyses identified candidate genes associated with the biosynthesis of flavor compounds and resistance to Huanglongbing (HLB), providing a foundation for accelerating molecular breeding programs and the discovery of functional genes [14]. In 2024, a study has reported the T2T genome of the ‘Nei Xiu’, a bud mutation originating from Tarocco blood orange[15].

‘Longhuihong’ (LHH) is a bud mutation of the ‘Newhall’ navel orange, known for its enhanced cold tolerance. The low-temperature stress during winter stimulates the synthesis of anthocyanins in LHH fruits, leading to visibly red juice sacs (Fig. 1A). The leaves display distinct curling and prominent veins, making its variation characteristics highly noticeable (Fig. 1B). Variety trials have demonstrated that LHH possesses several agronomic traits superior to those of Newhall, indicating that it may harbor multiple advantageous genotypes with significant development potential. However, the lack of available genomic resources for LHH currently limits the exploration and utilization of the

genetic basis behind its desirable traits—such as cold tolerance, high photosynthetic efficiency, and high anthocyanin content. This poses a significant challenge to molecular breeding efforts for sweet oranges.

This study leveraged state-of-the-art sequencing technologies, including PacBio High Fidelity (HiFi), Oxford Nanopore Technologies Ultra Long (ONT UL), high-throughput chromosome conformation capture (Hi-C), and Illumina sequencing data, to report the T2T genome assembly of navel orange varieties, LHH and Newhall. These assemblies provide valuable genomic resources for pangenome studies, functional gene identification, and molecular breeding of sweet oranges. Furthermore, by utilizing the two T2T genomes, we elucidated the evolutionary history of the sweet orange genome and characterized the genomic variations between the two varieties. This research offers critical data support for future genomic studies and breeding programs aimed at enhancing cold tolerance in sweet oranges.

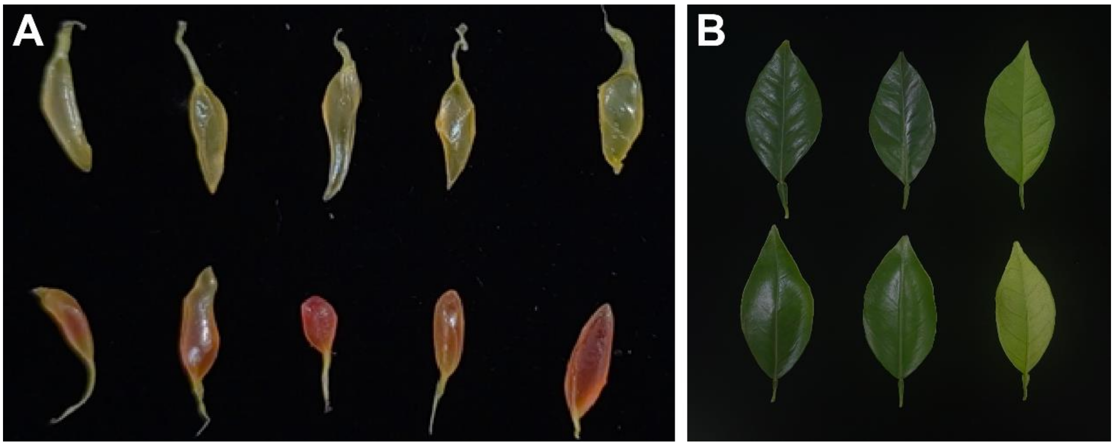

Figure 1: Morphological characteristics of *C. sinensis* LHH and Newhall. (A), Comparison of juice sac characteristics between navel orange cultivar Newhall (top) and LHH (bottom). (B), Comparison of leaf characteristics between navel orange cultivar LHH (top) and Newhall (bottom).

Materials and Methods

Sample collection

Samples of the navel orange varieties LHH and Newhall were collected from an experimental orchard specializing in sweet orange varieties in Fengming Town, Yunyang County, Chongqing, at an elevation of 750 meters. Fresh leaf samples were obtained from 4-year-old trees that had been grafted onto trifoliate orange rootstocks and planted at a density of 5 meters by 3 meters. These samples were then used for subsequent DNA extraction, library construction, and sequencing.

DNA isolation, library construction, and sequencing

Healthy, juvenile leaves are carefully selected and their surfaces are thoroughly rinsed with deionized water. After gently drying the leaves to eliminate surface moisture, we precisely cut the tissue into 50-100 mg fragments. These fragments are then placed into pre-prepared 2 mL cryovials and promptly frozen in liquid nitrogen for a duration of 3-4 hours. Following this, the samples are securely stored at -80° C for future DNA and RNA extraction. High-quality genomic DNA was extracted from the leaves of *C. sinensis* LHH and Newhall using the cetyl-trimethylammonium bromide (CTAB) method [16]. Qualified genomic DNA samples were then used for the construction of sequencing libraries. The size and quantity of library fragments were assessed using Qseq400 and Qubit, respectively, ensuring library quality. The qualified libraries were subsequently immobilized onto sequencing chips via bridge PCR methods. Illumina sequencing was eventually conducted, performing 150 bp paired-end sequencing on an Illumina sequencer. For PacBio Circular Consensus Sequencing (CCS), long-fragment library construction was carried out using genomic DNA extracted from leaf samples, following the manufacturer's instructions. The genomic DNA was then sheared into 15 kb fragments. Sequencing of the constructed library was executed on the PacBio Sequel II platform

(RRID:SCR_017990). Post-sequencing, low-quality reads and sequencing adapters were removed to obtain clean subreads. Regarding ONT UL sequencing, library preparation was conducted using the SQK-ULK001 kit, following the manufacturer's instructions. Libraries were purified and sequenced using a PromethION sequencer. The Hi-C experiment protocol is as follows: (1) Collect 1 gram of tender leaves, rinse them thoroughly in icy water, and gently blot them dry with absorbent paper. (2) Using scissors, carefully mince the sample tissue and place it into a 50 mL centrifuge tube. (3) Transfer the minced tissue into a 50 mL centrifuge tube containing 35 mL of NIB Buffer. (4) Introduce 35 μL of PMSF, 35 μL of β-mercaptoethanol, and 2 mL of 36% formaldehyde into the mixture. (5) Allow the mixture to react for 90 minutes on a vertical rotator. (6) Add 2.5 mL of 2M glycine and gently agitate the solution, either manually or on a shaker, for a minimum of 5 minutes to halt the reaction. (7) Filter out the excess liquid and rinse the sample thoroughly with sterile water until all foam disappears. (8) Blot the sample dry with absorbent paper, place it in a 50 mL centrifuge tube, and promptly freeze it in liquid nitrogen for future use. Upon passing quality checks, Illumina-based high-throughput sequencing was performed, generating 150 bp paired-end reads.

Genome survey

Before genome assembly, a 21-mer-based survey was conducted on the LHH and Newhall genomes to estimate genome size, heterozygosity, repeat content, and ploidy level, providing critical reference information for subsequent assembly efforts. Jellyfish (v2.3.1) [17] was employed to count the 21-mers from the Illumina paired-end sequencing data. Following this, GenomeScope (RRID:SCR_017014) (v2.0) [18] was used to analyze the 21-mer frequency distributions. Smudgeplot (v0.2.5) [18] was utilized to estimate genome ploidy.

Genome assembly and assessment

Based on the PacBio HiFi, ONT UL, and Hi-C sequencing data obtained from whole-genome sequencing, the LHH and Newhall navel orange genomes were de novo assembled. Hifiasm (v0.19) [19] was utilized for the initial assembly of the LHH and Newhall genomes, with ONT UL sequencing data integrated using the "–ul" parameter to enhance the assembly process. Subsequently, the initially assembled contigs were aligned to the NCBI NT database, the mitochondrial database, and the plastid database to filter out contaminants, mitochondrial sequences, and plastid sequences, resulting in clean contig sequences. Leveraging Hi-C contact signals, LACHESIS (RRID:SCR_017644) [20] was employed to group, order, and orient the contigs with the parameters: "CLUSTER_MIN_RE_SITES = 100; CLUSTER_MAX_LINK_DENSITY = 2; ORDER_MIN_N_RES_IN_TRUNK = 15; ORDER_MIN_N_RES_IN_SHREDS = 15." Subsequently, Juicebox (RRID:SCR_021172) [21] was used to manually inspect and adjust the scaffolding results from LACHESIS. TGS-Gapcloser (RRID:SCR_017633) (v1.2.1) [22] was utilized to close gaps in the anchored reference genome using ONT UL sequencing data. Ultimately, 8 and 7 gap-free chromosome assemblies were obtained for the LHH and Newhall sweet orange varieties, respectively. Potential telomeric repeat units within the genome were identified using TIDK. Subsequently, potential telomeric sequences were located with FindTelomeres (RRID:SCR_024403) based on these repeat units, enabling the acquisition of both telomeric positions and sequences. CentIER (v3.0) [23] was used to identify potential centromere regions. After completing the assembly of the two sweet orange genomes, various strategies were employed to verify their completeness and accuracy. The Benchmarking Universal Single-Copy Orthologs (BUSCO) (RRID:SCR_015008) [24] dataset, embryophyta_odb10, comprising 1614 genes, was mapped to the genomes. Minimap2 (RRID:SCR_018550) (v2.26-r1175) [25] was then used to map the HiFi and ONT UL reads to the genomes. LTR_retriever (RRID:SCR_017623) (v2.9.8) [26]

was employed to calculate the LTR Assembly Index (LAI) based on Long Terminal Repeat (LTR) annotations, to evaluate the assembly quality of repetitive sequences in both genomes [27]. Merqury (RRID:SCR_022964) (v1.3) [28] was utilized to calculate the assembly consensus quality value (QV) for the two genomes.

Genome annotation

De novo prediction of repetitive sequences was performed using RepeatModeler (RRID:SCR_015027) (v2.0.1) [29], while LTR_retriever (v2.9.0) [26] was utilized for de novo prediction of LTRs. The results from both tools were merged with Repbase (RRID:SCR_021169) [30] and deduplicated to construct a comprehensive repeat library for the two navel orange genomes. This repeat library was then used by RepeatMasker (RRID:SCR_012954) (v4.1.2) [31] for genome-wide repeat annotation, resulting in masked versions of the genomes. Augustus (RRID:SCR_008417) (v3.1.0) [31] and SNAP (RRID:SCR_007936) [32] were used for *de novo* gene model predictions for the LHH and Newhall genomes; GeMoMa (RRID:SCR_017646) (v1.7) [33] was employed for gene model predictions based on homologous genes from closely related species. In this study, two strategies were used for transcript-based gene model predictions. The first strategy involved processing RNA-seq data with HISAT (RRID:SCR_015530) (v2.1.0) [34] and StringTie (RRID:SCR_016323) (v2.1.4) [35], followed by gene model prediction using GeneMarkS-T (RRID:SCR_017648) (v5.1) [36]. The second strategy used Trinity (RRID:SCR_013048) (v2.11) [37] for transcript assembly, and the assembled transcripts were then fed into PASA (RRID:SCR_014656) (v2.4.1) [38] for gene prediction. Finally, EVidenceModeler (RRID:SCR_014659) (v1.1.1) [39] was used to merge the gene model predictions from the three methods, and PASA (v2.4.1) [38] was used to adjust the merged annotations. HiCExplorer (RRID:SCR_022111) (v3.7.4) [40] was used to analyze A/B compartments from Hi-C

data. Additionally, the NR, eggNOG (RRID:SCR_002456) [41], GO, KEGG [42], TrEMBL [43], KOG, SWISS-PROT [43] and Pfam (RRID:SCR_004726) [44] databases were employed to perform functional annotation of these gene sequences. tRNAscan-SE (RRID:SCR_008637) (v1.3.1) [45] was utilized for the recognition of transfer RNA (tRNA), and Barrnap (RRID:SCR_015995) (v0.9) was used for the prediction of ribosomal RNA (rRNA). MicroRNAs (miRNAs), small nucleolar RNAs (snoRNAs), and small nuclear RNAs (snRNAs) were identified based on the Rfam (RRID:SCR_007891) (v14.5) [46] database using Infernal (RRID:SCR_011809) (v1.1) [47].

Comparative genomics and evolutionary analysis

MAFFT (RRID:SCR_011811) (v7.205) [48] was used to generate multiple sequence alignments (MSA) for these single-copy orthologs, and Gblocks (RRID:SCR_015945) (v0.91b) [49] was employed to remove highly variable regions from the MSA. The MSA of single-copy orthologs was then concatenated. IQ-TREE (RRID:SCR_017254) (v1.6.11) [50] was used to construct a maximum likelihood (ML) phylogenetic tree based on the MSA, applying the model “JTT+F+I+G4” and a bootstrap value of 1000. OrthoFinder (RRID:SCR_017118) (v2.4) [51] was used for gene family clustering. Computational Analysis of gene Family Evolution (CAFÉ: RRID:SCR_005983)) (v4.2) [52] was employed to estimate the number of gene family members in the ancestral branches using the birth-death (λ) model, based on the evolutionary tree with divergence times and gene family clustering results. This approach allowed for the prediction of gene family contraction and expansion relative to the ancestors. In this study, a gene family was considered to have undergone significant expansion or contraction if both the family-wide P values and Viterbi P values were less than 0.05. The estimation of divergence times was performed using the mcmctree module in PAML (RRID:SCR_014932) (v4.9i) [53].

Detection of variations

In this study, MUMandCo (v3.8) [54] was utilized to detect and classify structural variations from Mummer's alignment results. Based on alignment results from Mummer (RRID:SCR_018171) (v4.0.0rc1) [54], ANNOVAR (RRID:SCR_012821) [55] was used to annotate the detected structural variations relative to gene positions. GenomeSyn (v1.2.7) [55] was used to integrate and present these data and perform genome collinearity analysis. BLASTp (RRID:SCR_001010) (v2.5.0) [56] was used to map the collected cold tolerance-related genes from rice to the Newhall genome. Minimap2 (v2.26-r1175) [25] was used to align the PacBio HiFi reads of the LHH genome to the Newhall genome. Subsequently, DeepVariant (v1.6.1) [57] was employed to perform SNP detection from the alignment results. The heterozygous SNPs identified in the SNP calling results were then filtered out, resulting in the final dataset used for presentation.

Results

Genome sequencing

For the LHH and Newhall varieties, 28.04 Gb (~71×) and 26.07 Gb (~79×) of PacBio HiFi reads, 20.58 Gb (~51×) and 24.49 Gb (~61×) of ONT UL reads, 18.25 Gb (~43×) and 17.18 Gb (~46×) of Illumina PE reads, as well as 50.69 Gb (~136×) and 50.43 Gb (~146×) of Hi-C reads were obtained, respectively (Table 1).

Table 1: Statistics of the clean data of the *C. sinensis* LHH and Newhall genomes

	C. sinensis LHH			*C. sinensis* Newhall		
Type	Total data (Gb)	Sequence depth (×)	Average size (bp)	Total data (Gb)	Sequence depth (×)	Average size (bp)
PacBio	28.04	71	13,757	26.07	79	13,965

ONT	20.58	51	98,866	24.49	61	95,293
Hi-C	50.69	136	150	50.43	146	150
Illumina	18.25	43	150	17.18	46	150

Genome survey

The survey results revealed that the estimated genome sizes for LHH and Newhall were 310.75 Mb and 301.9 Mb, respectively, with heterozygosities of 2.79% and 2.67%. Both genomes exhibited high levels of heterozygosity and similar repeat content, 45.0% and 44.8%, respectively (Fig. 2A, C). Ploidy estimation results indicated that both LHH and Newhall genomes are heterozygous diploids, consistent with previously reported sweet orange genomes (Fig. 2B, D).

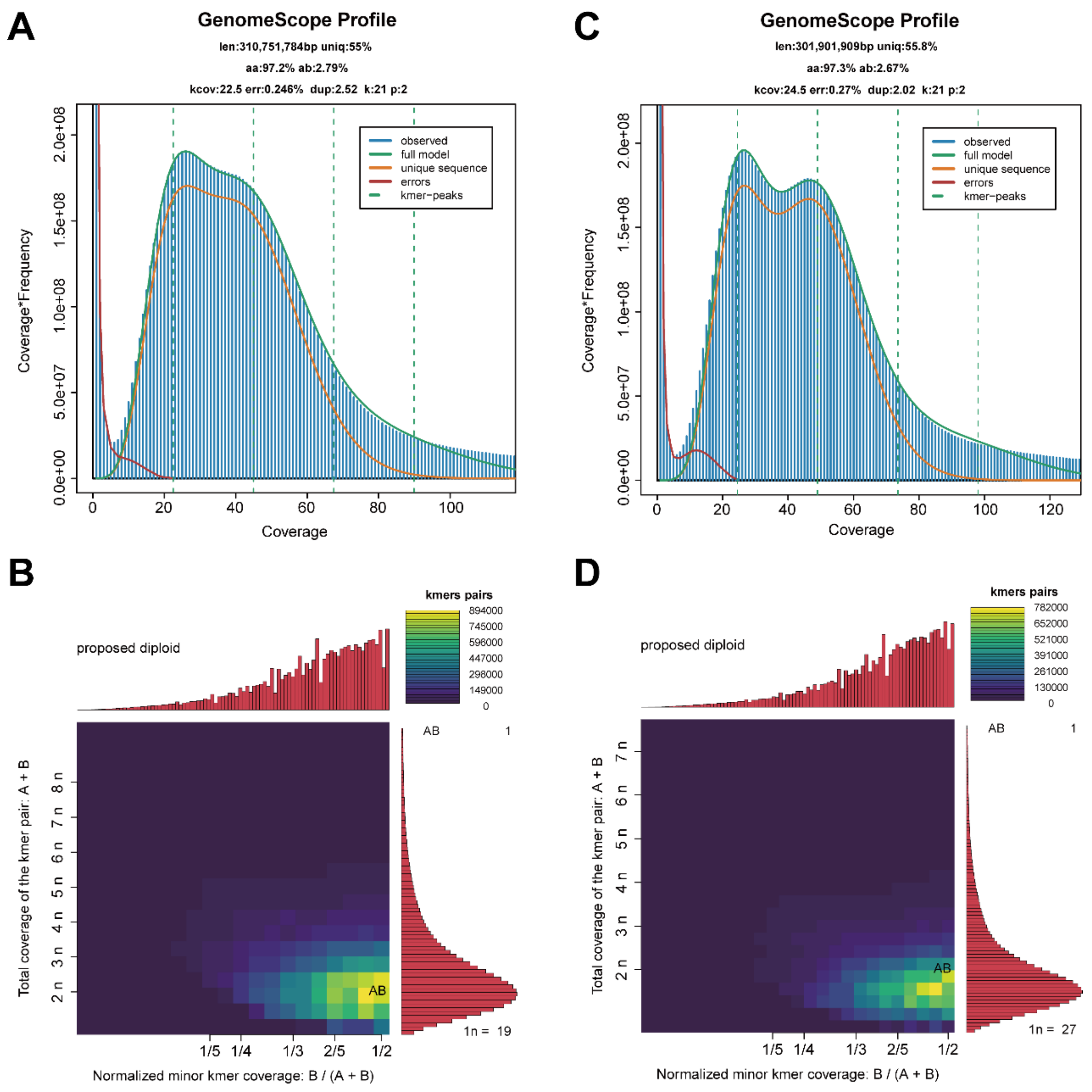

Figure 2: The genomic feature survey of *C. sinensis* LHH and Newhall genomes based on 21-mer. (A), 21-mer spectra for *C. sinensis* LHH. (B), Smudgeplots for *C. sinensis* LHH. (C), 21-mer spectra for *C. sinensis* Newhall. (D), Smudgeplots for *C. sinensis* Newhall.

Genome assembly and assessment

Initial assembly results indicated that the LHH and Newhall genomes contained 160 and 141 contigs, respectively, with total lengths of 372.71 Mb and 370.23 Mb, and contig N50 values of 36.91 Mb and 36.77 Mb. After filtering contaminants from the preliminary assembly, Hi-C data were used to anchor the clean contigs to the chromosome level for the LHH and Newhall genomes (Fig. 3C, D).

The statistics revealed that 338.55 Mb and 337.47 Mb of the clean contigs for LHH and Newhall, respectively, were anchored to 9 pseudochromosomes, with anchoring rates of 99.49% and 97.44% (Table S1). Telomeric sequences were detected at both ends of 7 chromosomes in each genome, while the remaining chromosomes exhibited telomeric sequences at one end. Centromeric sequences were identified on all chromosomes in both genomes, indicating that both assemblies achieved a T2T level [58] (Fig. 3E, F, Table S2, 3). The total lengths of the T2T genomes for LHH and Newhall were 340.28 Mb and 346.33 Mb, respectively, consistent with the genome sizes estimated from the genome surveys.

The BUSCO assessment results indicate that the completeness of the LHH and Newhall genomes is 99.07% and 99.19%, respectively (Fig. S1, Table S4). Additionally, the Core Eukaryotic Genes Mapping Approach (CEGMA) [59] was utilized for further evaluation, indicating completeness scores of 99.13% for the LHH genome and 99.56% for the Newhall genome. The mapping statistics showed that the HiFi reads had mapping rates of 99.11% for LHH and 99.58% for Newhall, with coverages of 99.98% and 99.99%, and average read depths of 71× and 68×, respectively. For the ONT UL reads, the mapping rates were 96.87% for LHH and 97.05% for Newhall, with coverages of 99.96% and 99.77%, and average read depths of 51× and 61×, respectively (Table S5). These results underscore the high completeness of both genome assemblies. The Hi-C contact matrices for both genomes displayed smooth and continuous Hi-C signals, confirming the correct order and orientation of the genome assemblies (Fig. 3C, D). Based on the LTR Assembly Index (LAI), the genome assembly quality assessment shows that the LAI values for the LHH and Newhall genomes are 20.39 and 20.09, respectively, both meeting the standards for gold reference genomes [27]. Further analysis of LAI across different chromosomal regions indicated that most regions of the T2T genomes exhibited high LAI, and the mapping results for HiFi and ONT UL reads demonstrated uniform coverage in these

areas (Fig. 3E, F). However, some regions showed relatively lower LAI, likely due to lower HiFi read coverage in those areas. Nevertheless, ONT UL reads successfully covered and filled these regions (Fig. 3E, F). Finally, the results of calculating the assembly consensus quality value (QV) for the LHH and Newhall genomes showed that the QV score for LHH was 46.64, while for Newhall it was 38.89. This indicates that the accuracies of the two genomes are 99.99% and 99.9%, respectively, demonstrating high accuracy [28].

Table 2: Assembly statistics of the *C. sinensis* LHH and Newhall genomes

	C. sinensis LHH		*C. sinensis* Newhall	
Items	Contig	Scaffold	Contig	Scaffold
Sequence Number	24	9	141	9
Assembly Size (Mb)	361.41	338.51	370.23	334.63
Longest SeqLen (Mb)	50.84	50.84	49.40	49.40
Average SeqLen (Mb)	15.06	37.61	2.62	37.18
N50 (Mb)	36.91	39.31	36.77	38.85

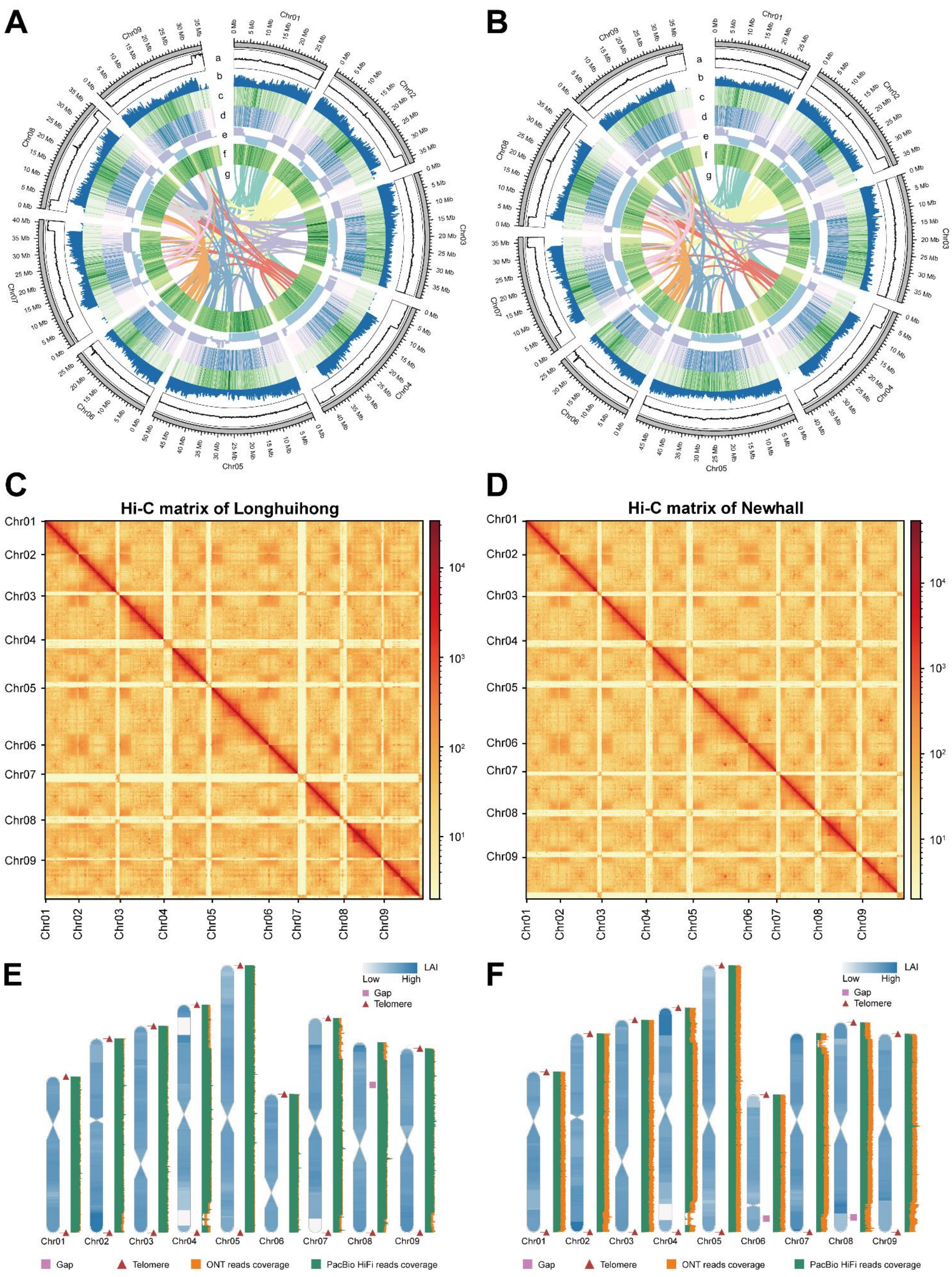

Figure 3: Assembly and assessment of the *C. sinensis* LHH and Newhall genomes. (A-B), Circos plot

of the genomic landscape of the *C. sinensis* LHH genome (A) and *C. sinensis* Newhall genome (B). The circos plots show, outermost to innermost, GC content (a), gene density (b), LTR/*Gypsy* density, LTR/*Copia* density (d), A/B compartment (e), DNA transposon density (f), and syntenic regions within the genome (g). (C-D), Hi-C contact matrix of LHH genome (C) and Newhall genome (D). (E-F), The distribution landscape of centromeres, telomeres, LAI, HiFi reads coverage, and ONT reads coverage in the LHH genome (E) and Newhall genome (F).

Genome annotation

The study initially performed repeat annotation on both genomes and masked the repetitive regions to improve the efficiency and accuracy of genome structural annotation. The results indicated that 205,650 and 197,501 transposable elements (TEs) were identified in the LHH and Newhall genomes, with total lengths of 115.70 Mb and 116.79 Mb, respectively, accounting for 34.00% and 33.72% of each genome (Table S6). Among these, LTR/Gypsy elements were the most abundant, comprising 11.37% and 12.69% of the LHH and Newhall genomes, respectively (Table S6). Furthermore, class I transposons, or retrotransposons, were found to be more prevalent than class II transposons, or DNA transposons, in both genomes, consistent with patterns observed in other plant species [60]. A comparison with the distribution of TEs revealed a high density of TEs in the central regions of the chromosomes, corresponding to the B compartment areas, which are characterized by low gene density, low transcriptional activity, and high chromatin condensation (Fig. 3A, B).

Subsequently, a comprehensive annotation strategy was implemented in the study, integrating ab initio, homology-based, and transcript evidence-based approaches to annotate gene structures in the repeat-masked LHH and Newhall genomes (Table S7). The analysis identified 31,456 and 30,021 gene

models in the LHH and Newhall genomes, respectively (Fig. S2, Table S7). Further statistical analysis of these gene models revealed that the distribution of annotated genes, coding sequences (CDS), exons, and intron lengths in both genomes were comparable to those reported in previously described sweet orange and closely related species genomes [61–63]. This consistency underscores the accuracy and reliability of the annotated gene models (Fig. 4).

Functional annotation results show that 96.66% and 97.6% of the genes in the LHH and Newhall genomes, respectively, were successfully annotated using these resources. Moreover, the study focused on identifying non-coding RNAs in both genomes. The analysis revealed that 411 and 418 tRNAs, 714 and 3,920 rRNAs, and 167 and 166 miRNAs were annotated in the LHH and Newhall genomes, respectively (Table S8).

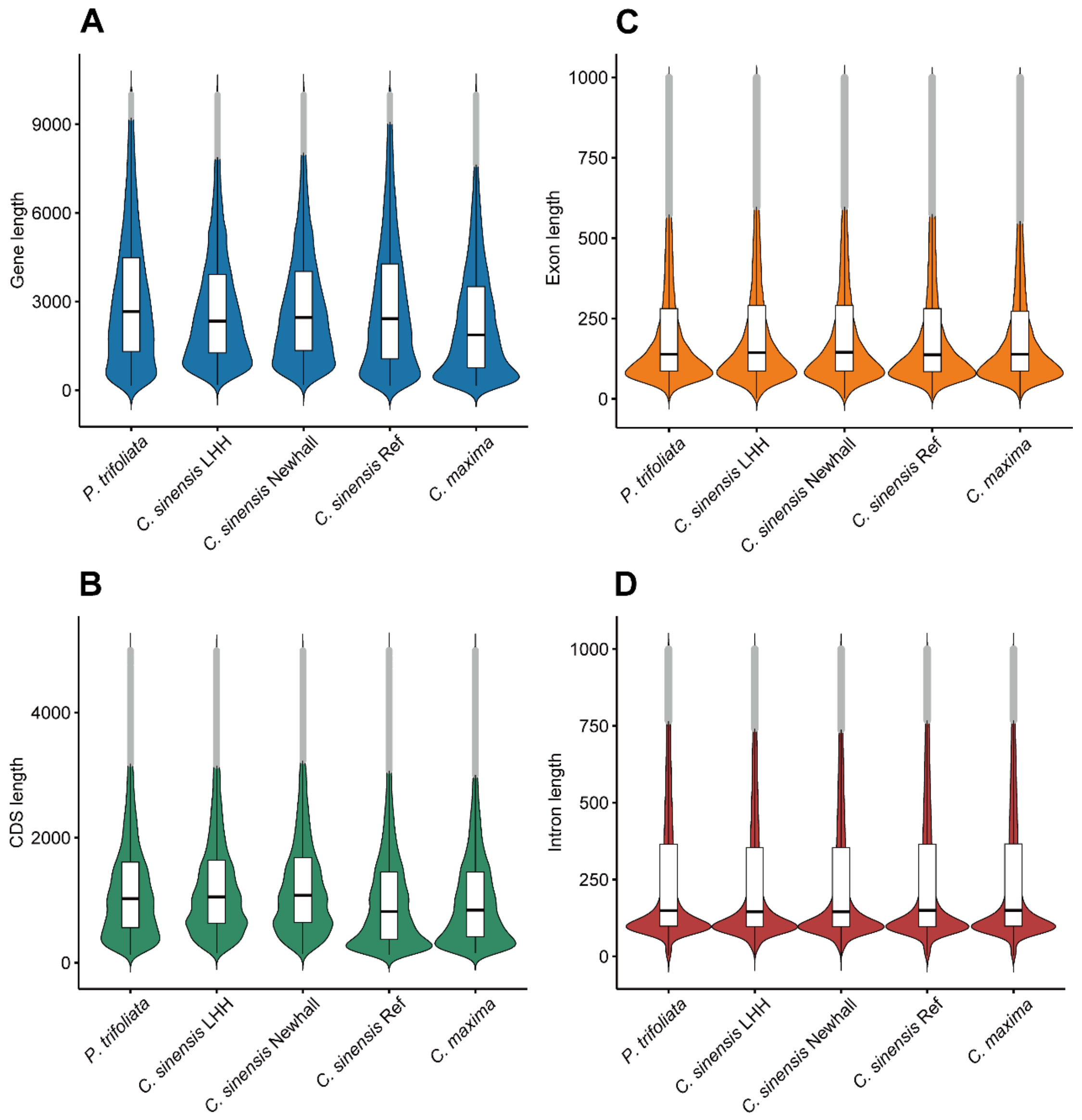

Figure 4: Statistical analysis and assessment of the annotation results for the *C. sinensis* LHH and Newhall genome. Comparison of gene length (A), CDS length (B), exon length (C) and intron length (D) distribution between the genomes of LHH, Newhall and closely related species. *C. sinensis* Ref refers to the sweet orange reference genome obtained from the NCBI database, with the corresponding RefSeq ID GCF_022201045.2.

Table 3: Statistics of function annotation results for *C. sinensis* LHH and Newhall

	C. sinensis LHH		*C. sinensis* Newhall	
Database	Annotated number	Annotated ratio (%)	Annotated number	Annotated ratio (%)
GO	24,741	78.65	23,881	79.55
KEGG	23,558	74.89	22,846	76.1
KOG	16,155	51.36	15,641	52.1
Pfam	25,420	80.81	24,722	82.35
SWISS-PROT	21,482	68.29	20,922	69.69
TrEMBL	30,266	96.22	29,198	97.26
eggNOG	25,306	80.45	24,461	81.48
NR	30,106	95.71	29,078	96.86
Total	30,404	96.66	29,299	97.60

Comparative genomics and evolutionary analysis

Based on the T2T genomes of LHH and Newhall, the study aimed to elucidate the evolutionary history of the navel orange genome. A total of 392,184 genes from 13 species (*Amborella trichopoda*, *Oryza sativa*, *Solanum lycopersicum*, *Vitis vinifera*, *Ziziphus jujuba*, *Malus domestica*, *Arabidopsis thaliana*, *Poncirus trifoliata*, *Citrus maxima*, *Citrus clementina*, *Citrus sinensis* Ref, *Citrus sinensis* LHH *and Citrus sinensis* Newhall) were clustered into 43,324 gene families. Among these, 3,331 gene families were found to be common to all 13 species. Specifically, 30,442 and 29,395 genes from LHH and Newhall were clustered into 22,554 and 22,022 gene families, respectively, with 282 and 122 gene families being unique to each (Fig. S3A, Table S9). Analysis of five species within the Citrus genus revealed that 13,531 gene families were common across all five species, while LHH and Newhall had 282 and 122 unique gene families, respectively (Fig. S3B). A review of gene copy numbers indicated that the proportion of genes with varying copy numbers in the LHH and Newhall genomes was comparable, with single-copy genes being the most prevalent (Fig. 5A).

Through gene family clustering analysis, 926 single-copy orthologs were identified across the 13 species. Based on the phylogenetic analysis of these single-copy orthologs, the five Citrus species, along with the closely related species *P. trifoliata*, clustered within the same branch, demonstrating high accuracy and reliability of the phylogenetic tree. Furthermore, we estimated that the divergence time between *P. trifoliata* and the five Citrus species occurred approximately 13.07 million years ago (Mya), during the Miocene epoch (Fig. 5A).

The analysis of gene family expansion and contraction across the genomes revealed that LHH and Newhall have 907 and 770 expanded gene families, respectively. Additionally, LHH has 673 contracted gene families, while Newhall has 1,141 contracted gene families (Fig. 5A). The distribution of the synonymous substitution rate (*Ks*) indicates that the *C. sinensis* LHH and Newhall genomes have undergone an ancient whole-genome triplication (WGT) event (Ks = 1.56), which is consistent with previous findings [10] (Fig. 5B). The insertion of Long Terminal Repeat Retrotransposons (LTR-RTs) plays a crucial role in the evolution of plant genomes [64,65]. The study analyzed the insertion times and genomic positions of LTR-RTs identified in LHH and Newhall. In the three *C. sinensis* genomes, a higher density of recent LTR-RT insertions was observed compared to its closely related species, *P. trifoliata*. This increased density may have contributed to the larger genome size of *C. sinensis* relative to *P. trifoliata* [66]. These LTR-RT insertions were widespread across sweet orange chromosomes and were also found within euchromatic regions, likely due to the ongoing amplification of LTR-RTs [60]. Furthermore, certain regions exhibited a high-density distribution of recent insertions, suggesting the presence of active LTR-RTs in these areas (Fig. 5C).

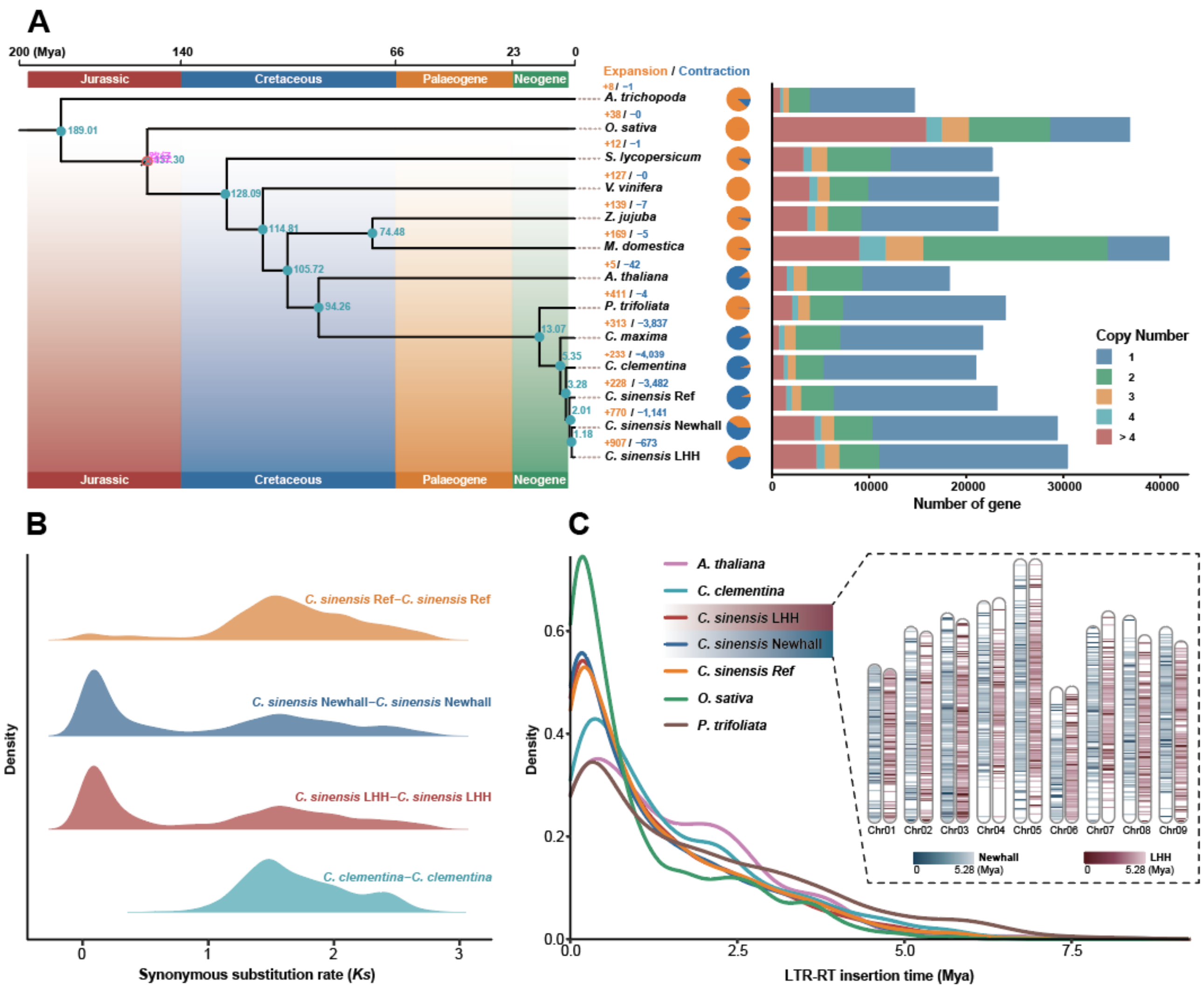

Figure 5: The evolutionary history of *C. sinensis* LHH and Newhall genomes. (A), Phylogenetic tree of *C. sinensis* LHH and Newhall along with 11 other plant genomes. Pie charts depict the expansion and contraction of gene families, with orange indicating expansion and blue indicating contraction. Corresponding values are displayed in matching colors above each chart. Nodes on the evolutionary tree are labeled with estimated divergence times (in Mya). The number of gene copies in each species' genome is shown on the right side. (B), The distribution of *Ks* values illustrates WGT events in the evolution of the *C. sinensis* LHH and Newhall genomes. (C), The distribution of full-length LTR-RT insertions in LHH and Newhall genomes. The density plot illustrates the temporal distribution of full-length LTR-RT insertions in plant genomes, including LHH and Newhall genomes. The chromosome

heatmap provides a comprehensive view of the spatial distribution and temporal information of full-length LTR-RT insertions on each chromosome of LHH and Newhall genomes.

Genomic variation between the LHH and Newhall genomes

To elucidate the sequence differences between the genomes of navel orange LHH and Newhall, and to provide a foundational dataset for understanding their phenotypic diversity, collinear regions and variations between the two genomes were identified. Collinearity analysis was conducted between the genomes, and single-nucleotide polymorphisms (SNPs), as well as presence/absence variations (PAVs), were called. Subsequently, the results of genomic colinearity analysis revealed 6,075 collinear blocks between the two genomes. The lengths of these blocks amounted to 289.07 Mb in the LHH genome and 289.54 Mb in the Newhall genome. These blocks account for 372.71 Mb and 370.23 Mb, or 77.56% and 78.21% of the respective genomes, indicating that most regions in both genomes are conserved. The density of SNPs showed differential distributions across the chromosomes of the LHH and Newhall genomes. Chromosomes 3, 5, and 9 were observed to have an abundance of SNPs, with chromosome 9 containing the highest number at 410,667 (Fig. 6). Additionally, these three chromosomes exhibited more PAVs compared to the other chromosomes. Genome structural variations (SVs) can affect gene expression through various mechanisms [67] and have been reported to contribute to phenotypic diversity in eukaryotes, driving the diversity of functional genes in crops [68]. SVs are considered to have a greater impact on gene expression and protein function compared to SNPs [69]. A total of 2,886 SVs were detected between the LHH and Newhall genomes, with insertions and deletions being the most common types of structural variations, numbering 1,383 and 1,313, respectively (Fig. S5A). Among these structural variations, 164 were further identified as haplotype-

specific structural variations. The distribution and types of SVs on different chromosomes revealed that chromosomes 5, 6, and 9 had a higher number of structural variations. Genome-wide analysis indicated that insertions and deletions were the predominant types of structural variations. The positions of these SVs relative to gene models in the Newhall genome were further annotated. The results revealed significant differences in the distribution of SVs at various relative positions across the chromosomes. Chromosomes 5, 6, and 9 had the highest proportion of SVs located in exon regions, with 338, 203, and 220 SVs, respectively, accounting for 31.98%, 25.66%, and 31.38% of the total SVs on each chromosome (Fig. S5B). These findings indicate substantial genomic structural diversity between the LHH and Newhall genomes. The extensive variation information within these two genomes offers valuable resources for identifying and utilizing alleles associated with superior agronomic traits.

Previously reported rice cold tolerance-related genes were collected and mapped onto the Newhall genome to identify homologous genes. By integrating information on detected SVs, several homologous genes were found to have SVs in their upstream, downstream, or intronic regions. These SVs may be linked to the differences in cold resistance between Newhall and LHH (Table S10).

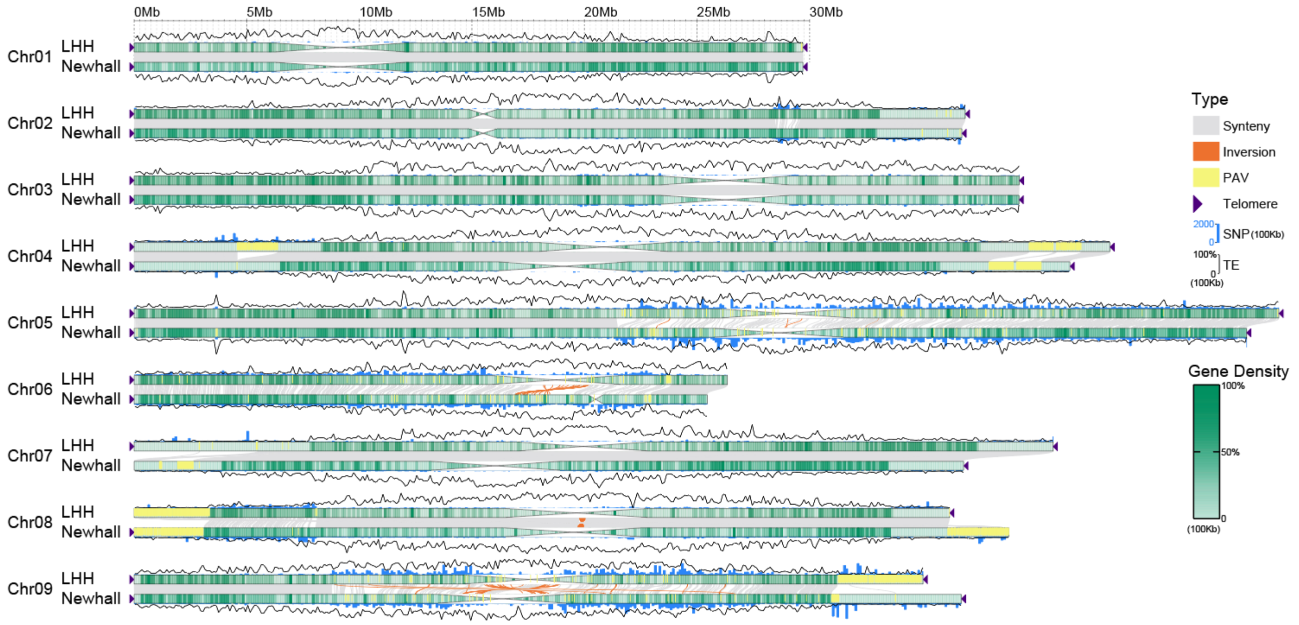

Figure 6: Variations between LHH and Newhall genomes. Collinear regions are connected by grey lines, while inversion regions are connected by orange lines. PAV regions are represented by yellow blocks. Black triangles indicate detected telomere repeat sequences. Gene density heatmaps are plotted on each chromosome with a 100-kb unit. SNP and TE percentage distribution are plotted above or below each chromosome in 100-kb units.

Reuse potential

This study successfully assembled two T2T reference genomes for navel orange, providing high-quality genomic resources for future multi-omics and molecular biology research in sweet orange. These reference genomes will significantly enhance the utilization of multi-omics data and improve the accuracy of downstream analyses.

The two navel orange varieties selected for this research exhibit notable differences in important agronomic traits such as flesh color, photosynthetic efficiency, and cold tolerance. Future studies can leverage the high-quality reference genomes generated in this study to construct a pangenome for

sweet orange. By incorporating phenotypic information from representative materials, further screening of the various types of variations identified between the LHH and Newhall genomes in this study, including SNPs, SVs, and PAVs, can be undertaken. Subsequent in vivo and in vitro experiments can validate the impact of these structural variations on phenotypes, allowing for the characterization of genes related to important agronomic traits and the understanding of how these variations influence gene function.

Additionally, forthcoming research can use the two T2T genomes as references to analyze population resequencing data of sweet orange. Genome-wide association studies (GWAS) based on SNPs, PAVs, and SVs can then be employed to identify and exploit genomic variations with substantial effects.

Finally, high-quality genomes are crucial for accurate gene prediction. The T2T genomes of the two navel orange varieties will facilitate further improvement in gene annotation quality. Future studies can refine sweet orange gene annotations using full-length transcriptome sequencing and manually validate and correct the annotation result.

Discussion

In this study, we performed de novo assembly and annotation of the genomes of two navel orange varieties that differ in key agronomic traits such as flesh color, photosynthetic efficiency, and cold tolerance. We utilized a comprehensive approach integrating PacBio HiFi, ONT UL, Hi-C, and Illumina PE sequencing data. With the support of advanced sequencing technologies and genome assembly algorithms and strategies, we successfully assembled two T2T reference genomes for sweet orange, achieving high continuity, completeness, and accuracy. These assemblies have unveiled the

sequences of highly repetitive regions, including centromeres and telomeres, within the sweet orange genomes. Through comparative genomic analysis, we elucidated the evolutionary history of the sweet orange genomes, including WGD events, gene family expansions and contractions, divergence times between species, and the amplification of LTR-RTs. Collinear regions account for over 77% of the sequences between the two genomes, indicating significant conservation. On the other hand, extensive intraspecies variations were detected, including 2,886 structural variations (SVs). We categorized these SVs and mapped their relative positions in the genome. These findings provide a valuable set of candidate genes, narrowing the scope for subsequent studies aimed at characterizing and validating genes associated with agronomic trait differences between the two sweet orange varieties. This study lays a high-quality data foundation for understanding the phenotypic and genetic diversity of sweet oranges.

The pangenome is a current research hotspot in plant genomics. Gap-free genomes can serve as high-quality references for constructing more comprehensive and accurate graph-based pangenomes, which, in turn, facilitate the study of polymorphisms in complex genomic regions [70]. The two T2T sweet orange reference genomes provided in this study enhance the existing sweet orange genomic resources. These genomes will support the construction of a graph-based pangenome for sweet orange, enable the detection and genotyping of a broader range of structural variations (SVs), and, when combined with population resequencing data, help uncover the associations between sweet orange phenotypes and genotypes.

Data Availability

The genome sequences and raw sequencing data for the Newhall and LHH navel orange genomes

are available under NCBI BioProject ID PRJNA1122682. The raw sequencing data for the Newhall and LHH navel orange genomes are available from the National Genomics Data Center (NGDC) [71], under accession number PRJCA026660. The PacBio HiFi, Hi-C, ONT UL, Illumina PE, and RNA-seq sequencing data for this project can be accessed through NCBI BioProject PRJNA1122682, with corresponding numbers SRR29362821 to SRR29362830. The LHH and Newhall genomes have been deposited in DDBJ/ENA/GenBank under accession numbers JBFBVJ000000000 and JBFBVK000000000, respectively. The genomic data of Longhuihong and Newhall, as well as the other supporting data can be found in the *GigaScience* GigaDB database [72-74].

Additional Files

Supplementary Figure 1. BUSCO assessment results for the genomes of LHH and Newhall.

Supplementary Figure 2. Statistics of integrated gene models predicted by three strategies. The Venn diagram depicts the quantities of gene models predicted by three strategies in LHH (A) and Newhall (B) genomes, along with their overlapping relationships.

Supplementary Figure 3. Gene family clustering of 13 plant genomes. The Venn diagrams show unique and shared orthologous gene clusters among the 13 species (A) and the 5 Citrus genomes (B).

Supplementary Figure 4. Dot matrix comparison of the LHH and Newhall genomes. The x-axis represents the LHH genome coordinates, and the y-axis represents the Newhall genome coordinates.

Supplementary Figure 5. (A), Composition of SVs between LHH and NHE genomes. The pie chart shows the proportion of different types of SVs across the whole genome. The stacked bar plot shows the types and quantities of SVs on each chromosome. (B), Positional distribution of SVs on each chromosome relative to the Newhall gene.

Supplementary Table 1. Statistics of Hi-C assembly results for the Newhall and LHH genomes

Supplementary Table 2. Telomere location information for Newhall and LHH genomes

Supplementary Table 3. Centromere location information for Newhall and LHH genomes

Supplementary Table 4. Summary of BUSCO assessment results for LHH and Newhall genomes

Supplementary Table 5. Mapping rate statistics of sequencing data for LHH and Newhall genomes

Supplementary Table 6. Statistical information of TEs in LHH and Newhall genomes

Supplementary Table 7. Gene model annotation results of LHH and Newhall genomes

Supplementary Table 8. Summary of non-coding RNA annotation results in LHH and Newhall genomes

Supplementary Table 9. Statistical summary of gene family clustering results

Supplementary Table 10. SVs of homologous genes in the Newhall genome corresponding to reported cold tolerance genes in *O. sativa* Nipponbare

Abbreviations

T2T: telomere-to-telomere; Mb: megabases; Gb: gigabase; ONT UL: Oxford Nanopore Technologies Ultra Long; HiFi: High Fidelity; Hi-C: high-throughput chromosome conformation capture; CCS: Circular Consensus Sequencing; PE: pair end; Q-PCR: quantitative polymerase chain reaction; LTR: long terminal repeat; LAI: LTR Assembly Index; QV: quality value; TEs: transposable elements; CDS: coding sequence; tRNA: transfer RNA; miRNA: microRNA; snoRNA: small nucleolar RNA; snRNA: small nuclear RNA; MSA: multiple sequence alignment; ML: maximum likelihood; Mya: million years ago; WGD: whole genome duplication; *Ks*: synonymous substitution rates; SNPs: single-nucleotide polymorphisms; PAVs: presence/absence variations; SVs: structural

variations; GWAS: genome-wide association studies.

Competing Interests

The authors declare that they have no competing interests.

Funding

This work was financed by Ministry of Agriculture and rural citrus industry cluster project and Chongqing academy of agricultural sciences municipal financial special project (NKY-2022AB005)

Authors' Contributions

L.H. and J.-M.S. conceived the idea, supervised the work, and revised the manuscript. M.W., S.L. and H.Y. prepared the plant materials. L.H., J.-M.S., X.-D.X., L.Y., S.-Y.Y. and L.-L.C. analyzed the data. L.H., J.-M.S., X.-D.X. and L.Y. wrote the original draft and revised the manuscript. L.H., X.-D.X. and L.Y. contributed equally to this work. All authors have read and approved the final manuscript.

Acknowledgments

We thank Biomarker Technologies Co., Ltd for assisting in sequencing.

References

1. Safdar MN, Kausar T, Jabbar S, Mumtaz A, Ahad K, Saddozai AA. Extraction and quantification of polyphenols from kinnow (Citrus reticulate L.) peel using ultrasound and maceration techniques. *J Food Drug Anal*. 2017; doi: 10.1016/j.jfda.2016.07.010.

2. Zou Z, Xi W, Hu Y, Nie C, Zhou Z. Antioxidant activity of Citrus fruits. *Food Chem*. 2016; doi: 10.1016/j.foodchem.2015.09.072.

3. Farag MA, Abib B, Ayad L, Khattab AR. Sweet and bitter oranges: An updated comparative review of their bioactives, nutrition, food quality, therapeutic merits and biowaste valorization practices. *Food Chem*. 2020; doi: 10.1016/j.foodchem.2020.127306.

4. Seminara S, Bennici S, Di Guardo M, Caruso M, Gentile A, La Malfa S, et al.. Sweet Orange: Evolution, Characterization, Varieties, and Breeding Perspectives. *Agriculture*. 2023; doi: 10.3390/agriculture13020264.

5. Carlos Merino, Aurea Hervalejo, Arturo Salguero, David GonzÃ¡lez, Francisco J. Arenas-Arenas. YIELD AND FRUIT QUALITY OF TWO EARLY MATURING ORANGE CULTIVARS, “NAVELINA” AND “FUKUMOTO”, IN ANDALUSIA, SPAIN. *Acta Horticulturae*. International Society for Horticultural Science (ISHS), Leuven, Belgium; 2015. DOI:10.17660/ActaHortic.2015.1065.29

6. Wu GA, Terol J, Ibanez V, López-García A, Pérez-Román E, Borredá C, et al.. Genomics of the origin and evolution of Citrus. *Nature*. 2018; doi: 10.1038/nature25447.

7. Wang L, Huang Y, Liu Z, He J, Jiang X, He F, et al.. Somatic variations led to the selection of acidic and acidless orange cultivars. *Nat Plants*. 2021; doi: 10.1038/s41477-021-00941-x.

8. The Arabidopsis Genome Initiative. Analysis of the genome sequence of the flowering plant Arabidopsis thaliana. *Nature*. 2000; doi: 10.1038/35048692.

9. Shendure J, Balasubramanian S, Church GM, Gilbert W, Rogers J, Schloss JA, et al.. DNA sequencing at 40: past, present and future. *Nature*. 2017; doi: 10.1038/nature24286.

10. Xu Q, Chen L-L, Ruan X, Chen D, Zhu A, Chen C, et al.. The draft genome of sweet orange (Citrus

sinensis). *Nat Genet*. 2013; doi: 10.1038/ng.2472.

11. Wu GA, Prochnik S, Jenkins J, Salse J, Hellsten U, Murat F, et al.. Sequencing of diverse mandarin, pummelo and orange genomes reveals complex history of admixture during citrus domestication. *Nat Biotechnol*. 2014; doi: 10.1038/nbt.2906.

12. Wang X, Xu Y, Zhang S, Cao L, Huang Y, Cheng J, et al.. Genomic analyses of primitive, wild and cultivated citrus provide insights into asexual reproduction. *Nat Genet*. 2017; doi: 10.1038/ng.3839.

13. Huang Y, He J, Xu Y, Zheng W, Wang S, Chen P, et al.. Pangenome analysis provides insight into the evolution of the orange subfamily and a key gene for citric acid accumulation in citrus fruits. *Nat Genet*. 2023; doi: 10.1038/s41588-023-01516-6.

14. Bao Y, Zeng Z, Yao W, Chen X, Jiang M, Sehrish A, et al.. A gap-free and haplotype-resolved lemon genome provides insights into flavor synthesis and huanglongbing (HLB) tolerance. *Hortic Res*. 2023; doi: 10.1093/hr/uhad020.

15. Yang L, Deng H, Wang M, Li S, Wang W, Yang H, et al.. A high-quality chromosome-scale genome assembly of blood orange, an important pigmented sweet orange variety. *Sci Data*. 2024; doi: 10.1038/s41597-024-03313-0.

16. Abu Almakarem AS, Heilman KL, Conger HL, Shtarkman YM, Rogers SO. Extraction of DNA from plant and fungus tissues in situ. *BMC Res Notes*. 2012; doi: 10.1186/1756-0500-5-266.

17. Marçais G, Kingsford C. A fast, lock-free approach for efficient parallel counting of occurrences of k-mers. *Bioinformatics*. 2011; doi: 10.1093/bioinformatics/btr011.

18. Ranallo-Benavidez TR, Jaron KS, Schatz MC. GenomeScope 2.0 and Smudgeplot for reference-free profiling of polyploid genomes. *Nat Commun*. 2020; doi: 10.1038/s41467-020-14998-3.

19. Cheng H, Concepcion GT, Feng X, Zhang H, Li H. Haplotype-resolved de novo assembly using phased assembly graphs with hifiasm. *Nat Methods*. 2021; doi: 10.1038/s41592-020-01056-5.

20. Burton JN, Adey A, Patwardhan RP, Qiu R, Kitzman JO, Shendure J. Chromosome-scale scaffolding of de novo genome assemblies based on chromatin interactions. *Nat Biotechnol*. 2013; doi: 10.1038/nbt.2727.

21. Durand NC, Robinson JT, Shamim MS, Machol I, Mesirov JP, Lander ES, et al.. Juicebox Provides a Visualization System for Hi-C Contact Maps with Unlimited Zoom. *Cell Syst*. 2016; doi: 10.1016/j.cels.2015.07.012.

22. Xu M, Guo L, Gu S, Wang O, Zhang R, Peters BA, et al.. TGS-GapCloser: A fast and accurate gap closer for large genomes with low coverage of error-prone long reads. *Gigascience*. 2020; doi: 10.1093/gigascience/giaa094.

23. Xu D, Yang J, Wen H, Feng W, Zhang X, Hui X, et al.. CentIER: accurate centromere identification for plant genome. *Plant Comm*. 2024; doi: 10.1016/j.xplc.2024.101046.

24. Seppey M, Manni M, Zdobnov EM. BUSCO: Assessing Genome Assembly and Annotation Completeness. *Methods Mol Biol*. 2019; doi: 10.1007/978-1-4939-9173-0_14.

25. Li H. Minimap2: pairwise alignment for nucleotide sequences. *Bioinformatics*. 2018; doi: 10.1093/bioinformatics/bty191.

26. Ou S, Jiang N. LTR_retriever: A Highly Accurate and Sensitive Program for Identification of Long Terminal Repeat Retrotransposons. *Plant Physiol*. 2018; doi: 10.1104/pp.17.01310.

27. Ou S, Chen J, Jiang N. Assessing genome assembly quality using the LTR Assembly Index (LAI). *Nucleic Acids Res*. 2018; doi: 10.1093/nar/gky730.

28. Rhie A, Walenz BP, Koren S, Phillippy AM. Merqury: reference-free quality, completeness, and

phasing assessment for genome assemblies. *Genome Biol*. 2020; doi: 10.1186/s13059-020-02134-9.

29. Flynn JM, Hubley R, Goubert C, Rosen J, Clark AG, Feschotte C, et al.. RepeatModeler2 for automated genomic discovery of transposable element families. *Proc Natl Acad Sci U S A*. 2020; doi: 10.1073/pnas.1921046117.

30. Bao W, Kojima KK, Kohany O. Repbase Update, a database of repetitive elements in eukaryotic genomes. *Mob DNA*. 2015; doi: 10.1186/s13100-015-0041-9.

31. Stanke M, Diekhans M, Baertsch R, Haussler D. Using native and syntenically mapped cDNA alignments to improve de novo gene finding. *Bioinformatics*. 2008; doi: 10.1093/bioinformatics/btn013.

32. Korf I. Gene finding in novel genomes. *BMC Bioinformatics*. 2004; doi: 10.1186/1471-2105-5-59.

33. Keilwagen J, Wenk M, Erickson JL, Schattat MH, Grau J, Hartung F. Using intron position conservation for homology-based gene prediction. *Nucleic Acids Res*. 2016; doi: 10.1093/nar/gkw092.

34. Kim D, Langmead B, Salzberg SL. HISAT: a fast spliced aligner with low memory requirements. *Nat Methods*. 2015; doi: 10.1038/nmeth.3317.

35. Pertea M, Pertea GM, Antonescu CM, Chang T-C, Mendell JT, Salzberg SL. StringTie enables improved reconstruction of a transcriptome from RNA-seq reads. *Nat Biotechnol*. 2015; doi: 10.1038/nbt.3122.

36. Tang S, Lomsadze A, Borodovsky M. Identification of protein coding regions in RNA transcripts. *Nucleic Acids Res*. 2015; doi: 10.1093/nar/gkv227.

37. Grabherr MG, Haas BJ, Yassour M, Levin JZ, Thompson DA, Amit I, et al.. Trinity: reconstructing

a full-length transcriptome without a genome from RNA-Seq data. *Nat Biotechnol*. 2011; doi: 10.1038/nbt.1883.

38. Haas BJ, Delcher AL, Mount SM, Wortman JR, Smith RK, Hannick LI, et al.. Improving the Arabidopsis genome annotation using maximal transcript alignment assemblies. *Nucleic Acids Res*. 2003; doi: 10.1093/nar/gkg770.

39. Haas BJ, Salzberg SL, Zhu W, Pertea M, Allen JE, Orvis J, et al.. Automated eukaryotic gene structure annotation using EVidenceModeler and the Program to Assemble Spliced Alignments. *Genome Biol*. 2008; doi: 10.1186/gb-2008-9-1-r7.

40. Wolff J, Rabbani L, Gilsbach R, Richard G, Manke T, Backofen R, et al.. Galaxy HiCExplorer 3: a web server for reproducible Hi-C, capture Hi-C and single-cell Hi-C data analysis, quality control and visualization. *Nucleic Acids Res*. 2020; doi: 10.1093/nar/gkaa220.

41. Huerta-Cepas J, Szklarczyk D, Heller D, Hernández-Plaza A, Forslund SK, Cook H, et al.. eggNOG 5.0: a hierarchical, functionally and phylogenetically annotated orthology resource based on 5090 organisms and 2502 viruses. *Nucleic Acids Res*. 2019; doi: 10.1093/nar/gky1085.

42. Kanehisa M, Sato Y, Kawashima M, Furumichi M, Tanabe M. KEGG as a reference resource for gene and protein annotation. *Nucleic Acids Res*. 2016; doi: 10.1093/nar/gkv1070.

43. Boeckmann B, Bairoch A, Apweiler R, Blatter M-C, Estreicher A, Gasteiger E, et al.. The SWISS-PROT protein knowledgebase and its supplement TrEMBL in 2003. *Nucleic Acids Res*. 2003; doi: 10.1093/nar/gkg095.

44. Finn RD, Mistry J, Schuster-Böckler B, Griffiths-Jones S, Hollich V, Lassmann T, et al.. Pfam: clans, web tools and services. *Nucleic Acids Res*. 2006; doi: 10.1093/nar/gkj149.

45. Lowe TM, Eddy SR. tRNAscan-SE: a program for improved detection of transfer RNA genes in

genomic sequence. *Nucleic Acids Res*. 1997; doi: 10.1093/nar/25.5.955.

46. Griffiths-Jones S, Moxon S, Marshall M, Khanna A, Eddy SR, Bateman A. Rfam: annotating non-coding RNAs in complete genomes. *Nucleic Acids Res*. 2005; doi: 10.1093/nar/gki081.

47. Nawrocki EP, Eddy SR. Infernal 1.1: 100-fold faster RNA homology searches. *Bioinformatics*. 2013; doi: 10.1093/bioinformatics/btt509.

48. Katoh K, Misawa K, Kuma K, Miyata T. MAFFT: a novel method for rapid multiple sequence alignment based on fast Fourier transform. *Nucleic Acids Res*. 2002; doi: 10.1093/nar/gkf436.

49. Castresana J. Selection of conserved blocks from multiple alignments for their use in phylogenetic analysis. *Mol Biol Evol*. 2000; doi: 10.1093/oxfordjournals.molbev.a026334.

50. Minh BQ, Schmidt HA, Chernomor O, Schrempf D, Woodhams MD, von Haeseler A, et al.. IQ-TREE 2: New Models and Efficient Methods for Phylogenetic Inference in the Genomic Era. *Mol Biol Evol*. 2020; doi: 10.1093/molbev/msaa015.

51. Emms DM, Kelly S. OrthoFinder: phylogenetic orthology inference for comparative genomics. *Genome Biol*. 2019; doi: 10.1186/s13059-019-1832-y.

52. De Bie T, Cristianini N, Demuth JP, Hahn MW. CAFE: a computational tool for the study of gene family evolution. *Bioinformatics*. 2006; doi: 10.1093/bioinformatics/btl097.

53. Yang Z. PAML 4: phylogenetic analysis by maximum likelihood. *Mol Biol Evol*. 2007; doi: 10.1093/molbev/msm088.

54. Marçais G, Delcher AL, Phillippy AM, Coston R, Salzberg SL, Zimin A. MUMmer4: A fast and versatile genome alignment system. *PLoS Comput Biol*. 2018; doi: 10.1371/journal.pcbi.1005944.

55. Zhou Z-W, Yu Z-G, Huang X-M, Liu J-S, Guo Y-X, Chen L-L, et al.. GenomeSyn: a bioinformatics tool for visualizing genome synteny and structural variations. *J Genet Genomics*. 2022; doi:

10.1016/j.jgg.2022.03.013.

56. Boratyn GM, Schäffer AA, Agarwala R, Altschul SF, Lipman DJ, Madden TL. Domain enhanced lookup time accelerated BLAST. *Biol Direct*. 2012; doi: 10.1186/1745-6150-7-12.

57. Poplin R, Chang P-C, Alexander D, Schwartz S, Colthurst T, Ku A, et al.. A universal SNP and small-indel variant caller using deep neural networks. *Nat Biotechnol*. 2018; doi: 10.1038/nbt.4235.

58. Xie L, Gong X, Yang K, Huang Y, Zhang S, Shen L, et al.. Technology-enabled great leap in deciphering plant genomes. *Nat Plants*. 2024; doi: 10.1038/s41477-024-01655-6.

59. Parra G, Bradnam K, Korf I. CEGMA: a pipeline to accurately annotate core genes in eukaryotic genomes. *Bioinformatics*. 2007; doi: 10.1093/bioinformatics/btm071.

60. Song J-M, Guan Z, Hu J, Guo C, Yang Z, Wang S, et al.. Eight high-quality genomes reveal pan-genome architecture and ecotype differentiation of Brassica napus. *Nat Plants*. 2020; doi: 10.1038/s41477-019-0577-7.

61. Wu B, Yu Q, Deng Z, Duan Y, Luo F, Gmitter F. A chromosome-level phased genome enabling allele-level studies in sweet orange: a case study on citrus Huanglongbing tolerance. *Hortic Res*. 2023; doi: 10.1093/hr/uhac247.

62. Peng Z, Bredeson JV, Wu GA, Shu S, Rawat N, Du D, et al.. A chromosome-scale reference genome of trifoliate orange (Poncirus trifoliata) provides insights into disease resistance, cold tolerance and genome evolution in Citrus. *The Plant Journal*. 2020; doi: 10.1111/tpj.14993.

63. Liu H, Wang X, Liu S, Huang Y, Guo Y-X, Xie W-Z, et al.. Citrus Pan-Genome to Breeding Database (CPBD): A comprehensive genome database for citrus breeding. *Mol Plant*. 2022; doi: 10.1016/j.molp.2022.08.006.

64. Li S-F, She H-B, Yang L-L, Lan L-N, Zhang X-Y, Wang L-Y, et al.. Impact of LTR-Retrotransposons on Genome Structure, Evolution, and Function in Curcurbitaceae Species. *International Journal of Molecular Sciences*. Multidisciplinary Digital Publishing Institute; 2022; doi: 10.3390/ijms231710158.

65. Choi JY, Purugganan MD. Evolutionary Epigenomics of Retrotransposon-Mediated Methylation Spreading in Rice. *Mol Biol Evol*. 2018; doi: 10.1093/molbev/msx284.

66. Zhang T, Qiao Q, Novikova PY, Wang Q, Yue J, Guan Y, et al.. Genome of Crucihimalaya himalaica, a close relative of Arabidopsis, shows ecological adaptation to high altitude. *Proc Natl Acad Sci U S A*. 2019; doi: 10.1073/pnas.1817580116.

67. Hollox EJ, Zuccherato LW, Tucci S. Genome structural variation in human evolution. *Trends Genet*. 2022; doi: 10.1016/j.tig.2021.06.015.

68. Chawla HS, Lee H, Gabur I, Vollrath P, Tamilselvan-Nattar-Amutha S, Obermeier C, et al.. Long-read sequencing reveals widespread intragenic structural variants in a recent allopolyploid crop plant. *Plant Biotechnology Journal*. 2021; doi: 10.1111/pbi.13456.

69. Yuan Y, Bayer PE, Batley J, Edwards D. Current status of structural variation studies in plants. *Plant Biotechnology Journal*. 2021; doi: 10.1111/pbi.13646.

70. Wang S, Qian Y-Q, Zhao R-P, Chen L-L, Song J-M. Graph-based pan-genomes: increased opportunities in plant genomics. *J Exp Bot*. 2023; doi: 10.1093/jxb/erac412.

71. CNCB-NGDC Members and Partners. Database Resources of the National Genomics Data Center, China National Center for Bioinformation in 2023. *Nucleic Acids Res*. 2023; doi: 10.1093/nar/gkac1073.

72. Hong L, Xu X, Yang L, Wang M, Li S, Yang H, et al.. The genomic data of Longhuihong (Citrus

sinensis [L.] Osb. cv. LHH). GigaScience Database. 2024. https://doi.org/10.5524/102578

73. Hong L, Xu X, Yang L, Wang M, Li S, Yang H, et al.. The genomic data of Newhall (Citrus sinensis [L.] Osb. cv. Newhall) GigaScience Database. 2024. https://doi.org/10.5524/102579

74. Hong L, Xu X, Yang L, Wang M, Li S, Yang H, et al.. Supporting data for "Construction and analysis of telomere-to-telomere genomes for two sweet oranges: Longhuihong and Newhall (Citrus sinensis)" GigaScience Database. 2024. https://doi.org/10.5524/102577

Figure 2

Click here to access/download;Figure;Figure 2.pdf

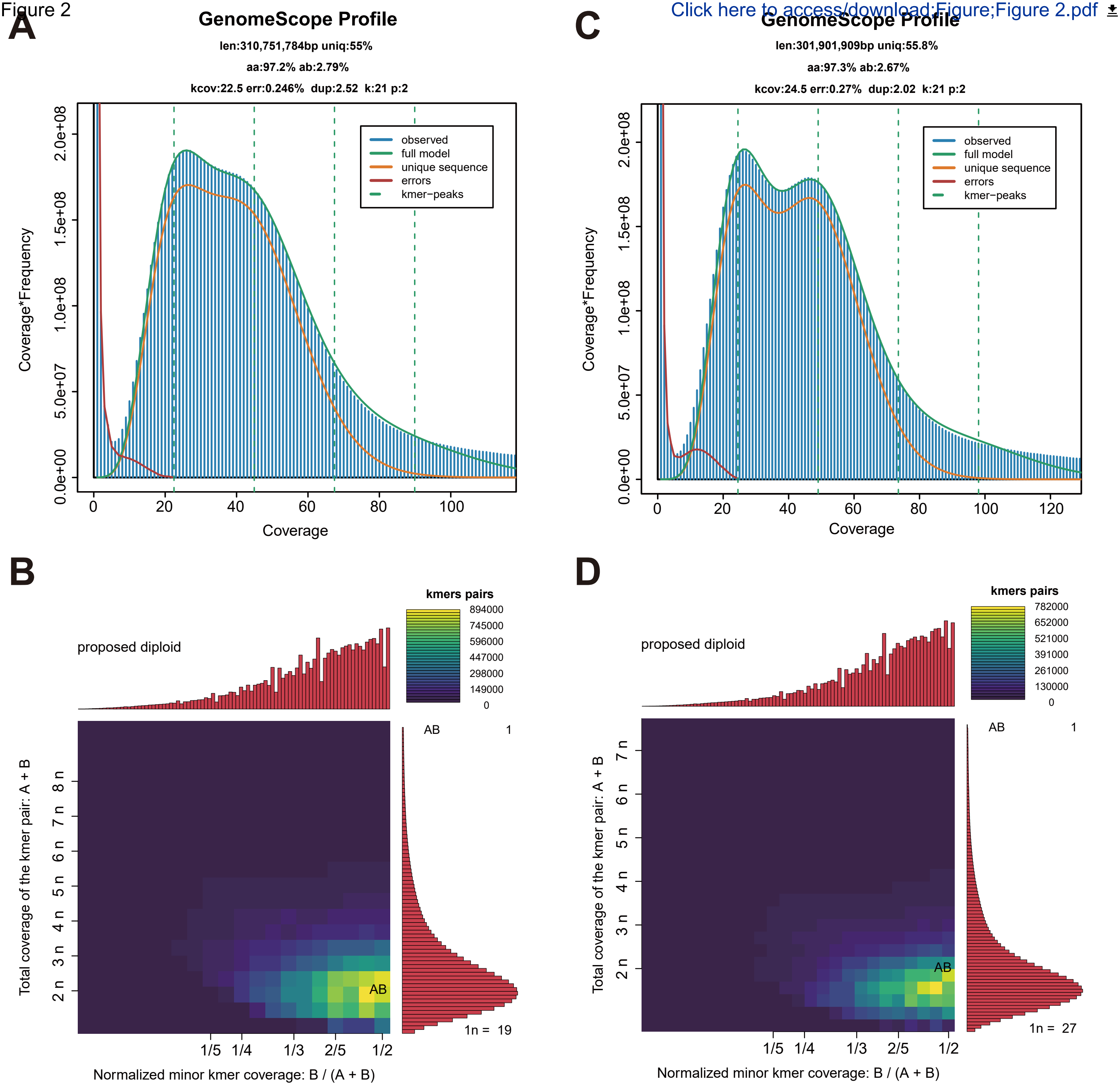

Figure 3

Click here to access/download;Figure;Figure 3.pdf

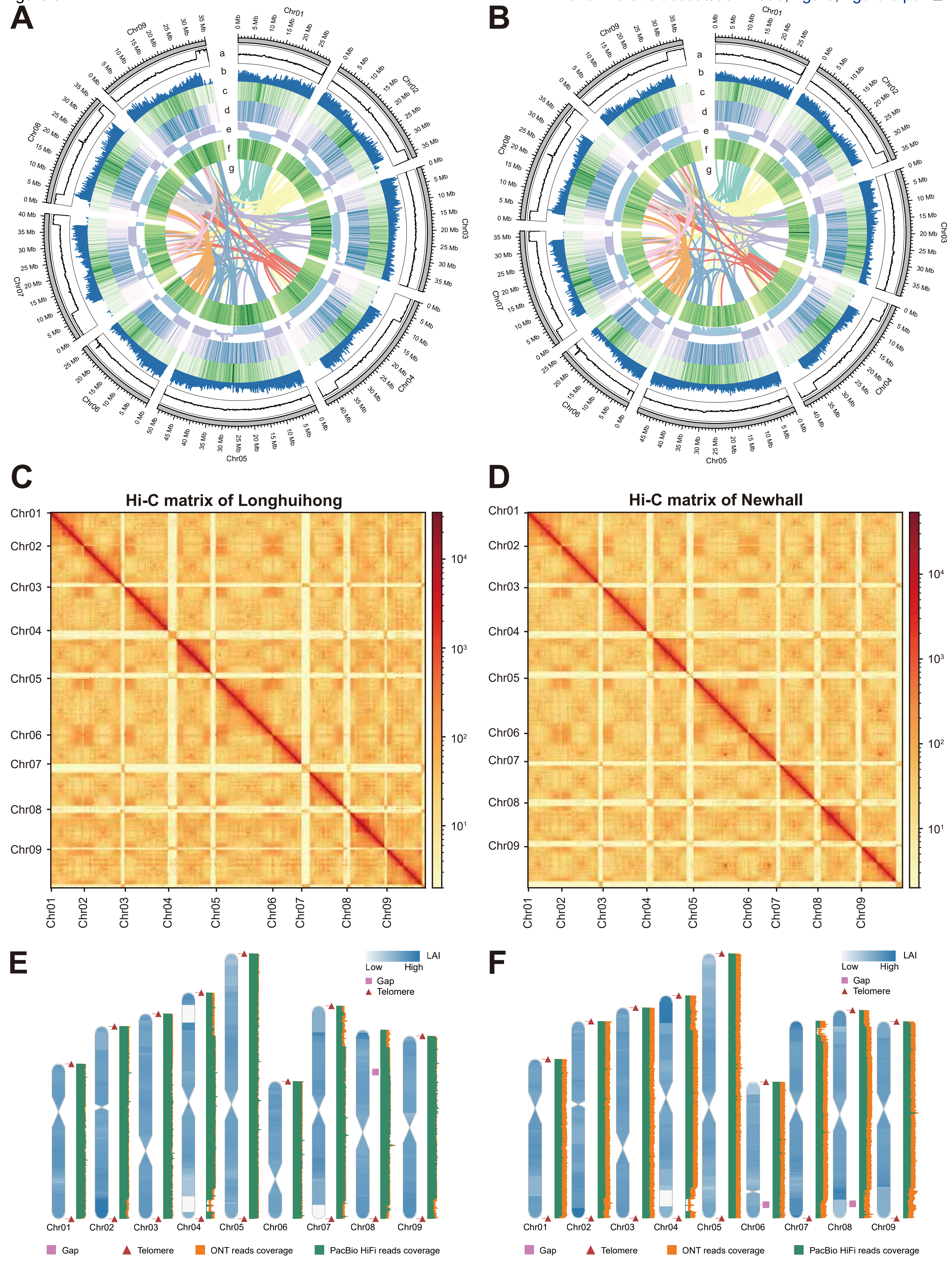

Figure 4

Click here to access/download;Figure;Figure 4.pdf

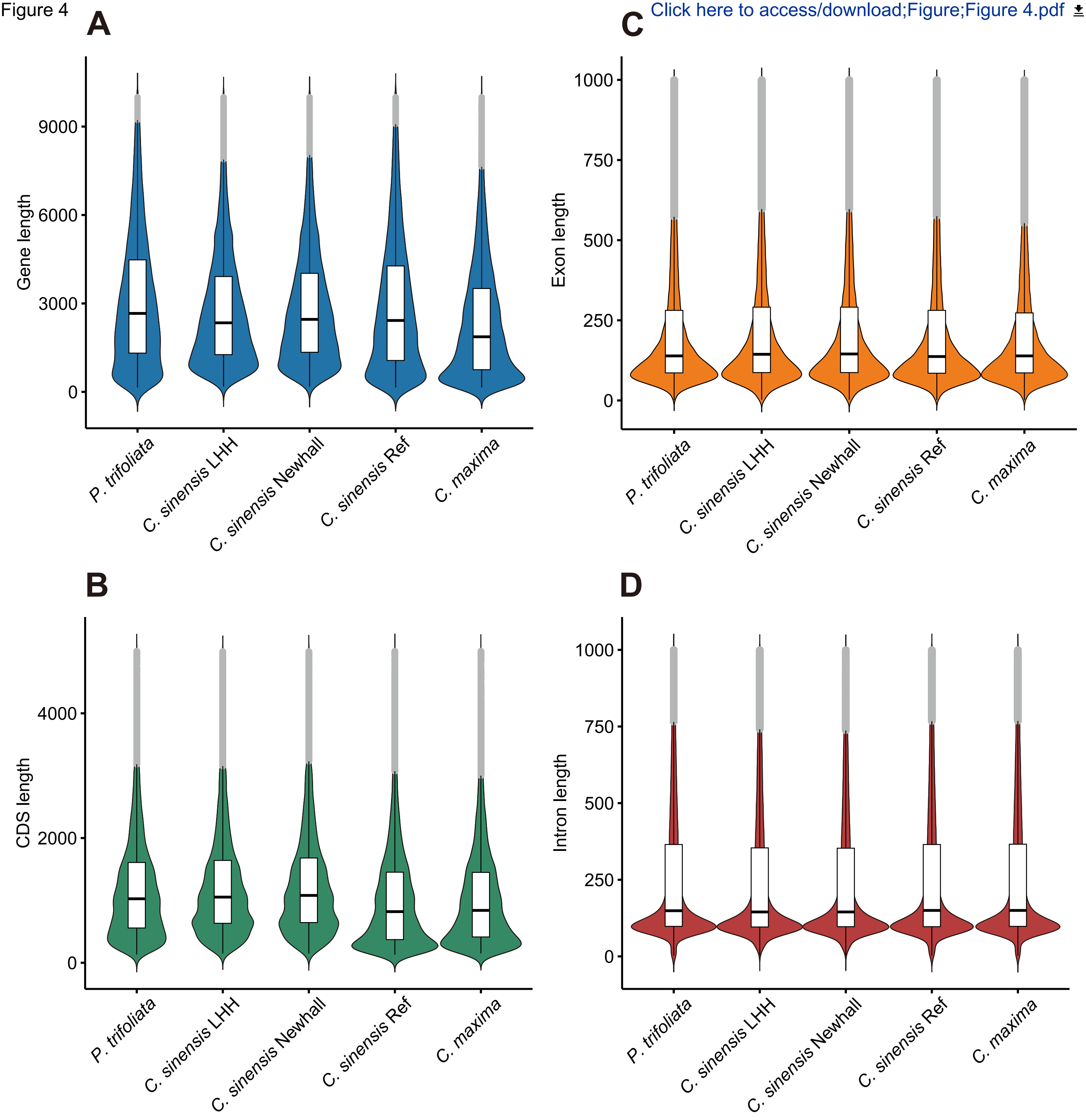

Figure 5

Click here to access/download;Figure;Figure 5.pdf

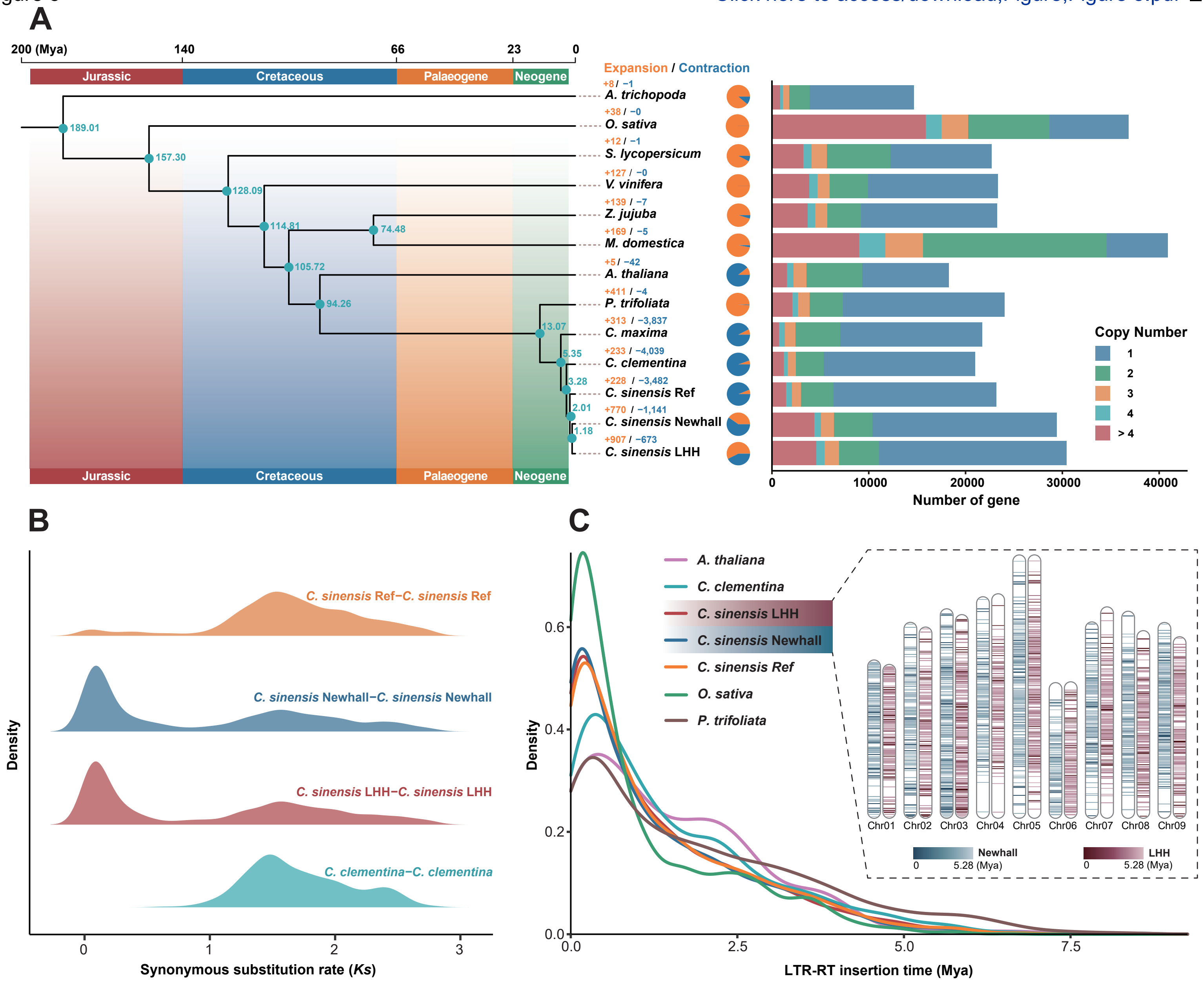

Figure 6
Click here to access/download;Figure;Figure 6.pdf

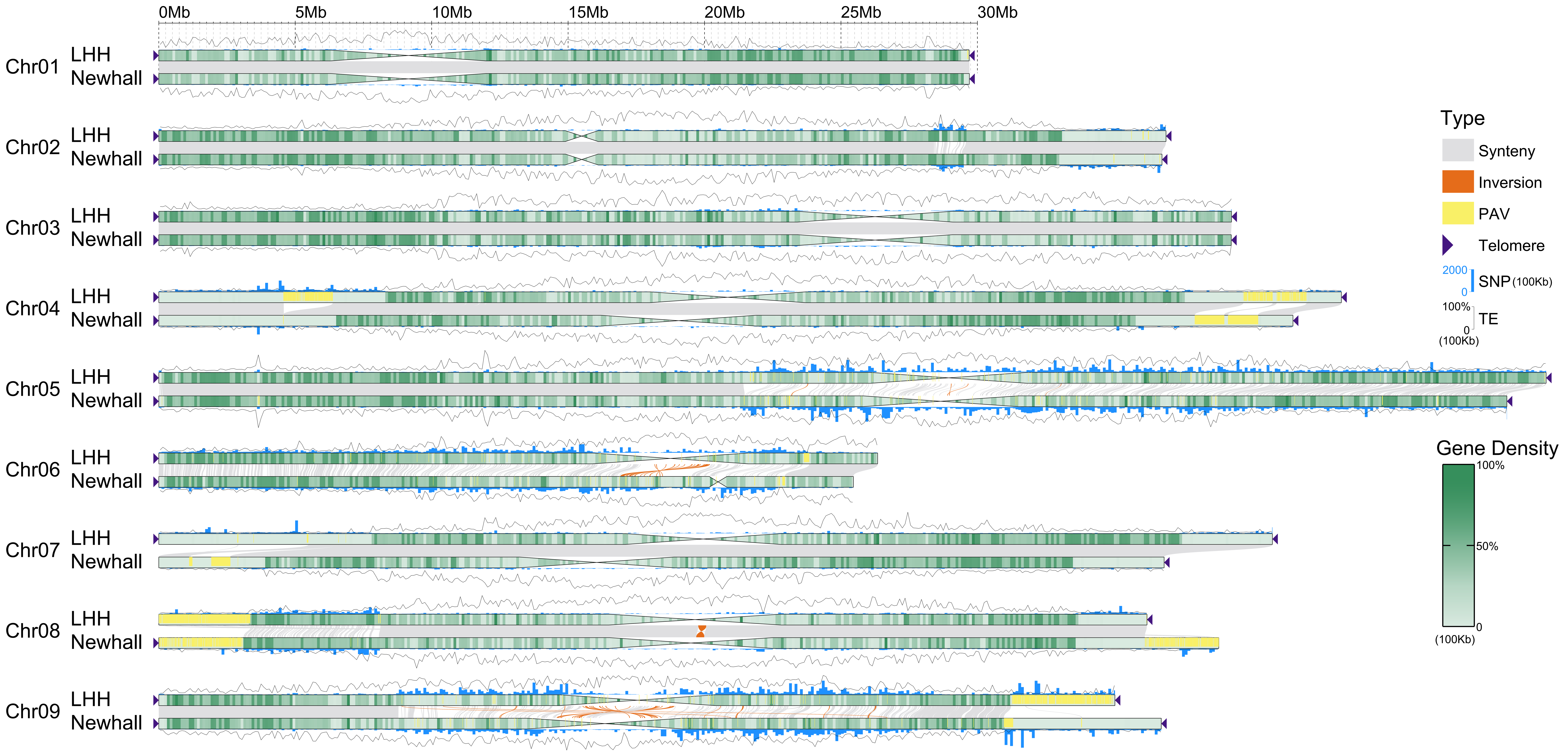

Supplementary Tables

Click here to access/download

Supplementary Material

Supplementary Tables 1-10.xlsx

Supplementary Figures

Click here to access/download
Supplementary Material
Supplementary Figure 1_5.pdf

Personal Cover Click here to access/download;Personal Cover;cover letter-MS-Gigascience-jms.docx

Dear Editor,

We are pleased to submit our manuscript entitled "**Construction and analysis of telomere-to-telomere genomes for two sweet oranges: Longhuihong and Newhall (*Citrus sinensis*)**" for consideration for publication in *GigaScience*.

Sweet orange (*Citrus sinensis* Osbeck) is a highly nutritious fruit crop widely consumed across the globe. However, sweet orange is particularly susceptible to low-temperature stress, which significantly impedes the sustainable development of the sweet orange industry. Therefore, developing cold-resistant sweet orange germplasm is crucial. Unfortunately, the lack of genomic resources for cold-resistant sweet orange varieties has hindered both the development of these varieties and the study of their molecular mechanisms of cold resistance.

In this study, we report the telomere-to-telomere (T2T) genomes of two sweet orange varieties with differing levels of cold resistance. By analyzing the differences between these genomes, we provide a data foundation for advancing research on cold resistance in sweet oranges.

The assembled sweet orange genomes underwent repeat and functional annotation. Comparative genomic analysis has revealed several significant events that have occurred in the genomes of *C. sinensis* LHH and Newhall, such as whole-genome triplication (WGT), expansion and contraction of gene families, and amplification of LTR-retrotransposons (LTR-RTs). Moreover, we identified and classified variations between the two sweet orange genomes. Notably, we observed structural variations in the introns and upstream/downstream regions of homologous genes related to cold resistance, previously reported in rice.

In summary, our study offers invaluable genomic resources for multi-omics and molecular biology research on sweet oranges. It also lays a data foundation for molecular breeding efforts aimed at improving cold resistance in sweet oranges. We believe our findings will capture the interest of a broad audience and make a meaningful contribution to the field.

Thank you for considering our manuscript for publication in *GigaScience*.

Sincerely,

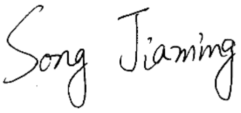

Dr. Jia-Ming Song

Professor, Southwest University, Chongqing, China 400715

E-mails address: jmsong@swu.edu.cn

On behalf of Dr. Lin Hong

Fruit Tree Research Institute, Chongqing Academy of Agricultural Sciences, Chongqing, China 401329
