# Supplementary material for: Construction and analysis of telomere-to-telomere genomes for 2 sweet oranges: Longhuihong and Newhall (Citrus sinensis)
Source: Gigascience. 2024 Nov 26;13:giae084. doi: 10.1093/gigascience/giae084 (PMC11590112; doi:10.1093/gigascience/giae084)
Supplement: giae084_Response_to_Reviewer_Comments_Original_Submission [file giae084_response_to_reviewer_comments_original_submission.pdf]

Author's Response To Reviewer Comments

Point-by-point response to reviewers' comments
We sincerely appreciate the reviewers for their insightful comments. We have thoroughly reviewed their feedback and made the necessary revisions to the manuscript. All modifications have been highlighted throughout both the revised manuscript and the figures.
Reviewer #1:
This study has generated two high quality collapsed genomes for two varieties of navel oranges; LHH and Newhall. They have performed the genome assemblies, annotations, structural variations detections, comparison of these two genomes with respect to other available genomes, and WGD analysis. Overall, this work provides valuable genomic resource for future research on sweet orange and other citrus as well. However, the methodology is not clearly and comprehensively explained for each analysis that have been performed here. For ex: If you use a specific tool for a particular analysis, it's always good to mention which type of input data you used (genome assemblies/whole protein or CDS files/the longest protein/CDS files), what are the steps of that analysis, specific threshold values (BLAST e-values).
Also, it would be good to indicate which tools the authors used to generate the figures in the figure legends. You can give the tool name and the reference as well. You also can mention it in the methodology and this is important to replicate the things in the future for another research.
some clarifications are required as listed below.
1. It would be good if you can give details about the method of leaf collections for DNA and RNA extractions, and Hi-C experiments for the other readers to know and replicate them in the future. (ex: collection under dry ice or leaves snap frozen)
[Response]: We genuinely appreciate your invaluable suggestions. In response, we have incorporated detailed descriptions of these experimental procedures into the methods section of our manuscript. The revised methodology is outlined below:
Healthy, juvenile leaves are carefully selected and their surfaces are thoroughly rinsed with deionized water. After gently drying the leaves to eliminate surface moisture, we precisely cut the tissue into 50-100 mg fragments. These fragments are then placed into pre-prepared 2 mL cryovials and promptly frozen in liquid nitrogen for a duration of 3-4 hours. Following this, the samples are securely stored at -80°C for future DNA and RNA extraction.
Hi-C experiment protocol:
(1) Collect 1 gram of tender leaves, rinse them thoroughly in icy water, and gently blot them dry with absorbent paper.
(2) Using scissors, carefully mince the sample tissue and place it into a 50 mL centrifuge tube.
(3) Transfer the minced tissue into a 50 mL centrifuge tube containing 35 mL of NIB Buffer.
(4) Introduce 35 μL of PMSF, 35 μL of β-mercaptoethanol, and 2 mL of 36% formaldehyde into the mixture.
(5) Allow the mixture to react for 90 minutes on a vertical rotator.
(6) Add 2.5 mL of 2M glycine and gently agitate the solution, either manually or on a shaker, for a minimum of 5 minutes to halt the reaction.
(7) Filter out the excess liquid and rinse the sample thoroughly with sterile water until all foam disappears.
(8) Blot the sample dry with absorbent paper, place it in a 50 mL centrifuge tube, and promptly freeze it in liquid nitrogen for future use.
2. Which method you used for DNA extraction? Was it CTAB? that was not mentioned in the manuscript.
[Response]: Thank you for pointing out this issue. The DNA extraction method used in our study is indeed CTAB. We have updated the manuscript to include this information and apologize for the omission.
3. Line 188 – It would be good to indicate the exact version of hifiasm you used for assemblies because each new release has fixed different issues in assemblies and has incorporated new improvements.
[Response]: We sincerely appreciate your feedback. In response, we have included and highlighted the version information for hifiasm (v0.19.8) [1] in the relevant section of our manuscript.
4. Any reason why you didn't generate haplotype-resolved assemblies for these two genomes? HiFiasm can integrate HiC data to give fully phased haplotypes and the haplotype-resolved assemblies are important in detecting the allele specific structural variations in genomes rather than collapsed genomes. The availability of Hi-C data is an added advantage of resolving the phased haplotypes.
[Response]: Thank you sincerely for providing valuable suggestions. Based on your suggestion, we have

included the assemblies of the LHH and Newhall haploid genomes (Fig. R1). Using the haplotype-resolved genome assemblies, we have further identified allele-specific structural variations, with a total of 164 such structural variants identified.

Figure R1: Synteny between the LHH and Newhall diploid genomes (https://cbi.gxu.edu.cn/zwzhou/xdxu/Figures/Figure_R1.pdf).

5. Line 213 – 216 You have indicated that "Telomeric sequences were detected at both ends of 7 chromosomes in each genome, while the remaining chromosomes exhibited telomeric sequences at one end.". If that is the case, how you define that these assemblies are T2T level without having telomeric repeats at both ends of all the chromosomes? It seems that two chromosomes of the two varieties don't have telomeric repeat at one end.
[Response]: We sincerely appreciate your valuable suggestions for our research. In this paper, the definition of the T2T genome is based on the recent publication [2], where a reference genome is considered T2T when more than half of its chromosomes have reached the T2T level. It is upon this criterion that we have defined the reference genome used in our study as a T2T genome.
6. Line 240-242 Is there a specific threshold in Merqury tool to identify the completeness of the assemblies? What is the minimum QV value for a complete genome?
[Response]: Thank you for raising valuable questions about our work. According to definitions in the literature, a higher assembly consensus quality value (QV) indicates greater accuracy, with Q30 corresponding to 99.9% accuracy and Q40 to 99.99% accuracy [3]. In our study, the QV of the assembled LHH and Newhall genomes reached 46.64 and 38.89, respectively, thus reflecting high accuracy.
7. Figure 3 E, F images – Some chromosomes have white regions within them. What these white regions indicate?
[Response]: We sincerely appreciate the questions raised about our research. The white areas in Figures 3E and 3F are due to weaker Hi-C contact signals in these regions compared to others. These regions, observed to be located near telomeres, may have lower Hi-C data coverage. However, upon inspection, these regions had already achieved chromosome-level assembly in the initial contig assembly. Therefore, Hi-C contact information was not used for the assembly of these regions, and their accuracy is not affected.
8. Table S1-S3, S5, S6, S8 Citrus sineses – spelling mistakes
[Response]: Thank you very much for pointing out this error. We have corrected the issue in the relevant sections and thoroughly reviewed the manuscript content. We apologize for any inconvenience this may have caused.
9. Figure 4 – What is the C. sinensis ref? haven't mentioned which genome used as the reference.
[Response]: We sincerely appreciate you pointing out the missing information. In Figure 4, "C. sinensis ref" refers to the sweet orange reference genome obtained from the NCBI database, with the corresponding RefSeq ID GCF_022201045.2. We have added this information to the revised manuscript and highlighted it accordingly. We deeply apologize for the oversight.
10. This manuscript does not have separate sections clearly mentioned as the background, Methodology, and Results. Please adhere to the journal guidelines. The methods and results have been combined under the sub topics of "Genome survey, Genome assembly and assessment, genome annotation).
[Response]: Thank you sincerely for providing valuable suggestions. In accordance with your suggestions, we have made the necessary adjustments to the structure of the manuscript and sincerely apologize for the structural issues present in the article.
11. The authors have selected the gene models coming from the final integration of different gene annotation methods (Table S7). However, it is not clear how the integration was done. Was there a specific software or a tool or was it done manually? This information was not found in a methodology section. Please explain. It's better to have these materials in the methodology section.
[Response]: We sincerely appreciate you pointing out the missing information in our manuscript. In this study, EVidenceModeler (v1.1.1) [4] was used to merge multiple versions of genome annotations. We have added this information to the Materials and Methods section of the manuscript and highlighted it accordingly. We deeply apologize for the omission of this detail.
12. Lines 301-302 What's the basis of using other non-citrus genomes for this analysis? The comparison among citrus genomes makes sense. It would be good to know what do you expect by comparing citrus genomes with other genomes. Also, on which basis did you select those genome assemblies? Are they high quality? Because all the subsequent analysis such as gene family clustering, expansion and contraction etc.

depend upon the accuracy and the completeness of the assemblies. Therefore, it’s always safe and good to use high quality, highly contiguous, complete genomes for any comparative analysis to get accurate results.
[Response]: Thank you for your valuable questions regarding our work. The inclusion of multiple non-Citrus species aims to use the known divergence times between these species to provide a more accurate estimation of the divergence times among the Citrus species of interest. Additionally, this approach enhances the accuracy and reliability of gene family clustering and the analysis of gene family expansion and contraction.
13. Line 316, 318 P. trifoliata should be Italy sized.
[Response]: We sincerely appreciate you pointing out this issue. We have made the necessary corrections at the relevant locations in the manuscript and have reviewed the entire document. The modified sections have been highlighted. We deeply apologize for any inconvenience caused.
14. A clear and comprehensive description of the methodology for CAFÉ analysis and the WGD analysis should be provided. Which tools you used and the steps you followed would be important in enabling the replication of work for others.
[Response]: We sincerely appreciate your valuable suggestions regarding our work. Based on your recommendations, we have made the necessary additions. In this study, the Computational Analysis of gene Family Evolution (CAFÉ) software (v4.2) [5] was used to estimate the number of gene family members in ancestral branches through the birth-death (λ) model, based on phylogenetic trees with divergence times and gene family clustering results. This approach allowed us to predict the contraction and expansion of gene families relative to ancestors in each species. The criteria for defining significant expansion or contraction in this study are that both family-wide P-values and Viterbi P-values are less than 0.05. Additionally, the WGD (v1.1.1) [6] software was used for analyzing whole-genome duplication (WGD) events.
15. Line 325 - Ancient polyploidization event which was common to all eudicots is a WGT event. All studies for genome assemblies of common citrus (Wu et al 2014 – C. clementina, Feng 2021 and Xu 2013 – C. sinensis), other Australian citrus genomes (Nakandala, 2024) and related species such as P. trifoliata (Peng 2021) revealed only one common, ancient WGT event. So, it would be good if you can make sure the results are correct.
[Response]: Thank you sincerely for providing valuable suggestions. Based on your suggestion, we have re-examined the whole-genome duplication (WGD) events in the two sweet orange genomes. Our analysis revealed that the first peak was caused by repetitive sequences in sweet orange rather than a WGD peak. This situation has also been observed in previous studies of Poncirus trifoliata [7]. Therefore, consistent with earlier reports, our results also support that the sweet orange genome has undergone only one whole-genome triplication (WGT) event (Fig. R2). We sincerely appreciate you pointing out this issue. We have made the necessary revisions in the manuscript, highlighted the changes, and apologize for any inconvenience caused.

Figure R2: The distribution of Ks values illustrates WGT events in the evolution of the C. sinensis LHH and Newhall genomes (https://cbi.gxu.edu.cn/zwzhou/xdxu/Figures/Figure_R2.pdf).

16. The figure 5B indicates the Ks plots for two species. Was it based on orthologous genes between the two genomes? Usually, the polyploidization events for a particular genome is based on the paralogous gene pairs of that particular species (self-genome comparison). All the other previous studies have revealed species-specific WGD events using Ks plots of paralogous gene pairs of that particular species. The orthologous ks peaks for two species might give information about the divergence events between those two species, but not the WGD events of a particular species.
[Response]: Thank you sincerely for providing valuable suggestions. We sincerely appreciate you pointing out this issue. Based on your suggestion, we have removed the Ks plots of orthologous gene pairs that are unrelated to WGT events in Figure 5 (Fig. R2). We apologize for any inconvenience this may have caused.

Figure R2: The distribution of Ks values illustrates WGT events in the evolution of the C. sinensis LHH and Newhall genomes (https://cbi.gxu.edu.cn/zwzhou/xdxu/Figures/Figure_R2.pdf).

17. Lines 329-331 – Do you have any explanation for the implications of the LTR insertions in these

genomes? What is the purpose of finding the LTR element insertions? For ex: does it have a relationship with genome size expansion of these genomes?
[Response]: Thank you sincerely for providing valuable suggestions. In response to your suggestions, we have provided additional explanations for this section of the manuscript. The identification of LTR insertions aims to explore the distribution landscape and evolutionary dynamics of LTRs in the LHH and Newhall genomes. In the three C. sinensis genomes, a higher density of recent LTR-RT insertions was observed compared to its closely related species, P. trifoliata. This increased density may have contributed to the larger genome size of C. sinensis relative to P. trifoliata [8].
18. Line 354 – The authors have not mentioned how the collinearity blocks were detected. There are specific tools like MCScanX to detect the collinear genes. Did you use such a tool? or was it based on Mummer alignments? Mummer does the alignment between two genomes and other types of tools are required to detect the synteny (ex: syri) or collinearity (MCScanX). Please elaborate how you performed the collinearity analysis. Ex: MCSCanX based collinearity requires protein fasta files for the detection of collinear genes, but not the genome assembly files. Also, collinearity is a special type of synteny which defines the same genes in the same order in the homologous chromosomes of two genomes.
[Response]: Thank you sincerely for providing valuable suggestions. Thank you very much for pointing out the missing information. In this study, we used GenomeSyn [9] for genome synteny analysis. This tool is based on the alignment results of the LHH and Newhall genomes using Mummer (v4.0.0beta2) [10] to construct and visualize syntenic regions between the two genomes. Following your suggestion, we have added the synteny analysis based on the gene sequences of LHH and Newhall (Fig. R3). This analysis was performed using MCScan-Python (https://github.com/tanghaibao/jcvi/wiki/MCscan-(Python-version)).

Figure R3: Synteny analysis between the LHH and Newhall genomes (https://cbi.gxu.edu.cn/zwzhou/xdxu/Figures/Figure_R3.pdf).

19. If the homologous gene identification was based on BLASTp, which e value was used?
[Response]: Thank you very much for pointing out the missing information. In the identification of homologous genes based on BLASTp [11], we used an e-value of 1e-5. We have updated the manuscript to include this information and sincerely apologize for the omission.
20. Figure 6B and 6C – It's not clear how these two images present the SVs between the two genomes. Do these bar graphs represent the number (Figure 6A) and the percentage (Figure 6C) of SVs of the two genomes or in one genome?
[Response]: Thank you very much for your valuable question. Figures 6A and 6C illustrate the structural variations between the LHH and Newhall T2T genomes. Figure 6A presents the types and counts of structural variations between homologous chromosomes of the LHH and Newhall T2T genomes. Figure 6B shows the percentage of structural variations located at different positions on homologous chromosomes, relative to the total number of structural variations between these chromosomes in the LHH and Newhall T2T genomes.
21. Line 422 – What are the differences in between the two varieties in terms of flesh color and photosynthetic efficiency?
[Response]: Thank you for your valuable questions regarding our research. In terms of flesh color, under the same cultivation conditions, the 'Longhuihong' fruit exhibits a distinct red color in the juice cells (indicative of anthocyanin accumulation), while 'Newhall' juice cells are a standard orange-yellow. Regarding photosynthetic rates, the parameters for 'Longhuihong' spring shoot leaves are significantly higher compared to those of 'Newhall', with increases of 33.4% in net photosynthetic rate, 55.6% in stomatal conductance, 20.9% in intercellular CO_2 concentration, and 33.1% in transpiration rate. Analysis of variance (ANOVA) indicates that the differences in photosynthetic parameters between the two varieties are statistically significant [12].
22. Don't you have any phenotypic or expression data for these two varieties?
[Response]: Thank you sincerely for providing valuable suggestions. Based on your suggestion, we have added the phenotypic information for the two varieties. In terms of photosynthetic rates, the parameters for 'Longhuihong' spring shoot leaves are significantly higher compared to 'Newhall', with increases of 33.4% in net photosynthetic rate, 55.6% in stomatal conductance, 20.9% in intercellular CO_2 concentration, and 33.1% in transpiration rate. Analysis of variance (ANOVA) indicates that the differences in these photosynthetic parameters between the two varieties are statistically significant [12].
In this study, RNA sequencing was performed on pooled tissues from both varieties to obtain gene

expression information. However, since the primary focus of this research is on the assembly and comparative genomics analysis of the two sweet orange genomes, our RNA-seq data were only utilized for gene annotation based on transcript evidence.

23. The raw sequence data could be found in the given NCBI bioproject. Please give the accession numbers of the deposited genome assemblies and annotations in NCBI or other databases. Only the bioproject number will not be sufficient for the others to get access to this data in the future.

[Response]: Thank you sincerely for providing valuable suggestions. In response to your suggestion, we have updated the data availability section of the manuscript with detailed information on the publicly available data. The PacBio HiFi, Hi-C, ONT UL, Illumina PE, and RNA-seq sequencing data for this project can be accessed through NCBI BioProject PRJNA1122682, with corresponding numbers SRR29362821 to SRR29362830 (Table R1). The LHH and Newhall genomes have been deposited in DDBJ/ENA/GenBank under accession numbers JBFBVJ000000000 and JBFBVK000000000, respectively.

Table R1. NCBI accession numbers for LHH and Newhall genome sequencing data

Accession	Title
SRR29362821	Newhall RNA-Seq
SRR29362822	LHH RNA-Seq
SRR29362823	Newhall Illumina PE
SRR29362824	Newhall Hi-C
SRR29362825	Newhall ONT UL
SRR29362826	Newhall PacBio HiFi
SRR29362827	LHH Illumina PE
SRR29362828	LHH Hi-C
SRR29362829	LHH ONT UL
SRR29362830	LHH PacBio HiFi

Reviewer #2:

This is a study to construct the entire genome of two sweet orange mutant strains. This research is important for understanding the cold-resistance of sweet oranges, which is the ultimate goal. The objectives and approach of the study are appropriate and rationale. Genome assembly follows standard procedures, and the analysis of SV and functional annotation were also performed according to standard procedures. The BUSCO score suggests that the quality of the assembled genome is high, and it also indicates that the full-length sequence retains telomere structure at both ends of the chromosome.

While the results presented are generally understandable, some descriptions are unclear, some seem erroneous, and some require confirmation. The following is a list of such cases. Please check them and correct them appropriately.

1. Although the amounts of reads used in the analysis of the two sweet orange mutant lines tested are almost the same, discrepancies are observed in the assembly size (Table 2; 361.41 Mb/370.23 Mb) and estimated gene number (31,456/30,021). This study identified 2,886 structural variants between these two genomes; however, I feel that number is quite large as they are mutants.

Since many studies take a similar approach, I highly encourage authors to also discuss possible problems with the genome assembly process.

[Response]: Thank you for your valuable suggestions regarding our research. Based on your advice, we have reviewed the genome assembly and confirmed that the variations observed were not due to assembly errors but rather due to sequence differences between the haploid genomes. Consequently, we have included genome assemblies specific to the LHH and Newhall haplotypes (Fig. R1) and identified haplotype-specific structural variations. Our analysis detected a total of 164 haplotype-specific structural variations. This information has been incorporated into the manuscript.

Figure R1: Synteny between the LHH and Newhall diploid genomes (https://cbi.gxu.edu.cn/zwzhou/xdxu/Figures/Figure_R1.pdf).

2. Figure 3. E and F: Why are the estimated positions of CENs different in Chr 01 and 08 between these two assemblies? Any possible causes, if any, should be indicated in the results and discussion.

[Response]: Thank you for your valuable suggestions regarding our research. In response to the issues you

pointed out, we used the recently published CentIER (v3.0) to re-evaluate the centromere regions. This tool has been reported to outperform similar software by more than 20% in various accuracy prediction metrics [13]. Through this re-evaluation, centromeres on multiple chromosomes, including Chr 01 and Chr 08, have been corrected (Fig. R4). We have updated the relevant information in Figure 3E, F, and the supplementary materials of the manuscript.

Figure R4: The distribution landscape of centromeres, telomeres, LAI, HiFi reads coverage, and ONT reads coverage in the LHH genome (A) and Newhall genome (B) (https://cbi.gxu.edu.cn/zwzhou/xdxu/Figures/Figure_R4.pdf).

3. Also, in Figure 3F, almost the entire length of the assembled chromosome appears to be covered by the ONT reads, while in Figure 3E, only some regions appear to be covered by the ONT reads. This is not consistent with the description in text L226-228.
[Response]: Thank you for pointing out this issue. The coverage depicted in Figure E does not indicate that ONT reads are specifically targeting localized regions. Instead, both HiFi and ONT reads cover most of the regions, with similar coverage levels, leading to overlap. Figure F illustrates that the higher coverage observed for ONT reads is consistent with the sequencing data statistics: the ONT read coverage for the LHH genome is 51×, whereas for the Newhall genome, it is 61×.
4. Figure 6 is too large; I encourage splitting A, B, and C into single figures and providing B and C as supplements.
[Response]: Thank you for your valuable suggestions regarding our research. We have revised Figure 6 as requested and moved panels 6B and 6C to the supplementary figures section.
5. Although the similarities between the two assembled orange genomes are shown in Figure 6A, it is difficult to understand the correspondence between these two genomes. Please also add a Matrix plot (mummer plot) of all chromosomes.
[Response]: Thank you for your suggestions on our article. Following your advice, we have added a matrix plot (MUMmer plot) showing the genome comparison results to the supplementary figures (Fig. R5).

Figure R5: Dot matrix comparison of the C. sinensis LHH and Newhall genomes (https://cbi.gxu.edu.cn/zwzhou/xdxu/Figures/Figure_R5.pdf). The x-axis represents the LHH genome coordinates, and the y-axis represents the Newhall genome coordinates.

6. Figure 6A What do the pale green triangles at the chromosome ends of Chr02, Chr04, Chr07, Chr08, and Chr09 mean? Also, how were the SNPs shown here called?
[Response]: Thank you for your suggestions on our research. The triangular markers at the chromosome ends indicate the presence of telomeres at these locations. The SNP calling method used in this study is as follows: Minimap2 (v2.26-r1175) [14] was employed to align the PacBio HiFi reads of the LHH genome to the Newhall genome. Subsequently, DeepVariant (v1.6.1) [15] was used for SNP detection from the alignment results. Heterozygous SNPs identified in the SNP calling results were filtered out, resulting in the final dataset used for presentation. We have supplemented this information in the Materials and Methods section of the manuscript and sincerely apologize for the omission.
7. L72: This sentence says that about 80% of sweet oranges are derived from somatic mutations, but it is unclear on what basis. Does this 80% represent the percentage of sports and nucellar seedlings? If the remaining ca. 20% are neither sports nor nucellar seedlings, then they are sweet orange hybrids, not sweet oranges. This needs to be corrected.
[Response]: Thank you for your excellent suggestion. Currently, bud mutation selection is the primary source of cultivated sweet orange varieties. It is estimated that about 80% of sweet orange varieties originate from bud mutation selection based on somatic variation. Apart from bud mutation selection, the remaining 20% of sweet orange varieties are primarily developed through radiation and chemical mutagenesis methods.
8. L136: What do you mean for 'mid-to-high altitude'? Is this a proper noun?
[Response]: Thank you very much for raising questions about our research. We intended to convey the elevation of the experimental field. We have revised the manuscript to specify the exact elevation of the experimental field as 750 meters and sincerely apologize for any confusion caused by the previous

description.
9. L103 What do you mean 'perfume lemon'?
[Response]: Thank you for your questions regarding our research. The types of cultivated citrus varieties we typically refer to include sweet oranges, mandarins, grapefruits, lemons, and their hybrids. ‘Perfume lemon’ refers to a variety of lemon known for its distinctive fragrance.
10. L145 Reference is required for this protocol.
[Response]: Thank you for your valuable suggestions. In response to your comments, we have reviewed and corrected the relevant section of the manuscript, and we have added the appropriate references. The modified content has been highlighted for clarity. We sincerely apologize for the incomplete information in this section.
11. L157 Insert 'DNA' before 'concentration'.
[Response]: Thank you for your valuable suggestions on our article. Based on your recommendations, we have made the necessary revisions in the corresponding sections of the manuscript.
12. L178 I suppose this 'Fig. 1B, D' would be 'Fig. 2B, D'.
[Response]: Thank you very much for pointing out this issue with our manuscript. It should indeed be Fig. 2B, D. We have corrected and highlighted the changes in the relevant sections of the manuscript and have re-checked the document. We apologize for the labeling error.
13. L201 Is 'Fig. 2C' 'Fig. 3C'?
[Response]: Thank you very much for pointing out this issue in our manuscript. It should indeed be Fig. 3C. We have made the necessary corrections and highlighted the changes in the relevant sections of the manuscript. We have also carefully re-checked the document and apologize for the labeling error.
14. L279 Cite reference for those 'closely related species genomes'.
[Response]: Thank you for pointing out the missing information in our manuscript. Following your suggestion, we have added the references to the manuscript and apologize for the oversight regarding this information.
15. L289 714 and 3,920 rRNA, these numbers show a large discrepancy. Confirm them.
[Response]: Thank you for your valuable suggestions regarding our research. Following your advice, we have reviewed the identified rRNA quantity and can confirm that it is indeed 3,920.
16. L302 Please describe the names of those 13 species, respectively in parenthesis.
[Response]: Thank you for pointing out the missing information in our manuscript. Following your suggestion, we have added the full names of the 13 species to the manuscript and apologize for the oversight regarding this information.
17. L319 Did you use known calibration point for the divergence time estimation?
[Response]: Thank you for the valuable questions you have raised about our research. In our study of divergence events, we obtained fossil dates from the TimeTree website (http://www.timetree.org/): 179.9-204.9 Mya for A. trichopoda vs. C. maxima, 0.44-0.48 Mya for C. maxima vs. C. sinensis Ref, 1.47-5.73 Mya for C. maxima vs. C. clementina, and 90-100.5 Mya for C. maxima vs. A. thaliana. We have now included this information and the specific analysis methods in the manuscript, highlighting these additions. We apologize for the oversight regarding this information.
18. L329-331 This sentence looks strange. Navel orange is a mutant line of sweet orange, and sweet orange has been recognized to emerge around 2,000 years ago, not 5 Mya. Furthermore, Newhall is the selection of navel orange discovered in 1912 in UC Riverside. Revise this sentence sounds coincide with natural history of sweet orange and its mutant selections.
[Response]: Thank you for highlighting this issue with our research. We have removed the inappropriate expressions from our manuscript and sincerely apologize for these inaccuracies.
19. Figure 5B Describe the unit of Density (vert axis).
[Response]: Thank you for your suggestions regarding our research. The y-axis unit of the density plot does not have a specific physical unit because density is a relative measure. It represents the density value of the data at that point. Typically, it does not have a direct unit but can be understood as ‘the probability per unit interval.’
References
1. Cheng H, Concepcion GT, Feng X, Zhang H, Li H. Haplotype-resolved de novo assembly using phased assembly graphs with hifiasm. Nat Methods. 2021; doi: 10.1038/s41592-020-01056-5.
2. Xie L, Gong X, Yang K, Huang Y, Zhang S, Shen L, et al.. Technology-enabled great leap in deciphering plant genomes. Nat Plants. Nature Publishing Group; 2024; doi: 10.1038/s41477-024-01655-6.
3. Rhie A, Walenz BP, Koren S, Phillippy AM. Merqury: reference-free quality, completeness, and phasing assessment for genome assemblies. Genome Biol. 2020; doi: 10.1186/s13059-020-02134-9.

4. Haas BJ, Salzberg SL, Zhu W, Pertea M, Allen JE, Orvis J, et al.. Automated eukaryotic gene structure annotation using EVidenceModeler and the Program to Assemble Spliced Alignments. Genome Biol. 2008; doi: 10.1186/gb-2008-9-1-r7.
5. Han MV, Thomas GWC, Lugo-Martinez J, Hahn MW. Estimating gene gain and loss rates in the presence of error in genome assembly and annotation using CAFE 3. Mol Biol Evol. 2013; doi: 10.1093/molbev/mst100.
6. Zwaenepoel A, Van de Peer Y. wgd-simple command line tools for the analysis of ancient whole-genome duplications. Bioinforma Oxf Engl. 2019; doi: 10.1093/bioinformatics/bty915.
7. Peng Z, Bredeson JV, Wu GA, Shu S, Rawat N, Du D, et al.. A chromosome-scale reference genome of trifoliate orange (Poncirus trifoliata) provides insights into disease resistance, cold tolerance and genome evolution in Citrus. Plant J. 2020; doi: 10.1111/tpj.14993.
8. Zhang T, Qiao Q, Novikova PY, Wang Q, Yue J, Guan Y, et al.. Genome of Crucihimalaya himalaica, a close relative of Arabidopsis, shows ecological adaptation to high altitude. Proc Natl Acad Sci U S A. 2019; doi: 10.1073/pnas.1817580116.
9. Zhou Z-W, Yu Z-G, Huang X-M, Liu J-S, Guo Y-X, Chen L-L, et al.. GenomeSyn: a bioinformatics tool for visualizing genome synteny and structural variations. J Genet Genomics Yi Chuan Xue Bao. 2022; doi: 10.1016/j.jgg.2022.03.013.
10. Marçais G, Delcher AL, Phillippy AM, Coston R, Salzberg SL, Zimin A. MUMmer4: A fast and versatile genome alignment system. PLoS Comput Biol. 2018; doi: 10.1371/journal.pcbi.1005944.
11. Cock PJA, Chilton JM, Grüning B, Johnson JE, Soranzo N. NCBI BLAST+ integrated into Galaxy. GigaScience. 2015; doi: 10.1186/s13742-015-0080-7.
12. Yuan, Gaopeng, Chun, changpin, Peng, Liangzhi, Huang, Zongjun, Huang, Tongqi, Yang, Huidong, et al.. A Comparative Analysis of ‘Newhall’ Navel Orange and Its Bud Sport ‘Longhuihong.’ Fruit Tree J. 2017; doi: 10.13925/j.cnki.gsxb.20170096.
13. Xu D, Yang J, Wen H, Feng W, Zhang X, Hui X, et al.. CentIER: accurate centromere identification for plant genome. Plant Commun. Elsevier; 2024; doi: 10.1016/j.xplc.2024.101046.
14. Li H. Minimap2: pairwise alignment for nucleotide sequences. Bioinforma Oxf Engl. 2018; doi: 10.1093/bioinformatics/bty191.
15. Poplin R, Chang P-C, Alexander D, Schwartz S, Colthurst T, Ku A, et al.. A universal SNP and small-indel variant caller using deep neural networks. Nat Biotechnol. 2018; doi: 10.1038/nbt.4235.
