# Supplementary material for: Construction and analysis of telomere-to-telomere genomes for 2 sweet oranges: Longhuihong and Newhall (Citrus sinensis)
Source: Gigascience. 2024 Nov 26;13:giae084. doi: 10.1093/gigascience/giae084 (PMC11590112; doi:10.1093/gigascience/giae084)
Supplement: giae084_Reviewer_2_Report_Original_Submission — Tokurou Shimizu -- 7/23/2024 [file giae084_reviewer_2_report_original_submission.pdf]

Reviewer Report

Title: Construction and analysis of telomere-to-telomere genomes for two sweet oranges: Longhuihong and Newhall (Citrus sinensis)

Version: Original Submission **Date:** 7/23/2024

Reviewer name: Tokurou Shimizu

Reviewer Comments to Author:

This is a study to construct the entire genome of two sweet orange mutant strains. This research is important for understanding the cold-resistance of sweet oranges, which is the ultimate goal. The objectives and approach of the study are appropriate and rationale. Genome assembly follows standard procedures, and the analysis of SV and functional annotation were also performed according to standard procedures. The BUSCO score suggests that the quality of the assembled genome is high, and it also indicates that the full-length sequence retains telomere structure at both ends of the chromosome.While the results presented are generally understandable, some descriptions are unclear, some seem erroneous, and some require confirmation. The following is a list of such cases. Please check them and correct them appropriately.1. Although the amounts of reads used in the analysis of the two sweet orange mutant lines tested are almost the same, discrepancies are observed in the assembly size (Table 2; 361.41 Mb/370.23 Mb) and estimated gene number (31,456/30,021). This study identified 2,886 structural variants between these two genomes; however, I feel that number is quite large as they are mutants.Since many studies take a similar approach, I highly encourage authors to also discuss possible problems with the genome assembly process.2. Figure 3. E and F: Why are the estimated positions of CENs different in Chr 01 and 08 between these two assemblies? Any possible causes, if any, should be indicated in the results and discussion.3. Also, in Figure 3F, almost the entire length of the assembled chromosome appears to be covered by the ONT reads, while in Figure 3E, only some regions appear to be covered by the ONT reads. This is not consistent with the description in text L226-228.4. Figure 6 is too large; I encourage splitting A, B, and C into single figures and providing B and C as supplements.5. Although the similarities between the two assembled orange genomes are shown in Figure 6A, it is difficult to understand the correspondence between these two genomes. Please also add a Matrix plot (mummer plot) of all chromosomes.6. Figure 6A What do the pale green triangles at the chromosome ends of Chr02, Chr04, Chr07, Chr08, and Chr09 mean? Also, how were the SNPs shown here called?L72: This sentence says that about 80% of sweet oranges are derived from somatic mutations, but it is unclear on what basis. Does this 80% represent the percentage of sports and nucellar seedlings? If the remaining ca. 20% are neither sports nor nucellar seedlings, then they are sweet orange hybrids, not sweet oranges. This needs to be corrected.L136: What do you mean for 'mid-to-high altitude'? Is this a proper noun?L103 What do you mean 'perfume lemon'?L145 Reference is required for this protocol.L157 Insert 'DNA' before 'concentration'.L178 I suppose this 'Fig. 1B, D' would be 'Fig. 2B, D'.L201 Is 'Fig. 2C' 'Fig. 3C'?L279 Cite reference for those 'closely related species genomes'.L289 714 and 3,920 rRNA, these numbers show a large discrepancy. Confirm them.L302 Please describe the names of those 13 species, respectively in parenthesis.L319 Did you use known calibration point for the

divergence time estimation?L329-331 This sentence looks strange. Navel orange is a mutant line of sweet orange, and sweet orange has been recognized to emerge around 2,000 years ago, not 5 Mya. Furthermore, Newhall is the selection of navel orange discovered in 1912 in UC Riverside. Revise this sentence sounds coincide with natural history of sweet orange and its mutant selections.Figure 5B Describe the unit of Density (vert axis).

Level of Interest

Please indicate how interesting you found the manuscript: Choose an item.

Quality of Written English

Please indicate the quality of language in the manuscript: Choose an item.

Declaration of Competing Interests

Please complete a declaration of competing interests, considering the following questions:

- Have you in the past five years received reimbursements, fees, funding, or salary from an organisation that may in any way gain or lose financially from the publication of this manuscript, either now or in the future?
- Do you hold any stocks or shares in an organisation that may in any way gain or lose financially from the publication of this manuscript, either now or in the future?
- Do you hold or are you currently applying for any patents relating to the content of the manuscript?
- Have you received reimbursements, fees, funding, or salary from an organization that holds or has applied for patents relating to the content of the manuscript?
- Do you have any other financial competing interests?
- Do you have any non-financial competing interests in relation to this paper?

If you can answer no to all of the above, write 'I declare that I have no competing interests' below. If your reply is yes to any, please give details below.

I declare that I have no competing interests.

I agree to the open peer review policy of the journal. I understand that my name will be included on my report to the authors and, if the manuscript is accepted for publication, my named report including any attachments I upload will be posted on the website along with the authors' responses. I agree for my report to be made available under an Open Access Creative Commons CC-BY license (http://creativecommons.org/licenses/by/4.0/). I understand that any comments which I do not wish to be included in my named report can be included as confidential comments to the editors, which will not be published.

Choose an item.

To further support our reviewers, we have joined with Publons, where you can gain additional credit to further highlight your hard work (see: https://publons.com/journal/530/gigascience). On publication of this paper, your review will be automatically added to Publons, you can then choose whether or not to claim your Publons credit. I understand this statement.

Yes Choose an item.
